# Visible-Light-Mediated 1,2-Arylpyridylation of Alkenes Using Arylboronic Acids and Cyanopyridines

**DOI:** 10.3390/molecules31132216

**Published:** 2026-06-24

**Authors:** Zi-Wei Xi, Zhuo-Ying Dong, Shu-Qing Hu, Ke-Li Qiu, Li He, Yong-Miao Shen

**Affiliations:** 1 School of Chemistry and Chemical Engineering, Zhejiang Sci-Tech University, Hangzhou 310018, China; xiziwei@zstu.edu.cn (Z.-W.X.); 2024221002014@mails.zstu.edu.cn (Z.-Y.D.); 2025221002022@mails.zstu.edu.cn (S.-Q.H.); 2 Shengzhou Innovation Research Institute, Zhejiang Sci-Tech University, Shengzhou 312400, China; 2025221002023@mails.zstu.edu.cn (K.-L.Q.); 2025221002028@mails.zstu.edu.cn (L.H.)

**Keywords:** photocatalysis, 1,2-arylpyridylation, aryl radical

## Abstract

We report a visible-light-mediated photoreduction strategy for the three-component 1,2-arylpyridylation of alkenes with arylboronic acids and 4-cyanopyridines. The reaction mechanism was verified via radical trapping experiments and DFT calculations. This reaction proceeds under mild conditions, features broad substrate compatibility and is readily scalable to the gram scale.

## 1. Introduction

Pyridine derivatives are ubiquitous in natural products, pharmaceutical molecules, and functional materials [1,2,3], serving as core heterocyclic structures with great research value. Coupling reactions represent modular and efficient strategies for constructing Csp^2^-Csp^3^ bonds between pyridines and functionalized carbon-containing groups. A diverse array of synthetic routes is available to access pyridine derivatives, ranging from Minisci-type reactions [4,5] and transition-metal-catalyzed C–H activation [6,7,8,9,10] to photochemical synthetic protocols [11]. In recent years, radical-mediated synthetic strategies represented by photochemistry [12] and electrochemistry [13] have emerged as promising alternatives [14,15,16]. These protocols feature mild conditions, low cost, no need for pre-functionalization of substrates, and excellent functional group compatibility. Among them, visible-light-driven photoredox catalysis for alkene difunctionalization exhibits great application potential. It enables the activation of alkenes via single electron transfer (SET), introduces two functional groups in a single step and rapidly constructs diverse molecular frameworks, thus becoming an efficient new approach to alkene difunctionalization [17,18].

**Scheme 1:**
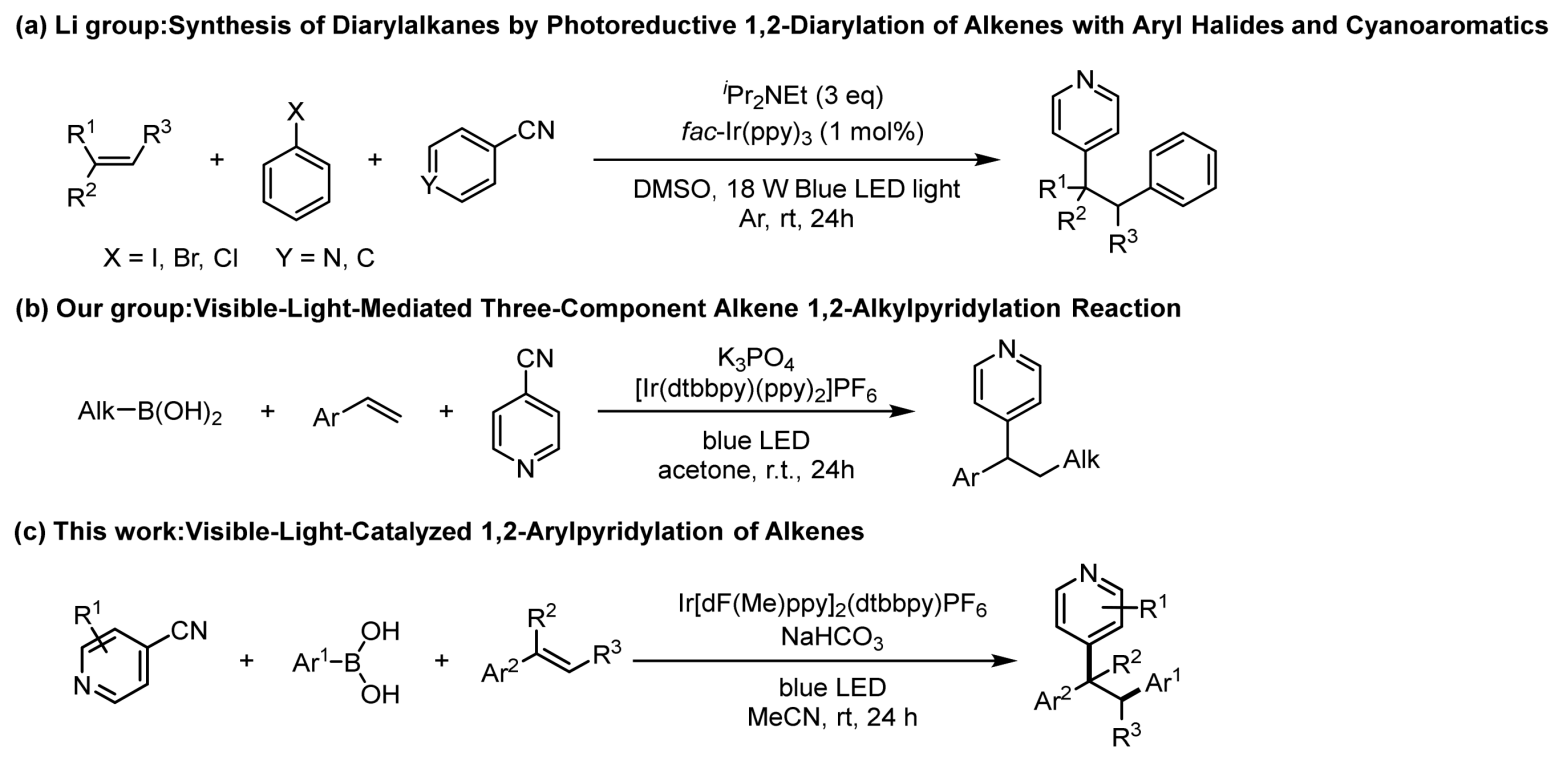
C-C bond is constructed from cyanopyridine as a pyridine source.

Over the past decade, innovative strategies including photocatalytic radical transformations and asymmetric difunctionalization [19,20] have developed rapidly, opening up new avenues for the precise synthesis of structurally complex and functionalized pyridine derivatives [21,22,23,24]. Typical examples include photocatalytic pyridyl-carbamoylation [25,26], pyridyl-alkylation [27], sulfonylative pyridylation [28], pyridyl-arylation [15,29], and 1,2-boroarylation and carbopyridylation [30] of alkenes. In 2024, Li’s group [29] developed a photocatalytic three-component reaction of aryl halides, cyanoarenes, and alkenes for the synthesis of 1,2-diarylated compounds, with the substrate scope extended to various cyanoarenes including electron-deficient benzonitriles and isonicotinonitriles (Scheme 1a). In 2025, our group [31] developed a visible-light-mediated three-component 1,2-alkylpyridylation of alkenes using alkylboronic acids as radical precursors for the construction of 4-alkylpyridines (Scheme 1b). Building on this work, we further established a photocatalytic protocol wherein arylboronic acids generate aryl radicals under light irradiation; subsequent radical addition to alkenes and radical-radical coupling with cyanopyridyl radical anions afford pyridine-containing 1,2-diarylated alkene derivatives (Scheme 1c).

## 2. Results and Discussion

### 2.1. Optimization of Reaction Conditions

We used 4-cyanopyridine (**1a**), 4-methylphenylboronic acid (**2a**), and styrene (**3a**) as model substrates. After an extensive screening of the reaction conditions (see Supporting Information (Supplementary Materials) for details), we found that using Ir[dF(Me)ppy]_2_(dtbbpy)PF_6_ (1.2 mol %) as the photocatalyst and NaHCO_3_ as the base, the reaction in acetonitrile (MeCN) proceeded under blue LED irradiation at room temperature for 24 h to afford product **4a** in 73% yield (Table 1, entry 20). Control experiments showed that both the light and photocatalyst were essential for the reaction (Table 1, entry 21 and 22), which established the photochemical character of the reaction. Furthermore, the yield decreased significantly when the reaction was carried out under an air atmosphere, demonstrating the necessity of an argon protective environment (Table 1, entry 23). Green light failed to drive the reaction (Table 1, entry 25); violet light accelerated the reaction rate but did not improve the product yield (Table 1, entry 24). We found that the reaction is accompanied by the formation of two-component byproducts originating from cyanopyridine and alkene. Increasing the loading of arylboronic acids promotes the generation of aryl radicals, which accelerates the reaction and inhibits the formation of this byproduct. Accordingly, a relatively high equivalent of boronic acid is adopted in this transformation.

### 2.2. Substrate Expansion

**Scheme 2:**
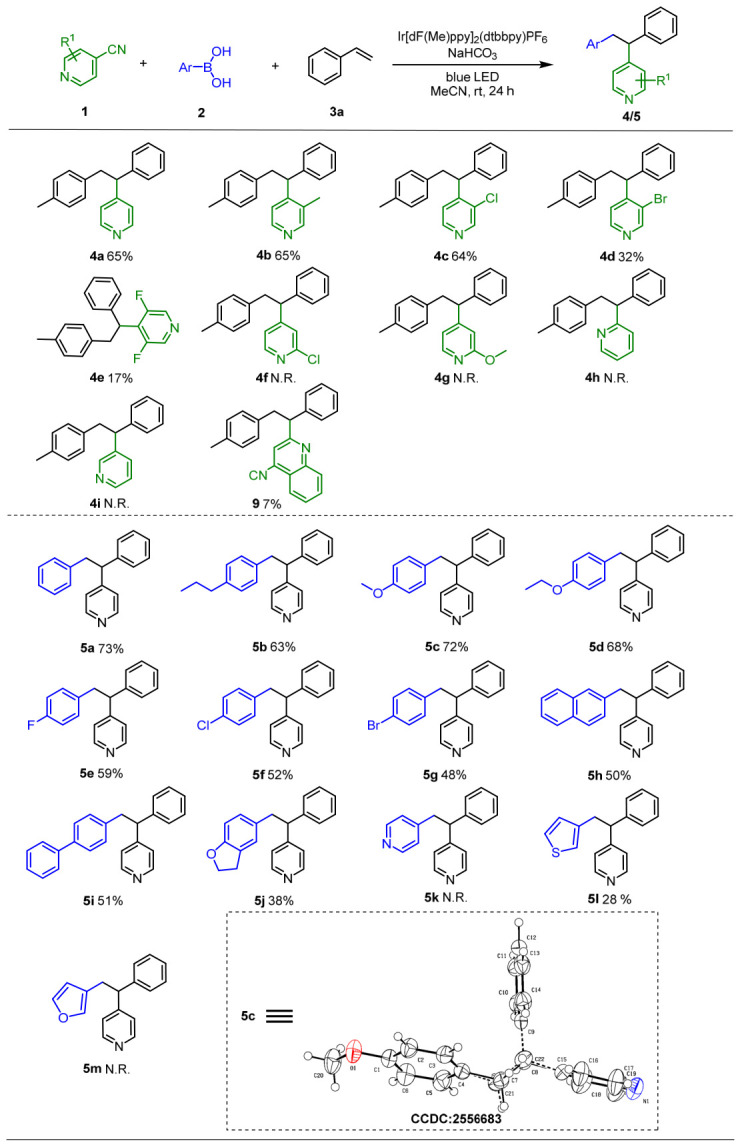
Scope of arylboronic acid and cyanopyridine. Reaction conditions: **1** (0.2 mmol), **2** (9 equiv), **3a** (4 equiv), NaHCO_3_ (2 equiv), Ir[dF(Me)ppy]_2_(dtbbpy)PF_6_ (1.2 mol%), MeCN (1 mL), room temperature, 3 W blue LEDs, under an Ar atmosphere, and for 24 h. Isolated yield. “N.R.” stands for “no reaction”.

Having optimized the reaction parameters, we next investigated the substrate scope of this three-component arylpyridylation reaction. We first examined the reactivity of cyanopyridines under the optimal conditions (Scheme 2). Notably, only 3-substituted 4-cyanopyridines were reactive, whereas their 2 substituted 4 cyanopyridines (**4f**, **4g**), 2-cyanopyridines (**4h**), and 3-cyanopyridines (**4i**) remained unreactive. For 3-substituted 4-cyanopyridines, both electron-donating and electron-withdrawing substituents on the pyridine ring were well tolerated, affording moderate yields (**4a**–**4c**, 64–66%). Nevertheless, bromo-substituted cyanopyridines delivered relatively low yields (**4d**, 32%), which could be attributed to the large atomic radius of bromine. Furthermore, increasing the number of electron-withdrawing groups led to a significant drop in product yields (**4e**, 17%). In addition, when quinoline-4-carbonitrile was used as the substrate, the isolated product was not our target compound. Instead, cyano-retained product **9** via C2-selective functionalization was obtained, with an isolated yield of 7%.

Next, we further explored the substrate scope of aryl boronic acids (Scheme 2). The results showed that both electron-donating (**5a**–**5d**, 68–73%) and electron-withdrawing (**5e**–**5g**, 48–59%) substituents were well tolerated. Notably, aryl boronic acids bearing electron-donating groups afforded slightly higher yields than those with electron-withdrawing substituents. In addition, naphthalene (**5h**, 50%), biphenyl (**5i**, 51%), and benzofuran (**5j**, 38%) were also compatible with this reaction, albeit with relatively low yields. However, the reaction with 3-thiopheneboronic acid (**5l**) gave a low yield, along with two-component byproducts that prevented the isolation of pure product. No reactivity was observed for 3-furanboronic acid (**5m**) and 4-pyridinylboronic acid (**5k**).

**Scheme 3:**
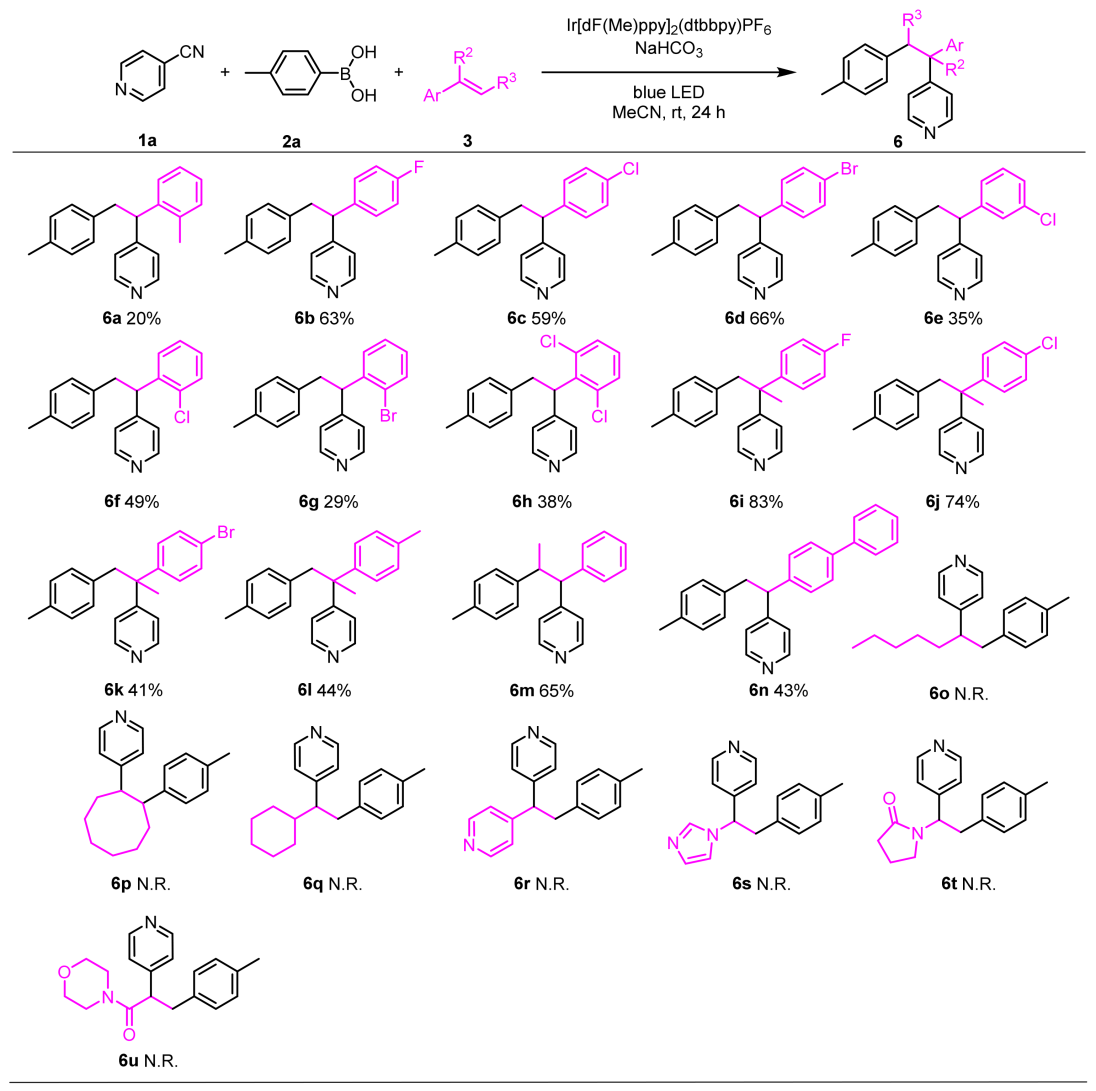
Scope of alkenes. Reaction conditions: **1a** (0.2 mmol), **2a** (9 equiv), **3** (4 equiv), NaHCO_3_ (2 equiv), Ir[dF(Me)ppy]_2_(dtbbpy)PF_6_ (1.2 mol%), MeCN (1 mL), room temperature, 3 W blue LEDs, under an Ar atmosphere, and for 24 h. Isolated yield. “N.R.” stands for “no reaction”.

Subsequently, we investigated the substrate scope of alkenes (Scheme 3). Both electron-donating and electron-withdrawing substituents were compatible with the reaction (**6a**–**6h**, **6n**, 20–66%). However, ortho- and meta-substitution significantly reduced the product yields. In addition, this method is compatible with sterically hindered α-methyl-substituted styrenes, furnishing pyridyl-bearing quaternary carbon centers (**6i**–**6l**, 41–83%). Higher yields were obtained for substrates bearing electron-withdrawing substituents. Furthermore, internal alkenes were also reactive under these conditions (**6m**, 65%). However, alkyl alkenes (**6o**–**6q**), heterocyclic alkenes (**6r**, **6s**), N-vinyl-2-pyrrolidone (**6t**), and 4-acryloylmorpholine (**6u**) showed no reactivity under the standard reaction conditions.

**Scheme 4:**
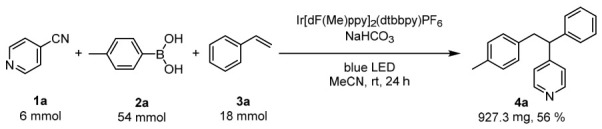
Gram-scale reaction. Reaction conditions: **1a** (6 mmol, 1 equiv), **2a** (9 equiv), **3a** (4 equiv), NaHCO_3_ (2 equiv), Ir[dF(Me)ppy]_2_(dtbbpy)PF_6_ (1.2 mol%), MeCN (30 mL), room temperature, 40 W blue LEDs, under an Ar atmosphere, and for 24 h. Isolated yields.

To further demonstrate the practical applicability of our method, we performed gram-scale reactions under the standard conditions for products **4a** (Scheme 4). The yield of the target compound was 56% (see Supplementary Materials for details).

### 2.3. Plausible Reaction Mechanism

**Scheme 5:**
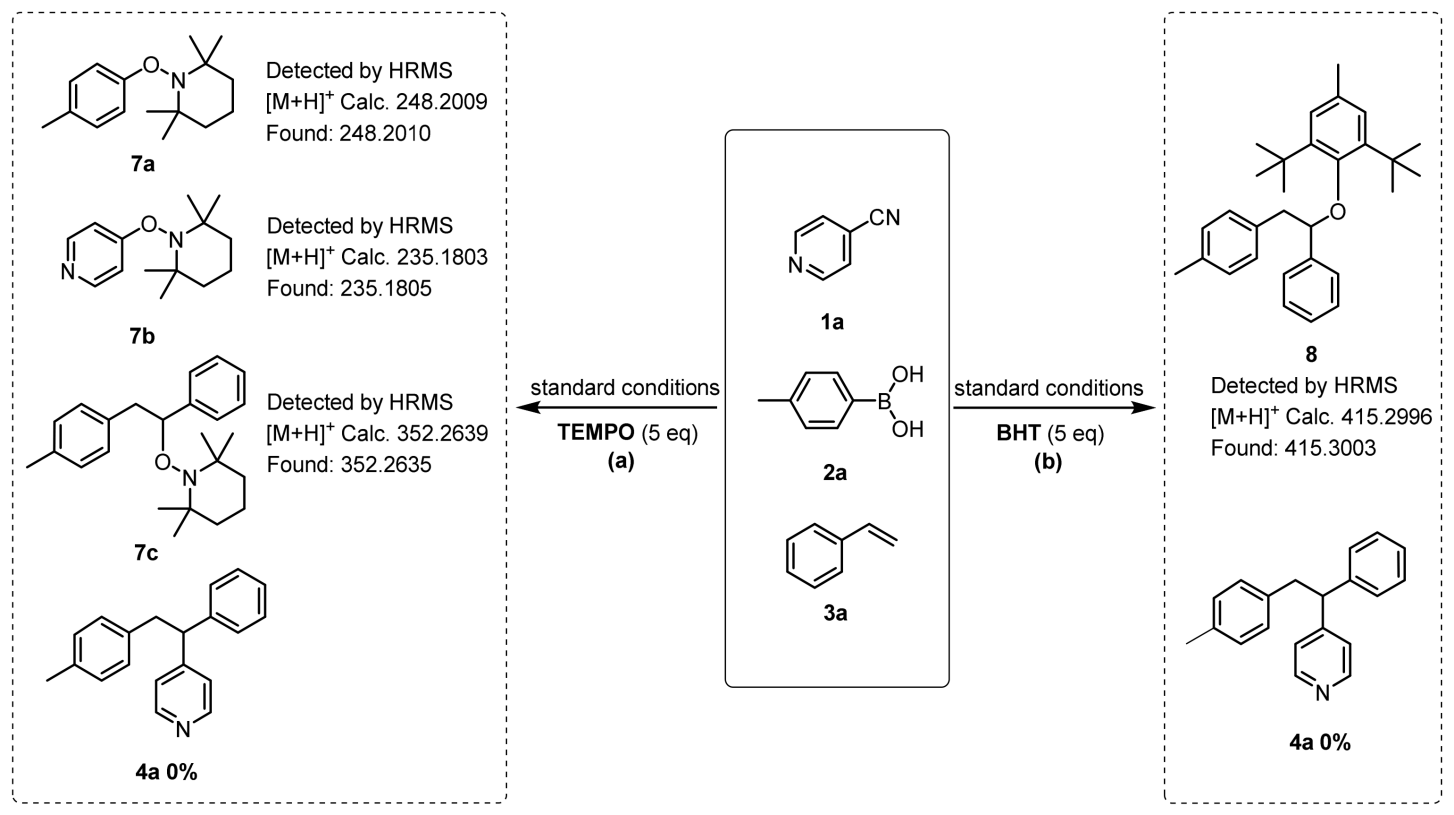
Control experiments. (**a**) TEMPO trapping experiment; (**b**) BHT trapping experiment.

To investigate the mechanism of the reaction, free radical trapping experiments were conducted. When the radical scavenger 2,2,6,6-tetramethylpiperidine (TEMPO) was added to the model reaction, the reaction was completely inhibited and no product **4a** was detected (Scheme 5a; for details, see Supplementary Materials). The reaction was also found to be effectively quenched by the addition of another radical scavenger 2,6-di-tert-butyl-4-methylphenol (BHT) (Scheme 5b; for details, see Supplementary Materials). Furthermore, the corresponding trapped radical adducts **7** and **8** were unambiguously detected by HRMS. These facts strongly suggest that the reactions proceed via a radical pathway.

**Scheme 6:**
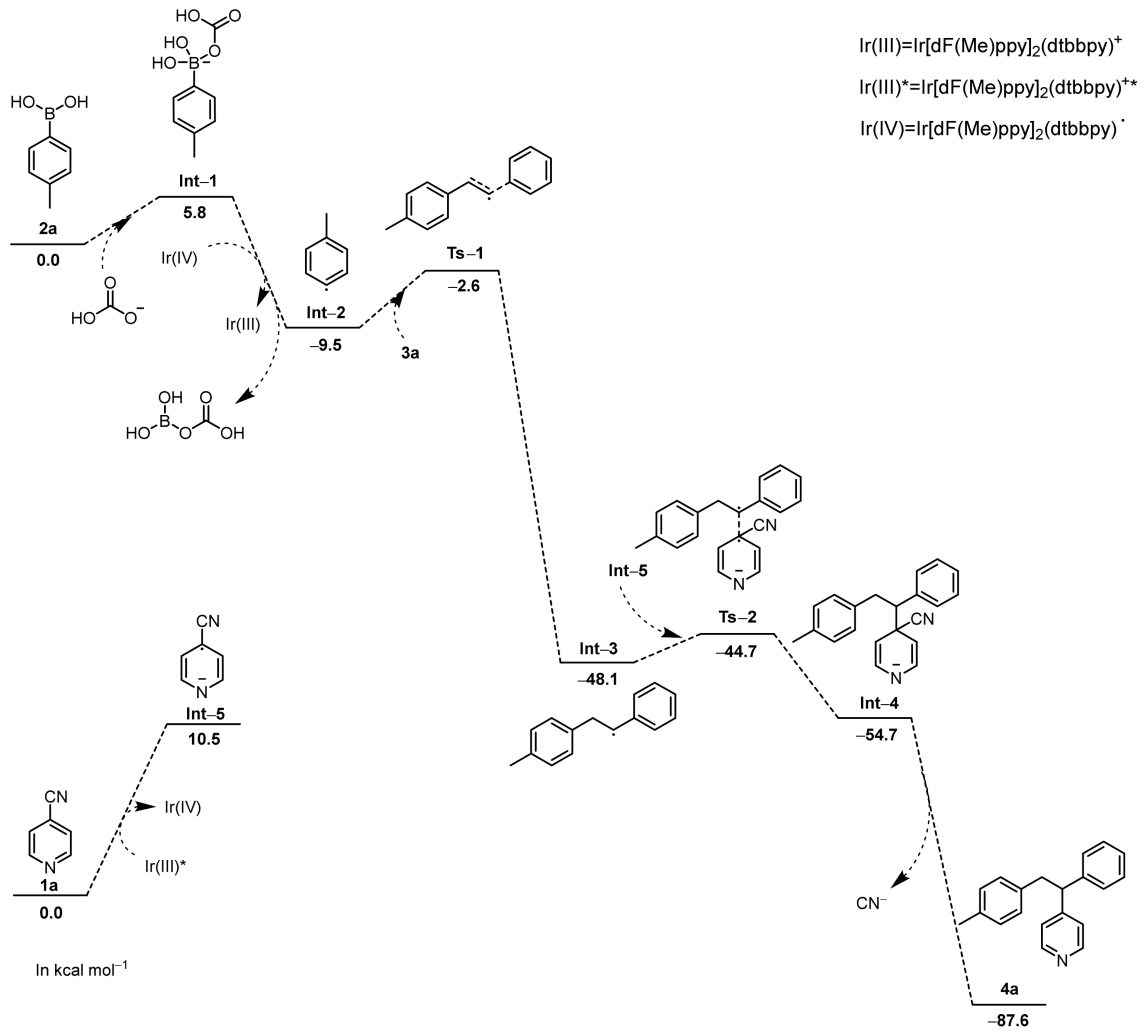
Energy profile of the reaction and proposed reaction pathway. The Gibbs free energies were calculated at the PBE0-D3(BJ)/def2-TZVP level with SMD continuum solvent model for acetonitrile.

To further examine the proposed reaction mechanism, we performed density functional theory (DFT) calculations on the energy profile of the reaction sequence for the reaction of 4-cyanopyridine **1a**, 4-methylphenylboronic acid **2a**, and styrene **3a**, using Gaussian 09 program (Scheme 6). (For computational details, see Supplementary Materials.)

First, 4-cyanopyridine with an onset underwent one-electron reduction by the excited photocatalyst Ir^III^* to give 4-cyanopyridine anion radical **Int-5**. Meanwhile, the bicarbonate ion combined with **2a** to form the intermediate **Int-1**. Subsequently, single-electron transfer (SET) between **Int-1** and Ir^IV^ occurred, Ir^IV^ was reduced to Ir^III^, and **Int-1** was oxidized to the aryl radical **Int-2**. Subsequent radical addition of **Int-2** with styrene **3a** generated radical **Int-3** with a barrier of 6.9 kcal mol^−1^. Radical pair recombination of **Int-3** with **Int-5** gave **Int-4** with a barrier of 3.4 kcal mol^−1^. Finally, spontaneous release of a cyanide anion from **Int-4** yielded the final product **4a**.

**Scheme 7:**
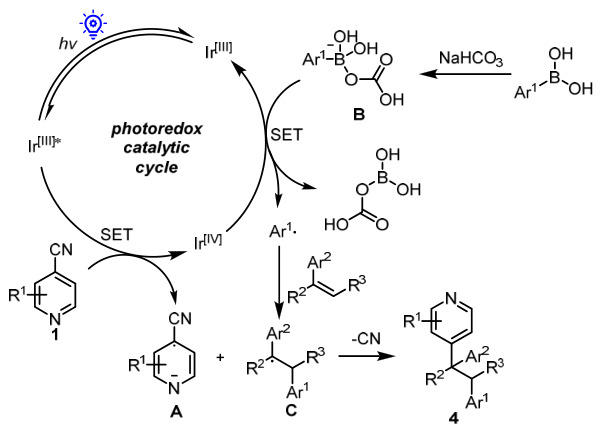
Proposed mechanism.

Based on these experimental and computational results, we suggest the reaction mechanism in Scheme 7. First, SET between the excited state Ir^III^* and cyanopyridine yielded a radical anion intermediate **A**. Meanwhile, boronic acid forms complex **B** under the action of a base. Complex **B** undergoes single-electron transfer with the Ir^IV^, generating an aryl radical, which is captured by the alkene to form intermediate **C**. Subsequently, radical pair recombination of **A** and **C** followed by extrusion of a cyano group gives the final product 4. These computational results provide firm support to the proposed mechanism in Scheme 7.

## 3. Materials and Methods

### 3.1. General Information

Deuterated solvents were purchased from Cambridge Isotope Laboratories. ^1^H NMR spectra were recorded on Bruker AVANCE III 400 (Bruker, Ettlingen, Germany) with 400 MHz frequencies, and ^13^C NMR spectra were recorded on Bruker AVANCE III 400 with 100 MHz frequencies. ^19^F NMR spectra were recorded on a Bruker AVANCE III 400 spectrometer with 376 MHz. Chemical shifts (δ) were reported in ppm relative to the residual solvent signal (CDCl_3_ δ = 7.26 for ^1^H NMR and δ = 77.0 for ^13^C NMR). Chemical shifts (ppm) were recorded with tetramethylsilane (TMS) as the internal reference standard. Multiplicities are given as s (singlet), d (doublet), t (triplet), dd (doublet of doublets), td (triplet of doublets), or m (multiplet). HRMS were obtained using a TOF instrument equipped with an ESI source (Agilent 6224, Agilent Technologies, Inc., Santa Clara, CA, USA). Data collection for crystal structure was performed at room temperature using Mo Kα radiation on a Bruker APEXII diffractometer (Bruker, Ettlingen, Germany).

### 3.2. Experimental Procedure for Compounds ***4***/***5***/***6***

CH_3_CN (1.0 mL) was added to a 4 mL transparent glass bottle equipped with a magnetic stirring bar and a rubber septum. Then Ir[dF(Me)ppy]_2_(dtbbpy)PF_6_ (1.2 mol%), cyanopyridine **1** (0.2 mmol, 1.0 equiv), boronic acid **2** (1.8 mmol, 9.0 equiv), alkene **3** (0.8 mmol, 4.0 equiv), and NaHCO_3_ (0.4 mmol, 2.0 equiv) were added sequentially. The mixture was purged with Ar to create an inert atmosphere. The reaction mixture was irradiated with 3 W blue LEDs (420 nm, 100% intensity) for 24 h at ambient temperature. After the reaction (monitored by TLC), the crude mixture was subjected to column chromatography on silica gel (eluted with a DCM:MeOH ratio of 200:1). The combined eluents were dried over anhydrous MgSO_4_, concentrated under vacuum and purified to afford the desired products **4**/**5**/**6**.

### 3.3. TEMPO Trapping Experiment

CH_3_CN (1.0 mL) was added to a 4 mL transparent glass bottle with magnetic stirring bars and rubber plugs; then, **TEMPO** (1.0 mmol, 5.0 equiv), **1a** (0.2 mmol, 1.0 equiv), **2a** (1.8 mmol, 9.0 equiv), **3a** (0.8 mmol, 4.0 equiv), NaHCO_3_ (0.4 mmol, 2.0 equiv), and Ir[dF(Me)ppy]_2_(dtbbpy)PF_6_ (1.2 mol%) were added to the bottle. The mixture was purged with Ar to create an inert atmosphere. The reaction mixture was irradiated with 3 W blue LEDs (420 nm, 100% intensity) for 24 h at ambient temperature. TLC analysis indicates that no product **4a** was produced, and the TEMPO-trapped product **7** was obtained by HRMS.

### 3.4. BHT Trapping Experiment

CH_3_CN (1.0 mL) was added to a 4 mL transparent glass bottle with magnetic stirring bars and rubber plugs; then, **BHT** (1.0 mmol, 5.0 equiv), **1a** (0.2 mmol, 1.0 equiv), **2a** (1.8 mmol, 9.0 equiv), **3a** (0.8 mmol, 4.0 equiv), NaHCO_3_ (0.4 mmol, 2.0 equiv), and Ir[dF(Me)ppy]_2_(dtbbpy)PF_6_ (1.2 mol%) were added to the bottle. The mixture was purged with Ar to create an inert atmosphere. The reaction mixture was irradiated with 3 W blue LEDs (420 nm, 100% intensity) for 24 h at ambient temperature. After the reaction (monitored by TLC), TLC analysis indicates that no product **4a** was produced, and the BHT-trapped product **8** was obtained by HRMS.

### 3.5. Characterization Data for All Products ***4a***–***4e***, ***5a***–***5j***, and ***6a***–***6n***

**4-(1-phenyl-2-(p-tolyl)ethyl)pyridine (4a)**: Purification by column chromatography (silica gel, DCM/MeOH = 200:1) afforded **4a** (65%, 35.5 mg) as a colorless liquid. ^1^H NMR (400 MHz, Chloroform-*d*) δ 8.44 (d, *J* = 5.7 Hz, 2H), 7.34–7.26 (m, 2H), 7.23–7.17 (m, 3H), 7.14–7.06 (m, 2H), 7.03–6.81 (m, 4H), 4.19 (t, *J* = 7.8 Hz, 1H), 3.44–3.17 (m, 2H), 2.26 (s, 3H). ^13^C NMR (100 MHz, Chloroform-*d*) δ 153.3, 149.7, 142.8, 136.1, 135.7, 128.9, 128.8, 128.6, 128.0, 126.8, 123.5, 52.6, 40.9, 21.0. HRMS (ESI-TOF) for C_20_H_20_N ([M + H]^+^): calcd. 274.1591; found: 274.1590.

**3-methyl-4-(1-phenyl-2-(p-tolyl)ethyl)pyridine (4b)**: Purification by column chromatography (silica gel, DCM/MeOH = 200:1) afforded **4b** (65%, 37.3 mg) as a colorless liquid. ^1^H NMR (400 MHz, Chloroform-*d*) δ 8.41 (d, *J* = 5.1 Hz, 1H), 8.27 (s, 1H), 7.30 (d, *J* = 5.1 Hz, 1H), 7.25–7.22 (m, 2H), 7.21–7.16 (m, 1H), 7.13–7.07 (m, 2H), 7.01–6.95 (m, 2H), 6.88–6.83 (m, 2H), 4.37 (t, *J* = 7.7 Hz, 1H), 3.37–3.20 (m, 2H), 2.27 (s, 3H), 2.07 (s, 3H). ^13^C NMR (100 MHz, Chloroform-*d*) δ 151.5, 150.7, 147.4, 142.2, 136.1, 135.7, 132.0, 128.9, 128.8, 128.5, 128.2, 126.6, 121.9, 48.3, 41.2, 21.0, 16.5. HRMS (ESI-TOF) for C_21_H_22_N ([M + H]^+^): calcd. 288.1747; found: 288.1744.

**3-chloro-4-(1-phenyl-2-(p-tolyl)ethyl)pyridine (4c)**: Purification by column chromatography (silica gel, DCM/MeOH = 200:1) afforded **4c** (64%, 39.3 mg) as a light yellow solid. ^1^H NMR (400 MHz, Chloroform-*d*) δ 8.47 (s, 1H), 8.40 (d, *J* = 5.0 Hz, 1H), 7.32–7.24 (m, 3H), 7.23–7.17 (m, 3H), 7.01–6.90 (m, 4H), 4.76 (t, *J* = 7.9 Hz, 1H), 3.40–3.23 (m, 2H), 2.25 (s, 3H). ^13^C NMR (100 MHz, Chloroform-*d*) δ 150.5, 149.6, 147.7, 141.0, 135.8, 135.6, 132.2, 129.0, 128.7, 128.5, 128.3, 126.9, 123.3, 48.1, 40.2, 21.0. HRMS (ESI-TOF) for C_20_H_19_ClN ([M + H]^+^): calcd. 308.1201; found: 308.1209.

**3-bromo-4-(1-phenyl-2-(p-tolyl)ethyl)pyridine (4d)**: Purification by column chromatography (silica gel, DCM/MeOH = 200:1) afforded **4d** (32%, 22.5 mg) as a white solid (84–87 °C). ^1^H NMR (400 MHz, Chloroform-*d*) δ 8.62 (s, 1H), 8.43 (d, *J* = 5.1 Hz, 1H), 7.33–7.26 (m, 3H), 7.25–7.20 (m, 3H), 7.02–6.94 (m, 4H), 4.76 (t, *J* = 7.9 Hz, 1H), 3.36–3.25 (m, 2H), 2.27 (s, 3H). ^13^C NMR (100 MHz, Chloroform-*d*) δ 152.2, 152.2, 148.2, 141.0, 135.9, 135.5, 129.0, 128.7, 128.6, 128.4, 126.9, 123.9, 50.5, 40.5, 21.0. HRMS (ESI-TOF) for C_20_H_19_BrN ([M + H]^+^): calcd. 352.0696; found: 352.0673.

**3,5-difluoro-4-(1-phenyl-2-(p-tolyl)ethyl)pyridine (4e)**: Purification by column chromatography (silica gel, DCM/MeOH = 200:1) afforded **4e** (17%, 10.5 mg) as a white solid (103–105 °C). ^1^H NMR (400 MHz, Chloroform-*d*) δ 8.17 (s, 2H), 7.39–7.35 (m, 2H), 7.31 (t, *J* = 7.5 Hz, 2H), 7.25–7.21 (m, 1H), 6.99 (s, 4H), 4.73 (t, *J* = 8.4 Hz, 1H), 3.49 (d, *J* = 8.3 Hz, 2H), 2.25 (s, 3H). ^13^C NMR (100 MHz, Chloroform-*d*) δ 157.6 (d, *J* = 255.0 Hz), 140.7, 136.0, 135.8, 134.5, 134.2, 129.1, 128.7, 128.4, 127.8, 127.2, 42.6, 37.9, 21.0. ^19^F NMR (377 MHz, Chloroform-*d*) δ −127.79. HRMS (ESI-TOF) for C_20_H_18_F_2_N ([M + H]^+^): calcd. 310.1402; found: 310.1417.

**4-(1,2-diphenylethyl)pyridine (5a)**: Purification by column chromatography (silica gel, DCM/MeOH = 200:1) afforded **5a** (73%, 37.8 mg) as a colorless liquid. ^1^H NMR (400 MHz, Chloroform-*d*) δ 8.44 (d, *J* = 6.1 Hz, 2H), 7.30–7.25 (m, 2H), 7.23–7.07 (m, 8H), 7.00–6.94 (m, 2H), 4.21 (t, *J* = 7.8 Hz, 1H), 3.41–3.26 (m, 2H). ^13^C NMR (100 MHz, Chloroform-*d*) δ 149.2, 142.5, 139.1, 129.0, 128.7, 128.3, 128.0, 126.9, 126.3, 123.6, 52.6, 41.3. HRMS (ESI-TOF) for C_19_H_18_N ([M + H]^+^): calcd. 260.1434; found: 260.1429.

**4-(1-phenyl-2-(4-propylphenyl)ethyl)pyridine (5b)**: Purification by column chromatography (silica gel, DCM/MeOH = 200:1) afforded **5b** (63%, 37.9 mg) as a brown solid (62–64 °C). ^1^H NMR (400 MHz, Chloroform-*d*) δ 8.43 (d, *J* = 5.8 Hz, 2H), 7.29–7.24 (m, 2H), 7.22–7.16 (m, 3H), 7.11–7.07 (m, 2H), 7.00–6.96 (m, 2H), 6.91–6.86 (m, 2H), 4.19 (t, *J* = 7.8 Hz, 1H), 3.38–3.23 (m, 2H), 2.49 (t, 2H), 1.64–1.51 (m, 2H), 0.89 (t, *J* = 7.3 Hz, 3H). ^13^C NMR (100 MHz, Chloroform-*d*) δ 153.4, 149.7, 142.8, 140.6, 136.4, 128.8, 128.6, 128.3, 128.0, 126.8, 123.5, 52.6, 41.0, 37.6, 24.5, 13.8. HRMS (ESI-TOF) for C_22_H_24_N ([M + H]^+^): calcd. 302.1904; found: 302.1911.

**4-(2-(4-methoxyphenyl)-1-phenylethyl)pyridine (5c)**: Purification by column chromatography (silica gel, DCM/MeOH = 200:1) afforded **5c** (72%, 41.6 mg) as a brown solid (81–82 °C). ^1^H NMR (400 MHz, Chloroform-*d*) δ 8.44 (d, *J* = 5.3 Hz, 2H), 7.31–7.23 (m, 3H), 7.23–7.15 (m, 3H), 7.09 (d, *J* = 5.8 Hz, 2H), 6.92–6.85 (m, 2H), 6.74–6.67 (m, 2H), 4.15 (t, *J* = 7.8 Hz, 1H), 3.72 (s, 3H), 3.36–3.20 (m, 2H). ^13^C NMR (100 MHz, Chloroform-*d*) δ 158.0, 153.6, 149.5, 142.7, 131.3, 129.9, 128.7, 128.1, 126.8, 123.6, 113.7, 55.2, 52.8, 40.5. HRMS (ESI-TOF) for C_20_H_20_NO ([M + H]^+^): calcd. 290.1539; found: 290.1549.

**4-(2-(4-ethoxyphenyl)-1-phenylethyl)pyridine (5d)**: Purification by column chromatography (silica gel, DCM/MeOH = 200:1) afforded **5d** (68%, 41.2 mg) as a yellow oily liquid. ^1^H NMR (400 MHz, Chloroform-*d*) δ 8.43 (d, *J* = 4.8 Hz, 2H), 7.30–7.24 (m, 2H), 7.22–7.16 (m, 3H), 7.08 (d, *J* = 5.8 Hz, 2H), 6.89–6.85 (m, 2H), 6.73–6.67 (m, 2H), 4.15 (t, *J* = 7.8 Hz, 1H), 3.94 (q, *J* = 7.0 Hz, 2H), 3.34–3.20 (m, 2H), 1.36 (t, *J* = 7.0 Hz, 3H). ^13^C NMR (100 MHz, Chloroform-d) δ 157.4, 153.6, 149.5, 142.7, 131.2, 129.9, 128.6, 128.1, 126.8, 123.6, 114.3, 63.3, 52.9, 40.6, 14.9. HRMS (ESI-TOF) for C_21_H_22_NO ([M + H]^+^): calcd. 304.1696; found: 304.1722.

**4-(2-(4-fluorophenyl)-1-phenylethyl)pyridine (5e)**: Purification by column chromatography (silica gel, DCM/MeOH = 200:1) afforded **5e** (59%, 32.7 mg) as a light yellow liquid. ^1^H NMR (400 MHz, Chloroform-*d*) δ 8.49–8.43 (m, 2H), 7.33–7.24 (m, 3H), 7.25–7.17 (m, 2H), 7.20–7.14 (m, 2H), 7.13–7.07 (m, 2H), 6.97–6.81 (m, 4H), 4.14 (t, *J* = 7.9 Hz, 1H), 3.39–3.24 (m, 2H). ^13^C NMR (100 MHz, Chloroform-*d*) δ 161.4 (d, *J* = 244.3 Hz), 153.1, 149.7, 142.3, 134.9 (d, *J* = 3.1 Hz), 130.4 (d, *J* = 7.8 Hz), 128.7, 128.0, 126.9, 123.4, 115.1 (d, *J* = 21.3 Hz), 52.7, 40.5. ^19^F NMR (377 MHz, Chloroform-*d*) δ −116.73. HRMS (ESI-TOF) for C_19_H_17_FN ([M + H]^+^): calcd. 278.1340; found: 278.1337.

**4-(2-(4-chlorophenyl)-1-phenylethyl)pyridine (5f)**: Purification by column chromatography (silica gel, DCM/MeOH = 200:1) afforded **5f** (52%, 30.5 mg) as a colorless liquid. ^1^H NMR (400 MHz, Chloroform-*d*) δ 8.47 (d, *J* = 5.9 Hz, 2H), 7.33–7.27 (m, 2H), 7.25–7.19 (m, 1H), 7.18–7.10 (m, 6H), 6.94–6.88 (m, 2H), 4.15 (t, *J* = 7.9 Hz, 1H), 3.39–3.24 (m, 2H). ^13^C NMR (100 MHz, Chloroform-*d*) δ 153.2, 149.5, 142.1, 137.6, 130.3, 128.7, 128.4, 128.0, 127.0, 123.4, 52.5, 40.7. HRMS (ESI-TOF) for C_19_H_17_ClN ([M + H]^+^): calcd. 294.1044; found: 294.1045.

**4-(2-(4-bromophenyl)-1-phenylethyl)pyridine (5g)**: Purification by column chromatography (silica gel, DCM/MeOH = 200:1) afforded **5g** (48%, 32.4 mg) as a light yellow liquid. ^1^H NMR (400 MHz, Chloroform-*d*) δ 8.47 (d, *J* = 5.2 Hz, 2H), 7.31–7.28 (m, 3H), 7.26 (d, *J* = 3.1 Hz, 1H), 7.24–7.19 (m, 1H), 7.19–7.14 (m, 2H), 7.10 (d, *J* = 5.9 Hz, 2H), 6.88–6.83 (m, 2H), 4.15 (t, *J* = 7.9 Hz, 1H), 3.37–3.23 (m, 2H). ^13^C NMR (100 MHz, Chloroform-*d*) δ 153.0, 149.7, 142.1, 138.2, 131.4, 130.7, 128.7, 127.9, 127.0, 123.4, 120.2, 52.4, 40.7, 29.7. HRMS (ESI-TOF) for C_19_H_17_BrN ([M + H]^+^): calcd. 338.0539; found: 338.0536.

**4-(2-(naphthalen-2-yl)-1-phenylethyl)pyridine (5h)**: Purification by column chromatography (silica gel, DCM/MeOH = 200:1) afforded **5h** (50%, 30.9 mg) as a light yellow liquid. ^1^H NMR (400 MHz, Chloroform-*d*) δ 8.45 (d, *J* = 4.8 Hz, 2H), 7.79–7.74 (m, 1H), 7.70–7.64 (m, 2H), 7.45–7.39 (m, 3H), 7.32–7.26 (m, 2H), 7.25–7.19 (m, 3H), 7.17–7.11 (m, 3H), 4.33 (t, *J* = 7.8 Hz, 1H), 3.59–3.44 (m, 2H). ^13^C NMR (100 MHz, Chloroform-*d*) δ 153.6, 149.4, 142.5, 136.7, 133.4, 132.1, 128.7, 128.0, 127.9, 127.6, 127.5, 127.3, 126.9, 126.0, 125.4, 123.6, 52.5, 41.5. HRMS (ESI-TOF) for C_23_H_20_N ([M + H]^+^): calcd. 310.1590; found: 310.1421.

**4-(2-([1,1′-biphenyl]-4-yl)-1-phenylethyl)pyridine (5i)**: Purification by column chromatography (silica gel, DCM/MeOH = 200:1) afforded **5i** (51%, 34.2 mg) as a light yellow solid (137–139 °C). ^1^H NMR (400 MHz, Chloroform-*d*) δ 8.45 (d, *J* = 5.6 Hz, 2H), 7.53 (d, *J* = 7.5 Hz, 2H), 7.43–7.37 (m, 4H), 7.33–7.26 (m, 3H), 7.23–7.18 (m, 3H), 7.11 (d, *J* = 5.8 Hz, 2H), 7.07–7.02 (m, 2H), 4.23 (t, *J* = 7.8 Hz, 1H), 3.44–3.31 (m, 2H). ^13^C NMR (100 MHz, Chloroform-*d*) δ 153.2, 149.7, 142.5, 140.7, 139.0, 138.3, 129.4, 128.7, 128.6, 128.0, 127.1, 126.9, 126.9, 126.8, 123.4, 52.4, 40.9. HRMS (ESI-TOF) for C_25_H_21_N ([M + H]^+^): calcd. 336.1747; found: 336.1741.

**4-(2-(2,3-dihydrobenzofuran-5-yl)-1-phenylethyl)pyridine (5j)**: Purification by column chromatography (silica gel, DCM/MeOH = 200:1) afforded **5j** (38%, 22.9 mg) as a light yellow solid (84–86 °C). ^1^H NMR (400 MHz, Chloroform-*d*) δ 8.45 (d, *J* = 4.7 Hz, 2H), 7.30–7.26 (m, 2H), 7.23–7.17 (m, 3H), 7.09 (d, *J* = 5.7 Hz, 2H), 6.79 (s, 1H), 6.74–6.68 (m, 1H), 6.61–6.56 (m, 1H), 4.50 (t, *J* = 8.7 Hz, 2H), 4.14 (t, *J* = 7.8 Hz, 1H), 3.26 (tt, *J* = 13.7, 7.4 Hz, 2H), 3.08 (t, *J* = 8.7 Hz, 2H). ^13^C NMR (100 MHz, Chloroform-*d*) δ 158.5, 153.4, 149.6, 142.7, 131.1, 128.6, 128.4, 128.0, 126.9, 126.8, 125.4, 123.5, 108.8, 71.1, 53.0, 40.8, 29.7. HRMS (ESI-TOF) for C_21_H_20_NO ([M + H]^+^): calcd. 302.1539; found: 302.1529.

**4-(1-(o-tolyl)-2-(p-tolyl)ethyl)pyridine (6a)**: Purification by column chromatography (silica gel, DCM/MeOH = 200:1) afforded **6a** 20%, 11.5 mg) as a colorless liquid. ^1^H NMR (400 MHz, Chloroform-*d*) δ 8.41 (s, 2H), 7.41–7.33 (m, 1H), 7.26–7.07 (m, 4H), 7.03–6.94 (m, 4H), 6.89–6.81 (m, 2H), 4.36 (dd, *J* = 8.6, 6.7 Hz, 1H), 3.37–3.18 (m, 2H), 2.28 (s, 3H), 2.11 (s, 3H). ^13^C NMR (100 MHz, Chloroform-*d*) δ 153.1, 149.5, 140.9, 136.3, 136.2, 135.8, 130.6, 129.0, 128.9, 126.9, 126.7, 126.3, 123.8, 48.3, 41.3, 21.0, 19.8. HRMS (ESI-TOF) for C_21_H_22_N ([M + H]^+^): calcd. 288.1747; found: 288.1742.

**4-(1-(4-fluorophenyl)-2-(p-tolyl)ethyl)pyridine (6b)**: Purification by column chromatography (silica gel, DCM/MeOH = 200:1) afforded **6b** (63%, 36.7 mg) as a colorless liquid. ^1^H NMR (400 MHz, Chloroform-*d*) δ 8.46 (d, *J* = 5.1 Hz, 2H), 7.15–7.06 (m, 4H), 7.00–6.93 (m, 4H), 6.89–6.84 (m, 2H), 4.17 (t, *J* = 7.8 Hz, 1H), 3.27 (d, *J* = 7.8 Hz, 2H), 2.27 (s, 3H). ^13^C NMR (100 MHz, Chloroform-*d*) δ 161.61 (d, *J* = 245.2 Hz), 153.3, 149.7, 138.4 (d, *J* = 3.2 Hz), 135.84, 135.81, 129.5 (d, *J* = 7.8 Hz), 128.90 (d, *J* = 21.3 Hz), 123.4, 115.5, 115.3, 51.8, 41.0, 21.0. ^19^F NMR (377 MHz, Chloroform-d) δ −115.95. HRMS (ESI-TOF) for C_20_H_19_FN ([M + H]^+^): calcd. 292.1496; found: 292.1490.

**4-(1-(4-chlorophenyl)-2-(p-tolyl)ethyl)pyridine (6c)**: Purification by column chromatography (silica gel, DCM/MeOH = 200:1) afforded **6c** (59%, 36.2 mg) as a light yellow solid (109–112 °C). ^1^H NMR (400 MHz, Chloroform-*d*) δ 8.46 (d, *J* = 4.6 Hz, 2H), 7.26–7.22 (m, 2H), 7.13–7.06 (m, 4H), 7.03–6.96 (m, 2H), 6.90–6.84 (m, 2H), 4.16 (t, *J* = 7.8 Hz, 1H), 3.27 (d, *J* = 7.8 Hz, 2H), 2.27 (s, 3H). ^13^C NMR (100 MHz, Chloroform-*d*) δ 153.0, 149.7, 141.1, 135.9, 135.6, 132.6, 129.4, 129.0, 128.8, 128.7, 123.4, 51.9, 40.8, 21.0. HRMS (ESI-TOF) for C_20_H_19_ClN ([M + H]^+^): calcd. 308.1201; found: 308.1208.

**4-(1-(4-bromophenyl)-2-(p-tolyl)ethyl)pyridine (6d)**: Purification by column chromatography (silica gel, DCM/MeOH = 200:1) afforded **6d** (66%, 46.3 mg) as a yellow oily liquid. ^1^H NMR (400 MHz, Chloroform-*d*) δ 8.57–8.36 (m, 2H), 7.47–7.32 (m, 2H), 7.10–7.06 (m, 2H), 7.06–7.04 (m, 1H), 7.04–7.02 (m, 1H), 7.02–6.84 (m, 4H), 4.15 (t, *J* = 7.8 Hz, 1H), 3.27 (d, *J* = 7.8 Hz, 2H), 2.27 (s, 3H). ^13^C NMR (100 MHz, Chloroform-*d*) δ 152.7, 149.9, 141.7, 135.9, 135.7, 131.7, 129.8, 129.1, 128.8, 123.3, 120.7, 52.0, 40.8, 21.0. HRMS (ESI-TOF) for C_20_H_19_BrN ([M + H]^+^): calcd. 352.0696; found: 352.0698.

**4-(1-(3-chlorophenyl)-2-(p-tolyl)ethyl)pyridine (6e)**: Purification by column chromatography (silica gel, DCM/MeOH = 200:1) afforded **6e** (35%, 21.5 mg) as a yellow oily liquid. ^1^H NMR (400 MHz, Chloroform-*d*) δ 8.54–8.40 (m, 2H), 7.21–7.04 (m, 6H), 7.01–6.94 (m, 2H), 6.90–6.84 (m, 2H), 4.16 (t, *J* = 7.8 Hz, 1H), 3.38–3.18 (m, 2H), 2.27 (s, 3H). ^13^C NMR (100 MHz, Chloroform-*d*) δ 152.5, 149.8, 144.7, 135.9, 135.6, 134.4, 129.8, 129.1, 128.8, 128.1, 127.0, 126.3, 123.4, 52.3, 40.7, 21.0. HRMS (ESI-TOF) for C_20_H_19_ClN ([M + H]^+^): calcd. 308.1201; found: 308.1202.

**4-(1-(2-chlorophenyl)-2-(p-tolyl)ethyl)pyridine (6f)**: Purification by column chromatography (silica gel, DCM/MeOH = 200:1) afforded **6f** (49%, 30.1 mg) as a yellow oily liquid. ^1^H NMR (400 MHz, Chloroform-*d*) δ 8.46 (s, 2H), 7.40–7.32 (m, 2H), 7.29–7.26 (m, 1H), 7.17 (td, J = 7.6, 1.7 Hz, 1H), 7.12–7.05 (m, 2H), 7.01–6.92 (m, 4H), 4.80–4.74 (m, 1H), 3.35–3.22 (m, 2H), 2.27 (s, 3H). ^13^C NMR (100 MHz, Chloroform-d) δ 151.8, 149.7, 140.2, 135.9, 135.6, 134.3, 129.9, 129.0, 128.8, 128.6, 128.1, 127.1, 48.0, 40.3, 21.0. HRMS (ESI-TOF) for C_20_H_19_ClN ([M + H]^+^): calcd. 308.1201; found: 308.1209.

**4-(1-(2-bromophenyl)-2-(p-tolyl)ethyl)pyridine (6g)**: Purification by column chromatography (silica gel, DCM/MeOH = 200:1) afforded **6g** (29%, 20.4 mg) as a yellow oily liquid. ^1^H NMR (400 MHz, Chloroform-*d*) δ 8.45 (s, 2H), 7.55–7.51 (m, 1H), 7.38–7.34 (m, 1H), 7.33–7.27 (m, 1H), 7.11–7.07 (m, 3H), 7.02–6.98 (m, 2H), 6.97–6.93 (m, 2H), 4.78 (dd, J = 8.8, 6.9 Hz, 1H), 3.38–3.17 (m, 2H), 2.27 (s, 3H). ^13^C NMR (100 MHz, Chloroform-d) δ 151.7, 149.7, 141.8, 135.9, 135.6, 133.3, 129.0, 129.0, 128.9, 128.8, 128.4, 127.7, 125.3, 50.5, 40.6, 21.0. HRMS (ESI-TOF) for C_20_H_19_BrN ([M + H]^+^): calcd. 352.0696; found: 352.0704.

**4-(1-(2,6-dichlorophenyl)-2-(p-tolyl)ethyl)pyridine (6h)**: Purification by column chromatography (silica gel, DCM/MeOH = 200:1) afforded **6h** (38%, 25.9 mg) as a light yellow solid (119–121 °C). ^1^H NMR (400 MHz, Chloroform-*d*) δ 8.49 (d, *J* = 4.8 Hz, 2H), 7.24–7.16 (m, 4H), 7.07 (t, *J* = 7.9 Hz, 1H), 7.02–6.94 (m, 4H), 5.35 (t, *J* = 8.0 Hz, 1H), 3.68–3.58 (m, 2H), 2.26 (s, 3H). ^13^C NMR (100 MHz, Chloroform-*d*) δ 151.2, 149.3, 137.7, 135.8, 135.5, 128.9, 128.7, 128.7, 123.0, 46.4, 35.0, 21.0. HRMS (ESI-TOF) for C_20_H_19_Cl_2_N ([M + H]^+^): calcd. 342.2630; found: 342.0783.

**4-(2-(4-fluorophenyl)-1-(p-tolyl)propan-2-yl)pyridine (6i)**: Purification by column chromatography (silica gel, DCM/MeOH = 200:1) afforded **6i** (83%, 50.7 mg) as a white solid (103–105 °C). ^1^H NMR (400 MHz, Chloroform-*d*) δ 8.48 (d, *J* = 5.1 Hz, 2H), 7.11–7.04 (m, 4H), 7.00–6.92 (m, 2H), 6.91–6.86 (m, 2H), 6.53–6.45 (m, 2H), 3.33 (s, 2H), 2.25 (s, 3H), 1.52 (s, 3H). ^13^C NMR (100 MHz, Chloroform-*d*) δ 161.3 (d, *J* = 245.6 Hz), 158.5, 149.5, 143.2 (d, *J* = 3.2 Hz), 135.9, 133.6, 130.5, 129.3 (d, *J* = 7.7 Hz), 128.3, 123.0, 114.87 (d, *J* = 21.1 Hz), 46.8, 46.7, 26.5, 21.0. ^19^F NMR (377 MHz, Chloroform-*d*) δ −116.59. HRMS (ESI-TOF) for C_21_H_21_FN ([M + H]^+^): calcd. 306.1653; found: 306.1663.

**4-(2-(4-chlorophenyl)-1-(p-tolyl)propan-2-yl)pyridine (6j)**: Purification by column chromatography (silica gel, DCM/MeOH = 200:1) afforded **6j** (74%, 47.5 mg) as a colorless liquid. ^1^H NMR (400 MHz, Chloroform-*d*) δ 8.53–8.42 (m, 2H), 7.25–7.21 (m, 2H), 7.07–7.03 (m, 4H), 6.92–6.87 (m, 2H), 6.52–6.46 (m, 2H), 3.33 (s, 2H), 2.25 (s, 3H), 1.52 (s, 3H). ^13^C NMR (100 MHz, Chloroform-*d*) δ 158.1, 149.5, 146.0, 136.0, 133.4, 132.2, 130.5, 129.2, 128.4, 128.2, 123.0, 46.9, 46.5, 26.3, 21.0. HRMS (ESI-TOF) for C_21_H_21_ClN ([M + H]^+^): calcd. 322.1358; found: 322.1328.

**4-(2-(4-bromophenyl)-1-(p-tolyl)propan-2-yl)pyridine (6k)**: Purification by column chromatography (silica gel, DCM/MeOH = 200:1) afforded **6k** (41%, 29.9 mg) as a colorless liquid. ^1^H NMR (400 MHz, Chloroform-*d*) δ 8.54 (s, 2H), 7.43–7.34 (m, 2H), 7.09 (s, 2H), 7.03–6.96 (m, 2H), 6.92–6.87 (m, 2H), 6.55–6.44 (m, 2H), 3.33 (s, 2H), 2.26 (s, 3H), 1.52 (s, 3H). ^13^C NMR (100 MHz, Chloroform-*d*) δ 146.6, 136.0, 133.4, 131.2, 130.5, 129.6, 128.4, 120.4, 47.0, 46.4, 26.2, 21.1. HRMS (ESI-TOF) for C_21_H_21_BrN ([M + H]^+^): calcd. 366.0852; found: 366.0854.

**4-(1,2-di-p-tolylpropan-2-yl)pyridine (6l)**: Purification by column chromatography (silica gel, DCM/MeOH = 200:1) afforded **6l** (44%, 26.5 mg) as a colorless liquid. ^1^H NMR (400 MHz, Chloroform-*d*) δ 8.51–8.34 (m, 2H), 7.14–6.99 (m, 6H), 6.92–6.82 (m, 2H), 6.56–6.45 (m, 2H), 3.42–3.26 (m, 2H), 2.33 (s, 3H), 2.25 (s, 3H), 1.52 (s, 3H). ^13^C NMR (100 MHz, Chloroform-d) δ 158.7, 149.3, 144.7, 135.9, 135.8, 134.0, 130.6, 128.8, 128.2, 127.5, 123.1, 46.8, 46.6, 26.3, 21.1, 21.0. HRMS (ESI-TOF) for C_22_H_24_N ([M + H]^+^): calcd. 302.1904; found: 302.1897.

**4-(1-phenyl-2-(p-tolyl)propyl)pyridine (6m)**: Purification by column chromatography (silica gel, DCM/MeOH = 200:1) afforded **6m** (65%, 37.4 mg) as a light yellow solid (85–87 °C). ^1^H NMR (400 MHz, Chloroform-*d*) δ 8.52 (d, *J* = 4.7 Hz, 2H), 7.33 (d, *J* = 4.8 Hz, 2H), 7.13–7.07 (m, 4H), 7.04–6.93 (m, 5H), 4.04 (d, *J* = 11.3 Hz, 1H), 3.61–3.50 (m, 1H), 2.22 (s, 3H), 1.21–1.16 (m, 3H). ^13^C NMR (100 MHz, Chloroform-*d*) δ 153.6, 149.6, 142.1, 141.7, 135.5, 128.9, 128.3, 128.2, 127.4, 126.3, 123.8, 58.8, 43.2, 22.0, 21.0. HRMS (ESI-TOF) for C_21_H_22_N ([M + H]^+^): calcd. 288.1747; found: 288.1750.

**4-(1-([1,1′-biphenyl]-4-yl)-2-(p-tolyl)ethyl)pyridine (6n)**: Purification by column chromatography (silica gel, DCM/MeOH = 200:1) afforded **6n** (43%, 30.0 mg) as a colorless liquid. ^1^H NMR (400 MHz, Chloroform-*d*) δ 8.47 (d, *J* = 5.8 Hz, 2H), 7.57–7.55 (m, 2H), 7.53–7.50 (m, 2H), 7.42 (t, *J* = 7.5 Hz, 2H), 7.35–7.30 (m, 1H), 7.28–7.26 (m, 2H), 7.13 (d, *J* = 6.1 Hz, 2H), 7.02–6.90 (m, 4H), 4.23 (t, *J* = 7.8 Hz, 1H), 3.42–3.27 (m, 2H), 2.27 (s, 3H). ^13^C NMR (100 MHz, Chloroform-*d*) δ 153.2, 149.8, 141.8, 140.6, 139.6, 136.1, 135.8, 129.0, 128.8, 128.8, 128.4, 127.3, 127.0, 123.5, 52.3, 41.0, 21.0. HRMS (ESI-TOF) for C_26_H_24_N ([M + H]^+^): calcd. 350.1903; found: 350.1888.

**2-(1-phenyl-2-(p-tolyl)ethyl)quinoline-4-carbonitrile (9)**: Purification by column chromatography (silica gel, PE/EA = 40:1) afforded **9** (7%, 5.1 mg) as a colorless liquid. ^1^H NMR (400 MHz, Chloroform-*d*) δ 8.23 (d, *J* = 8.3 Hz, 1H), 8.09 (dd, *J* = 8.3, 0.7 Hz, 1H), 7.90–7.79 (m, 1H), 7.76–7.65 (m, 1H), 7.52 (s, 1H), 7.38–7.26 (m, 4H), 7.23–7.17 (m, 1H), 6.97 (q, *J* = 8.1 Hz, 4H), 4.54 (t, *J* = 7.7 Hz, 1H), 3.82 (dd, *J* = 13.8, 8.1 Hz, 1H), 3.37 (dd, *J* = 13.8, 7.2 Hz, 1H), 2.24 (s, 3H). ^13^C NMR (100 MHz, Chloroform-*d*) δ 162.7, 147.6, 142.2, 136.8, 135.5, 131.0, 130.2, 129.0, 128.9, 128.7, 128.4, 128.3, 127.0, 126.0, 124.7, 124.3, 118.8, 115.8, 56.0, 40.4, 21.0. HRMS (ESI-TOF) for C_25_H_21_N_2_ ([M + H]^+^): calcd. 349.1699; found: 349.1688.

## 4. Conclusions

In summary, we have reported a visible light-mediated three-component coupling reaction of aryl boronic acids, aryl alkenes, and cyanopyridines for the synthesis of pyridine derivatives. The reaction mechanism was further validated by DFT calculations. We employed readily accessible and easy-to-handle arylboronic acids as aryl precursors. In this transformation, pyridyl and aryl groups were successfully installed onto alkenes to realize their 1,2-difunctionalization. The easy availability of starting materials, mild reaction conditions, simple one-pot procedures, broad functional group tolerance, and the ready scalability of the reaction make these reactions of potential practical synthetic value for synthesizing various natural products, pharmaceuticals, and their derivatives.

## Figures and Tables

**Table 1:** Optimization of the reaction conditions ^a^.

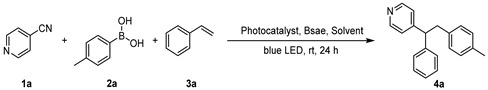				
Entry	Photocatalyst (1.2 mol %)	Solvent	Base	Yield (%) ^b^
1	Ir[dF(CF_3_)ppy]_2_(bpy)PF_6_	MeCN	Ph-COOK	15
2	Ir(dtbbpy)(ppy)_2_]PF_6_	MeCN	Ph-COOK	12
3	3CzClIPN	MeCN	Ph-COOK	27
4	4-CzIPN	MeCN	Ph-COOK	14
5	Ir[dF(Me)ppy]_2_(dtbbpy)PF_6_	MeCN	Ph-COOK	30
6	Ir[dF(Me)ppy]_2_(dtbbpy)PF_6_	DCM	Ph-COOK	N.R.
7	Ir[dF(Me)ppy]_2_(dtbbpy)PF_6_	Acetone	Ph-COOK	16
8	Ir[dF(Me)ppy]_2_(dtbbpy)PF_6_	DMSO	Ph-COOK	N.R.
9	Ir[dF(Me)ppy]_2_(dtbbpy)PF_6_	DMF	Ph-COOK	N.R.
10	Ir[dF(Me)ppy]_2_(dtbbpy)PF_6_	EA	Ph-COOK	17
11	Ir[dF(Me)ppy]_2_(dtbbpy)PF_6_	MeCN	t-BuONa	N.R.
12	Ir[dF(Me)ppy]_2_(dtbbpy)PF_6_	MeCN	DIPEA	N.R.
13	Ir[dF(Me)ppy]_2_(dtbbpy)PF_6_	MeCN	KH_2_PO_4_	35
14	Ir[dF(Me)ppy]_2_(dtbbpy)PF_6_	MeCN	K_2_HPO_4_	N.R.
15	Ir[dF(Me)ppy]_2_(dtbbpy)PF_6_	MeCN	K_3_PO_4_	N.R.
16	Ir[dF(Me)ppy]_2_(dtbbpy)PF_6_	MeCN	NaHCO_3_	38
17	Ir[dF(Me)ppy]_2_(dtbbpy)PF_6_	MeCN	NaH_2_PO_4_	23
18	Ir[dF(Me)ppy]_2_(dtbbpy)PF_6_	MeCN	PhCOOK	30
19 ^c^	Ir[dF(Me)ppy]_2_(dtbbpy)PF_6_	MeCN	NaHCO_3_	66
20 ^d^	Ir[dF(Me)ppy]_2_(dtbbpy)PF_6_	MeCN	NaHCO_3_	73
21 ^e^	-	MeCN	NaHCO_3_	N.R.
22 ^f^	Ir[dF(Me)ppy]_2_(dtbbpy)PF_6_	MeCN	NaHCO_3_	N.R.
23 ^g^	Ir[dF(Me)ppy]_2_(dtbbpy)PF_6_	MeCN	NaHCO_3_	54
24 ^h^	Ir[dF(Me)ppy]_2_(dtbbpy)PF_6_	MeCN	NaHCO_3_	70
25 ^i^	Ir[dF(Me)ppy]_2_(dtbbpy)PF_6_	MeCN	NaHCO_3_	N.R.

^a^ Reaction conditions: **1a** (0.2 mmol), **2a** (3 equiv), **3a** (4 equiv), base (2 equiv), photocatalyst (1.2 mol%), solvent (2 mL), room temperature, 3 W blue LEDs (420 nm), under an Ar atmosphere, and for 24 h. ^b^ Isolated yield. ^c^ **3a** (9 equiv). ^d^ **3a** (9 equiv), MeCN (1 mL). ^e^ Without a photocatalyst. ^f^ In the dark. ^g^ **3a** (9 equiv), MeCN (1 mL), and air atmosphere. ^h^ **3a** (9 equiv), MeCN (1 mL), 3 W violet LEDs (395 nm). ^i^ **3a** (9 equiv), MeCN (1 mL), and 3 W green LEDs (495 nm). “N.R.” stands for “no reaction”.

## Data Availability

The original contributions presented in this study are included in the article/Supplementary Materials. Further inquiries can be directed to the corresponding authors.

## Supplementary Materials

The following supporting information can be downloaded at: https://www.mdpi.com/article/10.3390/molecules31132216/s1 [32,33,34,35,36].

## Abbreviations

The following abbreviations are used in this manuscript:

MeCN	Acetonitrile
DCM	Dichloromethane
DMSO	Dimethyl sulfoxide
DMF	N,N-Dimethylformamide
EA	Ethyl acetate
