# Supplementary material for: Visible-Light-Mediated 1,2-Arylpyridylation of Alkenes Using Arylboronic Acids and Cyanopyridines
Source: Molecules. 2026 Jun 24;31(13):2216. doi: 10.3390/molecules31132216 (PMC13362646; doi:10.3390/molecules31132216)

Supporting Information

Table of Contents

1. General Information S2
2. Optimization Experiments S3
3. Gram-Scale Reaction S6
4. Mechanism Studies S7
5. Theoretical calculations S10
6. X-ray Crystallographic Analysis S26
7. References S28
8. ^{1}H NMR, ^{13}C NMR and ^{19}F NMR Spectra S29

1. General Information

General Remarks. Catalytic reactions were performed under an atmosphere of Ar in a glassware. All solvents were purchased from Energy Chemical, Macklin Biochemical or Sinopharm reagents. All chemicals were used directly without any further purification. Analytical TLC was performed with silica gel GF254 plates. For column chromatography, a 200-300 mesh silica gel was employed. Room temperature (r.t.) is 23-25 °C.

Light source in detail. The light source used for photochemical experiments (**Figure S1**). LED: 3W ($\lambda = 420$ nm). Product model: D800X/H276155-S. Manufacturer: Howsuper Technology Co., Ltd (China). The reaction vessel is borosilicate glass test tube and no filters were applied.

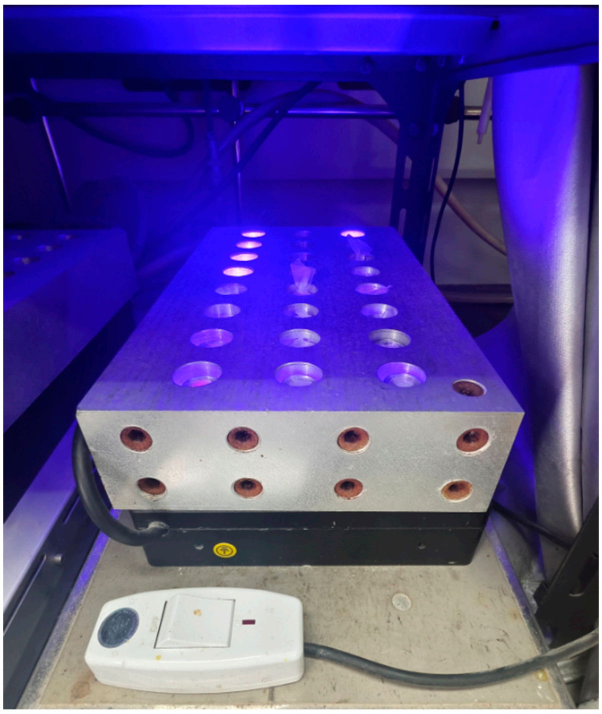

Figure S1. Reaction device

LED fixed lamp (**Figure S2**), 15-40 W ($\lambda = 420 \pm 15$ nm). Product model: PLS-LED 100. Manufacturer: Beijing Perfect light Technology Co., Ltd (China). The reaction vessel is borosilicate glass test tube and no filters were applied. The distance from the light source to the irradiation vessel is 4-5 cm.

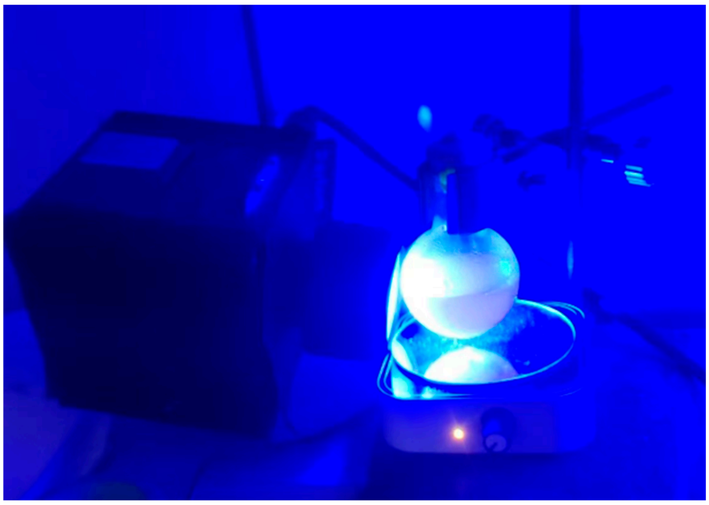

Figure S2. Photoreaction set-up for scale-up reaction.

2. Optimization Experiments

Table S1. Screen of Photocatalyst

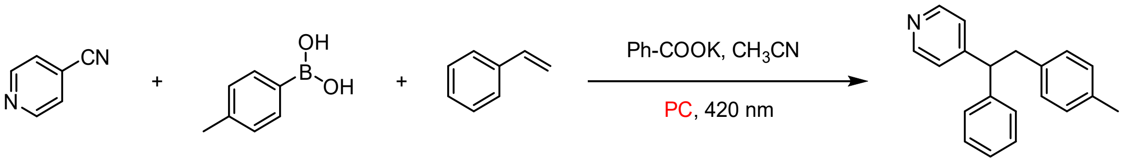

1a **2a** **3a** **4a**

Entry	Photocatalyst (1.2 %)	4a Yield (%)
1	Ir[dF(CF$_3$)ppy]$_2$(bpy)PF$_6$	15
2	Ir[dF(CF$_3$)ppy]$_2$(dtbbpy)PF$_6$	Trace
3	Ir[pF(Me)ppy]$_2$(dtbbpy)PF$_6$	29
4	Ir[dF(Me)ppy]$_2$(dtbbpy)PF$_6$	**30**
5	Ir(dtbbpy)(ppy)$_2$PF$_6$	12
6	Ir(ppy)$_3$	NR
7	Ru(bpy)$_3$Cl$_2$	NR
8	3DPAFIPN	12
9	3CzClIPN	27
10	4-CzIPN	14
11	[Acr-Mes]$^+$(ClO$_4$)$^-$	13

Reaction conditions: **1a** (0.2 mmol), **2a** (3 equiv), **3a** (4 equiv), Ph-COOK (2 equiv), photocatalyst (1.2 mol%), CH_3CN (2 mL), room temperature, 3 W blue LEDs, under an Ar atmosphere, 24 h. Isolated yield.

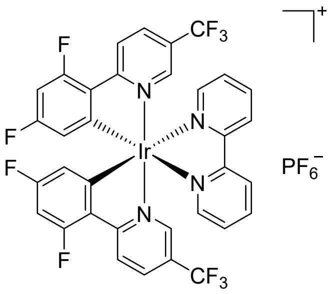

Ir[dF(CF$_3$)ppy]$_2$(bpy)PF$_6$

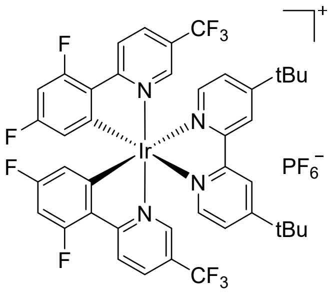

Ir[dF(CF$_3$)ppy]$_2$(dtbbpy)PF$_6$

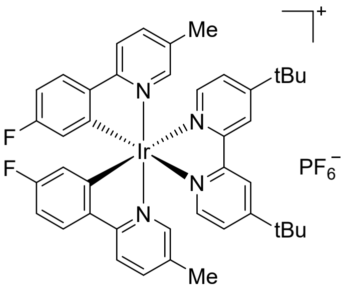

[Ir(pF(Me)ppy)$_2$(dtbbpy)]PF$_6$

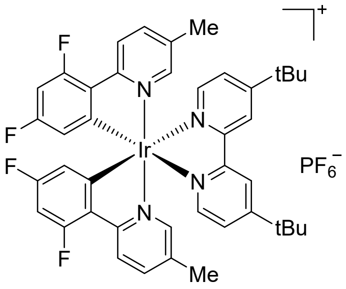

[Ir(dF(Me)ppy)$_2$(dtbbpy)]PF$_6$

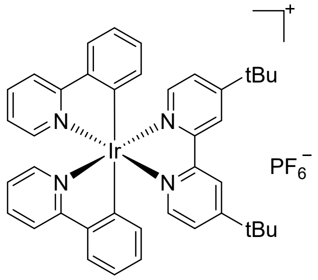

[Ir(dtbbpy)(ppy)$_2$]PF$_6$

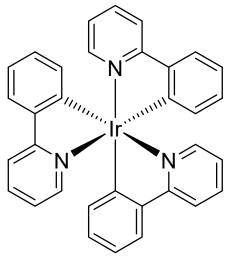

Ir(ppy)$_3$

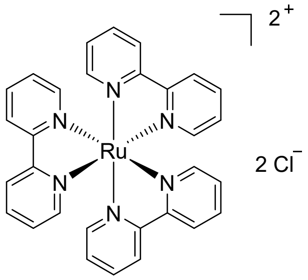

Ru(bpy)$_3$Cl$_2$

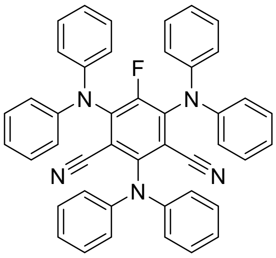

3DPAFIPN

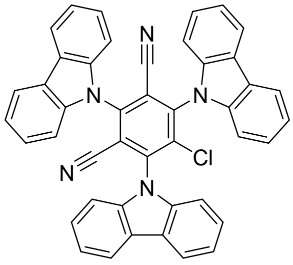

3CzClIPN

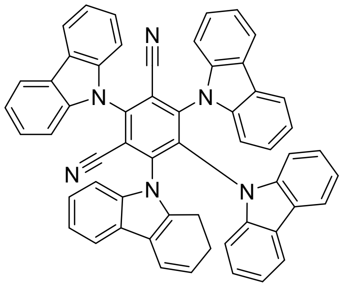

4–CzIPN

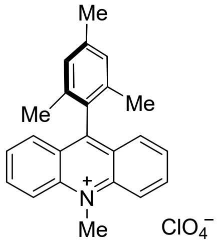

[Acr-Mes]$^+$(ClO$_4$)$^-$

Table S2. Screen of Solvent

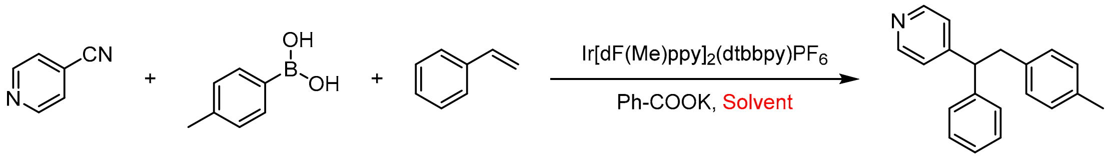

1a 2a 3a 4a

Entry	Solvents	4a Yield (%)
1	CH_3CN	**30**
2	DMF	NR
3	1,4-dioxane	NR
4	THF	NR
5	DMSO	NR
6	Tol	NR
7	DCM	NR
8	EA	17
9	EtOH	NR
10	Acetone	16

Reaction conditions: **1a** (0.2 mmol), **2a** (3 equiv), **3a** (4 equiv), Ph-COOK (2 equiv), Ir[dF(Me)ppy]$_2$(dtbbpy)PF$_6$ (1.2 mol%), Solvent (2 mL), room temperature, 3 W blue LEDs, under an Ar atmosphere, 24 h. Isolated yield.

Table S3. Screen of Base

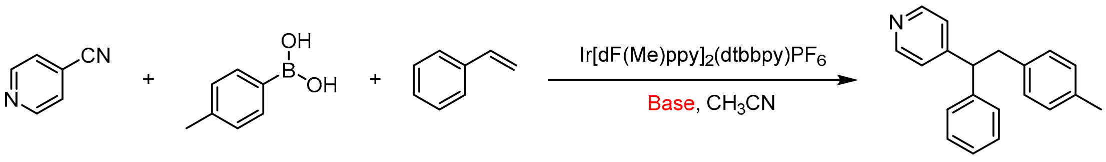

1a **2a** **3a** **4a**

Entry	Base (0.4 mmol）	4a Yield (%)
1	KOH	NR
2	NaOH	NR
3	CsOAc	NR
4	Cs_2CO_3	NR
5	K_2CO_3	NR
6	K_3PO_4	NR
7	KOAc	Trace
8	K_2HPO_4	NR
9	KH_2PO_4	35
10	Na_2CO_3	Trace
11	$NaHCO_3$	**38**

12	t-BuONa	NR
13	t-BuOK	NR
14	NaH_2PO_4	23
15	DIPEA	NR
16	Ph-COOK	30

Reaction conditions: **1a** (0.2 mmol), **2a** (3 equiv), **3a** (4 equiv), Base (2 equiv), Ir[dF(Me)ppy]$_2$(dtbbpy)PF_6 (1.2 mol%), CH_3CN (2 mL), room temperature, 3 W blue LEDs, under an Ar atmosphere, 24 h. Isolated yield.

Table S4. Screen of Ratio[a]

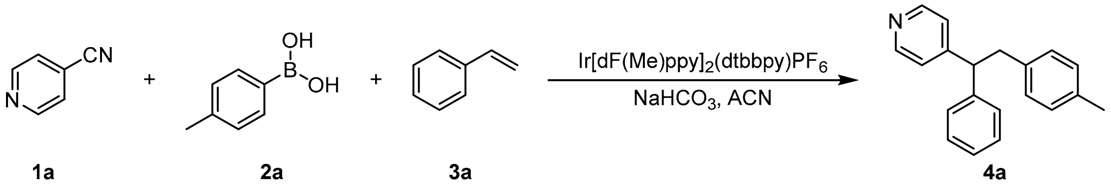

Entry	1a (mmol)	2a (mmol)	3a (mmol)	4a Yield (%)
1	0.2	0.6	0.8	38
2	0.2	0.8	0.8	47
3	0.2	1.0	0.8	49
4	0.2	1.2	0.8	54
5	0.2	1.4	0.8	59
7	0.2	1.8	0.8	66
8[b]	0.2	1.8	0.8	**73**

[a]Reaction conditions: Ir[dF(Me)ppy]$_2$(dtbbpy)PF_6 (1.2 mol%), $NaHCO_3$ (0.4 mmol), CH_3CN (2 mL), room temperature, 3 W blue LEDs, under an Ar atmosphere, 24 h. Isolated yield. [b] CH_3CN (1 mL).

3. Gram-Scale Reaction

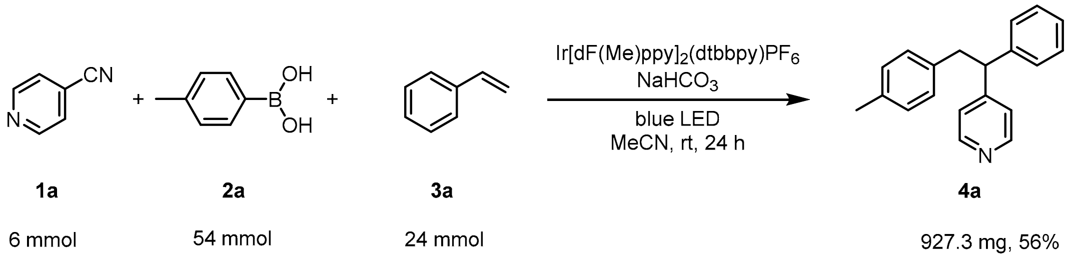

1a (624.7 mg, 6 mmol), **2a** (7.3 g, 54 mmol), **3a** (2.5 g, 24 mmol), $NaHCO_3$ (1.0 g, 12 mmol) and Ir[dF(Me)ppy]$_2$(dtbbpy)PF_6 (1.2 mol%) were dissolved in CH_3CN (30 mL) in a 250 mL round bottom flask. The mixture was purged with Ar to create an inert atmosphere. The reactants were mixed evenly and then irradiated with a 30 W blue LEDs for 24 h. After the reaction, it was then extracted with ethyl acetate (3×100 mL), and the combined organic extract was washed with saline (100 mL), dried with anhydrous sodium sulfate and concentrated in vacuo. The crude mixture was directly purified by column chromatography on silica gel (DCM:MeOH = 200:1) by flash column chromatography to obtain the desired product **4a** (927.3 mg, 56%).

4. Mechanism Studies

4.1 TEMPO trapping experiment

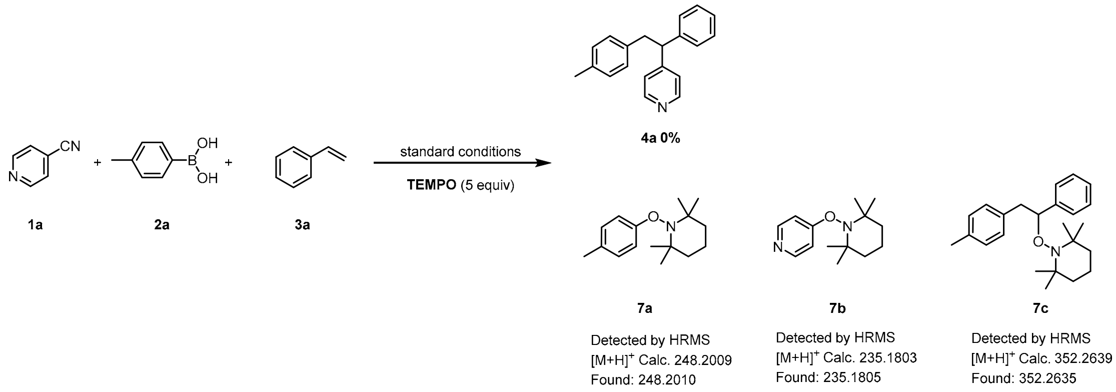

CH_3CN (1.0 mL) was added to a 4 mL transparent glass bottle with magnetic stirring bars and rubber plugs, then, **TEMPO** (1.0 mmol, 5.0 equiv), **1a** (0.2 mmol, 1.0 equiv), **2a** (1.8 mmol, 9.0 equiv), **3a** (0.8 mmol, 4.0 equiv), $NaHCO_3$ (0.4 mmol, 2.0 equiv) and Ir[dF(Me)ppy]$_2$(dtbbpy)PF_6 (1.2 mol%) were added to the bottle. The mixture was purged with argon to create an inert atmosphere. The reaction mixture was irradiated with 3 W blue LEDs (420 nm, 100% intensity) for 24 hours at ambient temperature. TLC analysis indicates that no product 4a, and the TEMPO-trapped product **7a/7b/7c** was obtained by HRMS.

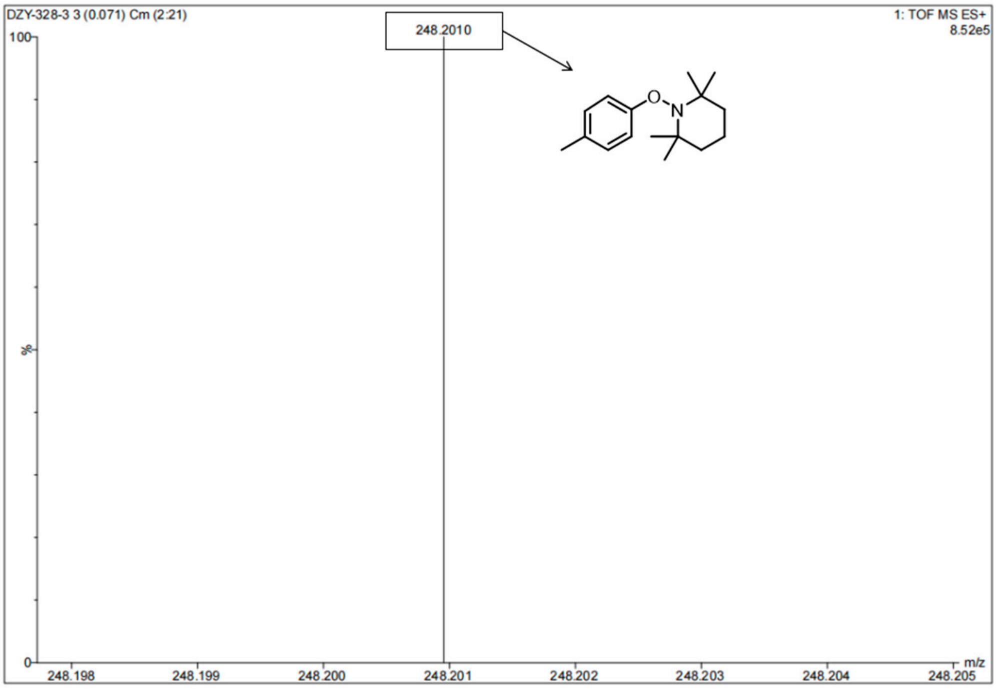

Figure S3. HRMS of 7a

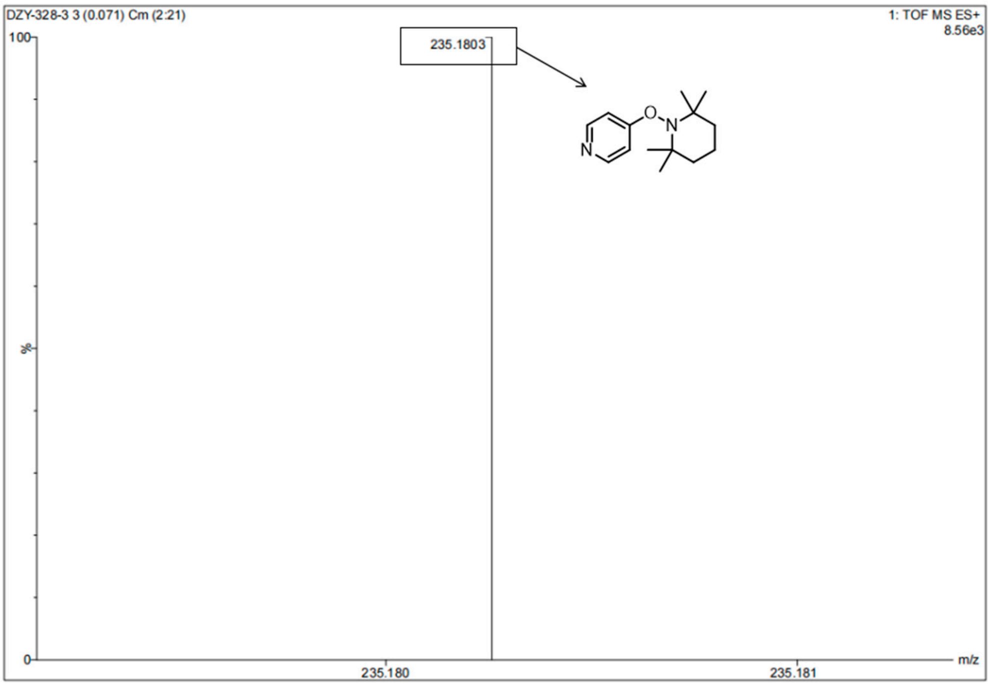

Figure S4. HRMS of 7b

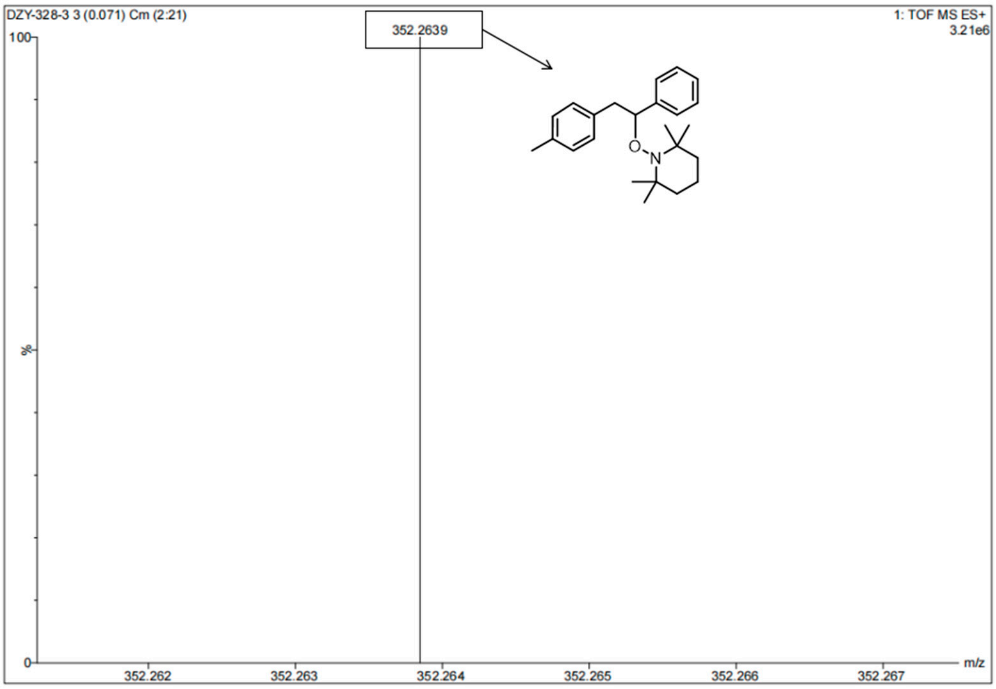

Figure S5. HRMS of 7c

4.2 BHT trapping experiment

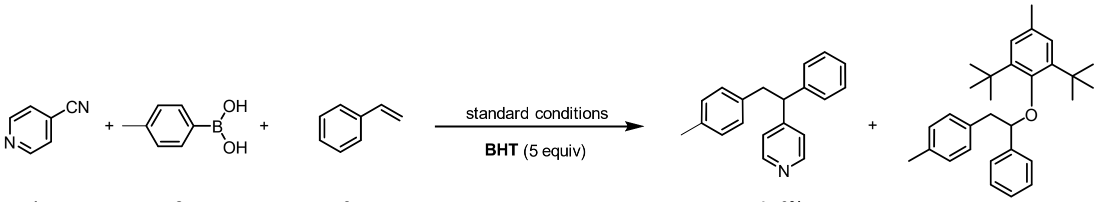

CH_3CN (1.0 mL) was added to a 4 mL transparent glass bottle with magnetic stirring bars and rubber plugs, then, **BHT** (1.0 mmol, 5.0 equiv), **1a** (0.2 mmol, 1.0 equiv), **2a** (1.8 mmol, 9.0 equiv), **3a** (0.8 mmol, 4.0 equiv), $NaHCO_3$ (0.4 mmol, 2.0 equiv) and Ir[dF(Me)ppy]$_2$(dtbbpy)PF_6 (1.2 mol%) were added to the bottle. The mixture was purged with Ar to create an inert atmosphere. The reaction mixture was irradiated with 3 W blue LEDs (420 nm, 100% intensity) for 24 hours at ambient temperature. After the reaction (monitored by TLC), TLC analysis indicates that no product 4a was produced, and the BHT-trapped product **8** was obtained by HRMS.

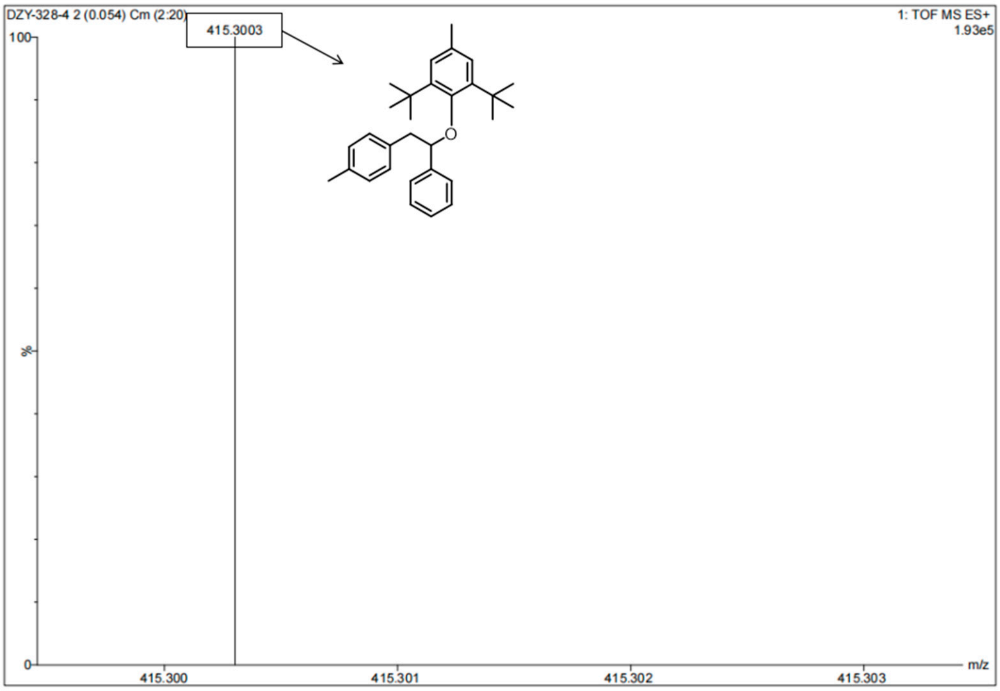

Figure S6. HRMS of 8

5. Theoretical calculations

General information

Density functional theory (DFT) calculations were carried out using Gaussian 09 program.[1] Geometrical optimization calculations were carried out at the PBE0-D3(BJ)[2, 3]/def2-TZVP[4] level with the SMD continuum solvent model[5] for acetonitrile without any symmetry assumptions unless otherwise stated. Harmonic vibration frequency calculations were performed at the same level for verifying the resulting geometries as local minima (with all the frequencies real) or saddle points (with only one imaginary frequency).

Energies of Stationary Points

Supplementary Table S5. Electronic energies(***EE***), thermal corrections to Gibbs free energies (***Gcorr***), and Gibbs free energies(***G***) of the stationary points calculated at the PBE0-D3(BJ)/def2-TZVP level of theory with the SMD continuum solvent model for acetonitrile.

Table S5

	G_{corr}(Ha)	*EE*(Ha)	*G*(Ha)
**2a**	0.117620	-447.267329	-447.149709
**HCO_3^-**	0.000812	-264.386126	-264.385314
**Int-1**	0.141257	-711.667092	-711.525835
**$HCO_3B(OH)_2$**	0.033496	-440.801787	-440.768291
**Int-2**	0.085344	-270.662533	-270.577190
**3a**	0.102549	-309.400646	-309.298097
**Ts-1**	0.202097	-580.066461	-579.864364

**Int-3**	0.207999	-580.144883	-579.936884
**1a**	0.057708	-340.267884	-340.210177
**Ts-2**	0.287993	-920.526238	-920.238246
**Int-4**	0.292163	-920.546436	-920.254273
**CN^-**	-0.014051	-92.880289	-92.894340
**4a**	0.28628	-827.698683	-827.412403
**Int-5**	0.053877	-340.360668	-340.306791
**Ir(III)**	0.649236	-2345.786293	-2345.137057
**Ir(III)***	0.643968	-2345.689905	-2345.045938
**Ir(IV)**	0.646358	-2345.578772	-2344.932414

Cartesian coordinates of the optimized geometries

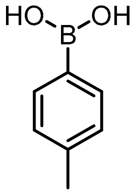

2a

C	0.06580700	1.19521700	-0.01573500
C	1.45324100	1.19820800	-0.01735600
C	2.17127500	0.00516700	-0.00032400
C	1.45480900	-1.19272600	0.01664400
C	0.07067400	-1.19303000	0.01548300
C	-0.65768000	0.00144900	0.00005200
C	3.66747800	-0.00145000	0.00045000
B	-2.21945900	-0.00109300	0.00040400
O	-2.86765800	1.20265800	0.03435600
O	-2.86297600	-1.20732500	-0.03396700
H	-0.46642100	2.14079300	-0.02873700
H	1.99174200	2.14084500	-0.03152600
H	1.99542700	-2.13453900	0.03080100
H	-0.46026400	-2.13933700	0.02856600
H	4.06903000	1.01307600	-0.00591100
H	4.05605100	-0.51759600	0.88327900
H	4.05735700	-0.52910800	-0.87493900
H	-3.82930500	1.14347000	0.02736600
H	-3.82486800	-1.15180500	-0.02931900

HCO_3^- 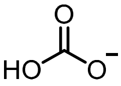

C	-0.13848800	0.05854400	-0.00001800
O	1.01891200	-0.73458000	0.00000500
O	-1.19520900	-0.58397600	0.00000400
O	0.06163500	1.28768200	0.00000500
H	1.74822000	-0.10427100	-0.00001100

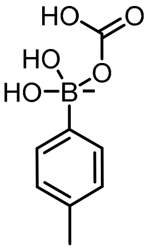

Int-1

C	2.99510000	-0.56753000	-0.39055900
O	3.47186600	-1.77481100	-0.75267400
O	1.74666300	-0.62522600	-0.14553600

O	3.72724500	0.38642000	-0.34835700
B	0.98256700	0.61390400	0.42008300
C	-0.59318400	0.29406000	0.18564700
O	1.36615300	0.61133300	1.83416400
O	1.35537800	1.81512600	-0.28922900
C	-1.21446600	-0.79294900	0.80445200
C	-2.56505800	-1.07185000	0.63130700
C	-3.36734100	-0.26347500	-0.17118500
C	-2.76313700	0.82722300	-0.79394600
C	-1.41022000	1.09171500	-0.61947000
C	-4.82694700	-0.54897400	-0.35757100
H	4.41200300	-1.65041900	-0.94155800
H	1.09692500	1.44824800	2.21868600
H	2.32044500	1.83162300	-0.29563500
H	-0.62577100	-1.44289700	1.44684500
H	-3.00937100	-1.93017000	1.12864000
H	-3.36371500	1.47635400	-1.42663600
H	-0.96810200	1.94655800	-1.12265200
H	-5.12225600	-1.45853000	0.16924900
H	-5.44392300	0.27279300	0.01914500
H	-5.07598400	-0.67515100	-1.41549400

$HCO_3B(OH)_2$ 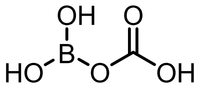

C	1.22938800	0.11839300	0.00000000
O	2.08530400	1.13895000	0.00000000
O	0.00000000	0.62212400	0.00000000
O	1.54896800	-1.03711600	0.00000000
B	-1.20971500	-0.07939800	0.00000000
O	-2.27822900	0.75797100	0.00000000
O	-1.23700200	-1.43070600	0.00000000
H	2.97976700	0.76394000	0.00000000
H	-3.13363800	0.31578300	0.00000000
H	-2.12621200	-1.80287200	0.00000000

Int-2 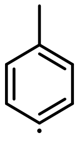

C	0.13402800	-1.19923000	-0.00000400
C	-1.25968300	-1.22163600	0.00000100
C	-1.89074200	-0.00519200	0.00000400
C	-1.27129700	1.21425200	0.00000100
C	0.12562900	1.20315800	-0.00000400
C	0.83863700	0.00617400	-0.00000600

C	2.33726000	0.00416900	0.00000400
H	0.68061700	-2.13797800	-0.00001000
H	-1.80118700	-2.16143200	0.00000300
H	-1.81986100	2.14997900	-0.00000100
H	0.66496600	2.14566200	-0.00000700
H	2.73292000	1.02089700	-0.00017700
H	2.72977200	-0.51348900	0.87974900
H	2.72978600	-0.51381400	-0.87954200

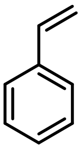

3a

C	0.01483800	1.08555600	-0.00000500
C	-1.34824100	1.32407700	-0.00000200
C	-2.24900000	0.26370900	0.00000400
C	-1.77154000	-1.03866800	0.00000400
C	-0.40492700	-1.27674400	0.00000000
C	0.51114800	-0.22237500	-0.00000400
C	1.94273400	-0.53302800	-0.00000700
C	2.95100400	0.33730200	0.00000900
H	0.70179400	1.92440800	-0.00001100
H	-1.71410900	2.34509600	-0.00000400
H	-3.31626000	0.45513500	0.00000600
H	-2.46415500	-1.87323300	0.00000800
H	-0.03290600	-2.29648100	0.00000100
H	2.17426900	-1.59619800	-0.00002000
H	3.97827500	-0.00930400	0.00000600
H	2.79700300	1.41160600	0.00002500

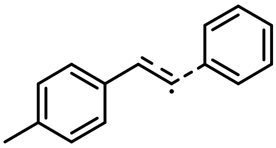

Ts-1

C	2.76449500	1.46507700	0.41770500
C	3.83099400	0.56394900	0.37768400
C	3.66416900	-0.72994900	-0.11141400
C	2.40427500	-1.11983600	-0.56937800
C	1.32318500	-0.24103100	-0.54108600
C	1.55751400	1.01626500	-0.04711700
C	-0.77883500	2.47321000	0.26877900
C	-1.65698400	1.82895700	-0.50689600
C	-2.41076800	0.62880800	-0.15360300
C	-2.24007000	-0.04487900	1.06150900

C	-2.97012100	-1.18529900	1.34609700
C	-3.88699100	-1.68309200	0.42554500
C	-4.06457900	-1.02821800	-0.78491100
C	-3.33207200	0.11424000	-1.07023200
C	4.81056000	-1.69509000	-0.13708900
H	2.90468200	2.47080800	0.80059300
H	4.80797200	0.87796100	0.73365800
H	2.26398300	-2.12592900	-0.95480400
H	0.34606000	-0.55052500	-0.89729900
H	-0.58537800	2.18366200	1.29547300
H	-0.27269200	3.36146000	-0.09070600
H	-1.83139200	2.19878900	-1.51478800
H	-1.52401000	0.32382100	1.78730200
H	-2.82224700	-1.69477200	2.29215600
H	-4.45584600	-2.57796600	0.65211700
H	-4.77453500	-1.40897500	-1.51102600
H	-3.47000100	0.62369200	-2.01880000
H	5.74984700	-1.19922400	0.11306200
H	4.65690300	-2.50558200	0.58198500
H	4.91802200	-2.15589500	-1.12246900

Int-3 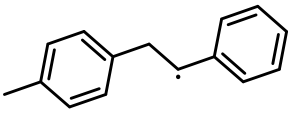

C	-2.30187100	1.09561200	0.81239900
C	-3.63831300	0.71514700	0.86126600
C	-4.14496100	-0.24374500	-0.00873200
C	-3.26709400	-0.81115100	-0.93403700
C	-1.93616000	-0.43264100	-0.98320500
C	-1.43050700	0.52948800	-0.10905500
C	0.01938400	0.91828900	-0.15703000
C	0.93177000	-0.16576900	0.30292500
C	2.33319200	-0.14063700	0.15948400
C	3.01881300	0.93624600	-0.45354000
C	4.39456100	0.92429200	-0.57374100
C	5.14125800	-0.14948800	-0.09342300
C	4.48729600	-1.22102700	0.51550800
C	3.11455000	-1.21998400	0.64160400
C	-5.58331600	-0.65824600	0.03677600
H	-1.93166200	1.84676300	1.50350200
H	-4.29891300	1.17347600	1.59084800
H	-3.63693400	-1.56009800	-1.62818200
H	-1.27473400	-0.88853700	-1.71408800
H	0.28479300	1.21199300	-1.18127000

H	0.17211900	1.82038000	0.45145700
H	0.49255700	-1.02310000	0.80381600
H	2.45640300	1.78191500	-0.83221300
H	4.89734300	1.76105400	-1.04710300
H	6.22079500	-0.15202800	-0.19174900
H	5.06142300	-2.06072200	0.89253300
H	2.60971800	-2.05539700	1.11641100
H	-6.12508600	-0.11998500	0.81634000
H	-6.08049700	-0.46411100	-0.91819800
H	-5.67893700	-1.72993000	0.23470300

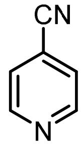

1a

C	0.11045100	-1.20156900	-0.00000300
C	1.49370300	-1.14063000	0.00000100
N	2.17889800	0.00000500	0.00000500
C	1.49369400	1.14063700	0.00000100
C	0.11044400	1.20156500	-0.00000300
C	-0.59064300	-0.00000600	-0.00000500
C	-2.01822600	-0.00000900	-0.00001100
N	-3.16912800	0.00000500	0.00001200
H	-0.40671600	-2.15214100	-0.00000400
H	2.07426700	-2.05781200	0.00000300
H	2.07425400	2.05782100	0.00000300
H	-0.40673200	2.15213200	-0.00000400

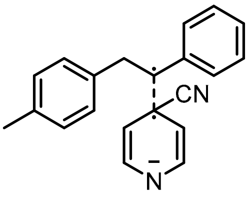

Ts-2

C	3.77673200	-1.61756700	-0.74283200
C	4.66220200	-0.60306100	-0.37410800
C	4.19734100	0.37208800	0.50172800
C	2.89526700	0.34258400	0.98826000
C	2.00938800	-0.66196000	0.61080000
C	2.47832600	-1.64485900	-0.26232100
C	6.06078500	-0.57090200	-0.90990800
C	0.57850700	-0.67076900	1.08387100
C	-0.36681100	-0.15760800	0.04260900
C	-2.49320500	-0.92287700	0.68008600
C	-0.54996100	1.21865600	-0.23556400
C	-1.19600400	1.61812000	-1.43445600

C	-1.50643100	2.94135600	-1.68870100
C	-1.18326600	3.93954900	-0.77230400
C	-0.53101500	3.57633400	0.40539900
C	-0.21617900	2.25675900	0.67168900
C	-3.39163000	-0.38946800	-0.28971600
C	-3.82590400	-1.20496800	-1.30599900
N	-3.54203800	-2.51119600	-1.42581200
C	-2.82087500	-3.04004700	-0.41909700
C	-2.32973500	-2.33987000	0.65179500
C	-2.25655000	-0.20162000	1.87793000
N	-2.09609800	0.38656100	2.86245400
H	4.11520300	-2.40017300	-1.41597500
H	4.86694700	1.16802900	0.81419900
H	2.56411800	1.11310100	1.67734800
H	1.81042500	-2.44546900	-0.56771400
H	6.06618100	-0.38452300	-1.98852500
H	6.64879700	0.21484800	-0.43207900
H	6.57070000	-1.52482300	-0.74813500
H	0.29865100	-1.70086400	1.33823200
H	0.50075700	-0.08293600	2.00337800
H	-0.51705200	-0.82391000	-0.80238800
H	-1.43400200	0.85856200	-2.17192900
H	-2.00050400	3.20256700	-2.61996000
H	-1.41870300	4.97784200	-0.97716300
H	-0.25837300	4.34135800	1.12653400
H	0.29548600	2.01386200	1.59541000
H	-3.69286300	0.64870800	-0.24948300
H	-4.46624400	-0.78356100	-2.07829400
H	-2.64506200	-4.11230200	-0.47186400
H	-1.78340000	-2.84709900	1.43695900

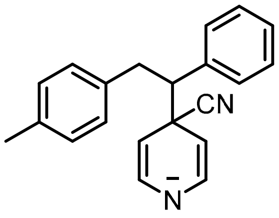

Int-4

C	3.76416400	-1.59871600	-0.84844200
C	4.67476600	-0.64073000	-0.37406400
C	4.21943900	0.26068700	0.59302600
C	2.90600500	0.21379000	1.06675400
C	1.99635200	-0.73467200	0.58687500
C	2.45504100	-1.64285900	-0.38014700
C	6.08280500	-0.59578800	-0.89198100
C	0.55672700	-0.75594900	1.04496800

C	-0.38739600	-0.19916700	0.01977700
C	-2.53663300	-0.86963400	0.69101700
C	-0.51949500	1.19151600	-0.24750600
C	-1.17173000	1.63091300	-1.43742700
C	-1.43026500	2.97543400	-1.67693600
C	-1.04671900	3.95620200	-0.75474200
C	-0.39035400	3.55305600	0.41558800
C	-0.12764800	2.21217800	0.66790700
C	-3.42177600	-0.30101700	-0.28112500
C	-3.89813400	-1.10899000	-1.29653900
N	-3.67264600	-2.42789100	-1.40665000
C	-2.96995600	-2.98556300	-0.40233100
C	-2.43863600	-2.29956800	0.67007300
C	-2.25317600	-0.14733100	1.88746500
N	-2.04130800	0.44526700	2.86840400
H	4.09324300	-2.32465500	-1.59880000
H	4.90723800	1.01460500	0.98834400
H	2.58324300	0.92772100	1.83012400
H	1.76724300	-2.39962100	-0.77103700
H	6.10434100	-0.38623000	-1.97425500
H	6.67256600	0.18250300	-0.38652000
H	6.59554900	-1.56038900	-0.74522300
H	0.26874100	-1.80085500	1.26024400
H	0.47356200	-0.20234300	1.99499400
H	-0.58062600	-0.85644300	-0.83408300
H	-1.45463200	0.88340300	-2.18358600
H	-1.93132700	3.26787100	-2.60531000
H	-1.24198200	5.01378400	-0.94953300
H	-0.07213100	4.30516900	1.14464800
H	0.38728300	1.93676100	1.59062900
H	-3.67926800	0.75804100	-0.24493500
H	-4.52807500	-0.65794500	-2.07481300
H	-2.83996500	-4.07473700	-0.44858100
H	-1.90762000	-2.83101700	1.46217000

CN^-

C	0.00000000	0.00000000	-0.62804500
N	0.00000000	0.00000000	0.53832400

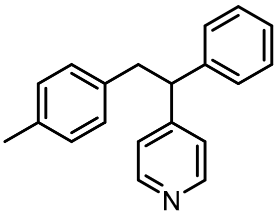

4a

C	3.42473200	-1.71140200	0.81314600
C	4.22998500	-0.85871400	0.05667900
C	3.63168900	-0.15602100	-0.98378200
C	2.27689200	-0.29567200	-1.25923000
C	1.47562500	-1.14317600	-0.50312000
C	2.07450000	-1.85022700	0.53905800
C	5.69004700	-0.71598700	0.35751800
C	0.00240900	-1.26010500	-0.75934100
C	-0.82692000	-0.47969300	0.27583400
C	-0.50133400	0.99334600	0.20833600
C	-2.31576900	-0.71744700	0.16216200
C	-3.09597100	-0.64908700	1.31537600
C	-4.46950400	-0.83040000	1.26123000
C	-5.08992600	-1.08568200	0.04492600
C	-4.32354800	-1.15915400	-1.10912700
C	-2.94742800	-0.97744600	-1.05100000
C	-0.87640700	1.77862700	-0.87705300
C	-0.51232800	3.11330200	-0.90446200
N	0.18720200	3.70959900	0.06132400
C	0.54253800	2.95734600	1.09961400
C	0.22575000	1.61261800	1.21634500
H	3.86658100	-2.27584000	1.62903000
H	4.23494800	0.51334400	-1.58942700
H	1.83442500	0.26750100	-2.07503600
H	1.47047500	-2.51963100	1.14458900
H	5.85095400	-0.39961200	1.39214200
H	6.21396100	-1.66795800	0.22911200
H	6.15880200	0.01864400	-0.29937000
H	-0.22211200	-0.89424600	-1.76372300
H	-0.30483300	-2.30899500	-0.71457400
H	-0.52117900	-0.82611600	1.26709600
H	-2.61433000	-0.45206100	2.26833900
H	-5.05680900	-0.77652100	2.17153300
H	-6.16357100	-1.23063000	-0.00150200
H	-4.79718500	-1.36199700	-2.06366300
H	-2.36792900	-1.04126700	-1.96450200
H	-1.44652700	1.36031900	-1.69865000
H	-0.79812100	3.73581600	-1.74804400
H	1.11259600	3.45017500	1.88264600
H	0.54925500	1.05172800	2.08608400

Int-5 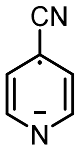

C	-0.07328000	1.22120000	-0.00039400
C	-1.42638100	1.19171200	-0.00007500
N	-2.10018800	-0.00008500	0.00084500
C	-1.42624300	-1.19180500	-0.00007500
C	-0.07313900	-1.22112500	-0.00039300
C	0.66985000	0.00008200	-0.00016100
C	2.06408000	0.00018700	0.00009400
N	3.22806200	-0.00010700	0.00035700
H	0.43718400	2.17518700	-0.00084200
H	-2.03578900	2.08462000	0.00033900
H	-2.03554700	-2.08478400	0.00033900
H	0.43744100	-2.17505000	-0.00084200

Ir(III)

Ir	-0.69074400	-0.00473300	-0.00700000
C	-1.89014500	2.21462900	-3.40976000
C	-1.73853200	1.69246100	-2.12580700
N	-0.84037700	0.69902100	-1.92040300
C	-0.11035700	0.22808800	-2.93604800
C	-0.21150800	0.70119900	-4.23048400
C	-1.13249400	1.72231400	-4.45067700
C	0.92267700	2.58658800	0.52555400
N	0.99357600	1.28203400	0.25125000
C	2.20362500	0.71569900	0.10783700
C	3.36762800	1.45818100	0.25508600
C	3.31273700	2.81188900	0.55590900
C	2.04154700	3.37235400	0.68380800
N	0.98429800	-1.28997100	-0.32907300
C	0.90903700	-2.59008300	-0.60123800
C	2.02676100	-3.38581400	-0.76305500
C	3.29568700	-2.83198500	-0.64053200
C	3.35584200	-1.47020100	-0.35315600
C	2.20110800	-0.72388000	-0.20046900
C	-1.63596700	-1.70566700	2.15702400
C	-1.72756100	-2.22875100	3.44629700
C	-0.92422600	-1.73534200	4.45191700
C	-0.01688100	-0.71191400	4.19015100
C	0.02273600	-0.23686900	2.89318300
N	-0.75169100	-0.70879600	1.91140600
C	-3.76161800	-2.71540700	-1.33671800
C	-4.05960600	-3.44248000	-0.20059500
C	-3.36746300	-3.11272600	0.94424100
C	-2.40858000	-2.10361300	0.98875500
C	-2.12868500	-1.38426200	-0.19929800
C	-2.82361700	-1.70447400	-1.36315700

C	-4.06057000	3.41989800	0.33854700
C	-3.70859100	2.69428800	1.46008500
C	-2.76600100	1.68742400	1.44448500
C	-2.12269900	1.37001800	0.25050200
C	-2.45899000	2.08790600	-0.92382000
C	-3.41926200	3.09286200	-0.83625600
F	-3.76183300	3.80185400	-1.92481100
F	-4.32004300	2.99175900	2.61788300
F	-4.42282000	-3.01584100	-2.46605400
F	-3.65735100	-3.82357700	2.04681700
C	0.87146500	-0.14245000	5.24525200
C	4.57449000	-3.63383700	-0.80124700
C	5.39313800	-3.03613200	-1.94980500
C	5.37906100	-3.54713500	0.49965000
C	4.29399700	-5.09927200	-1.10956500
C	4.55254900	3.66591700	0.74623300
C	5.83721100	2.86549200	0.57306200
C	4.53261800	4.80075200	-0.28223300
C	4.52621700	4.25807500	2.15902300
C	0.62683600	0.13336600	-5.32678600
H	-2.60376600	3.00331900	-3.58821000
H	0.58141800	-0.56870200	-2.69648600
H	-1.25737600	2.13296800	-5.44701600
H	-0.07306300	2.99938600	0.62686000
H	4.32291600	0.97066600	0.13356900
H	1.91032200	4.42299400	0.90917900
H	-0.08790300	-3.00220800	-0.69483700
H	1.88364600	-4.43465400	-0.98227100
H	4.31701500	-0.98770700	-0.24314700
H	-2.42992300	-3.01977900	3.65643200
H	-1.00224600	-2.14691000	5.45262000
H	0.70015500	0.56274800	2.62408500
H	-4.79981700	-4.23174400	-0.20133200
H	-2.64897700	-1.18029800	-2.29484800
H	-4.80362600	4.20572600	0.37269800
H	-2.54821900	1.16407100	2.36763600
H	1.47104200	-0.92704200	5.71404100
H	1.54843400	0.60576900	4.83055600
H	0.28223900	0.33055800	6.03574300
H	6.31888700	-3.60367100	-2.07366000
H	4.83829500	-3.07917100	-2.89067400
H	5.66119200	-1.99493200	-1.75855400
H	6.30782500	-4.11355200	0.39432800
H	4.81606000	-3.96751300	1.33693200

H	5.64108600	-2.51623100	0.74782500
H	3.72980100	-5.58248000	-0.30783500
H	3.73849800	-5.21823200	-2.04343400
H	5.24177900	-5.63126100	-1.21676100
H	6.69385500	3.52735500	0.71725100
H	5.91526600	2.05815700	1.30612500
H	5.91627700	2.43413300	-0.42838800
H	5.41583400	5.43069900	-0.14969500
H	3.64900100	5.43213200	-0.16944300
H	4.54406200	4.40690000	-1.30185000
H	4.53517700	3.46890400	2.91545700
H	5.40873900	4.88479400	2.31005100
H	3.64149700	4.87719600	2.32139800
H	1.21250000	0.91725100	-5.81412600
H	1.31488400	-0.62369900	-4.94801900
H	0.00057300	-0.32789800	-6.09536700

Ir(III)*

Ir	-0.75033800	-0.00195200	0.00005300
C	-1.85101200	2.24743100	-3.40598000
C	-1.72908200	1.70995300	-2.12777600
N	-0.86535600	0.68788500	-1.92039300
C	-0.12635500	0.20604200	-2.91923100
C	-0.19550800	0.69821300	-4.21188500
C	-1.08800500	1.74145600	-4.43813800
C	0.86300900	2.61036000	0.48294300
N	0.92290800	1.29094400	0.23952100
C	2.15870900	0.69578800	0.10285200
C	3.32647500	1.48136600	0.26151500
C	3.26396000	2.82121000	0.52758700
C	1.96764200	3.40057900	0.63273400
N	0.92290600	-1.28654900	-0.30836700
C	0.86166900	-2.60120600	-0.55532100
C	1.96710800	-3.39467500	-0.72007500
C	3.25751200	-2.81706900	-0.62465600
C	3.32065400	-1.47278300	-0.36060000
C	2.15924800	-0.68741500	-0.18921300
C	-1.58589100	-1.74290600	2.16455800
C	-1.62299800	-2.29658600	3.44113100
C	-0.81409300	-1.78405400	4.43469700
C	0.03994100	-0.71772000	4.17104700
C	0.02402100	-0.20996600	2.88278900
N	-0.75812200	-0.69941100	1.92139100
C	-3.65243700	-2.84618500	-1.35360900
C	-3.94200700	-3.55411500	-0.19868100

C	-3.28039800	-3.19567000	0.95770600
C	-2.34413800	-2.16899000	0.99786400
C	-2.09007500	-1.45284000	-0.20573000
C	-2.74755500	-1.81071900	-1.38435600
C	-3.97877200	3.50308600	0.35007500
C	-3.61738300	2.80516200	1.49107500
C	-2.69786200	1.78260400	1.47775100
C	-2.09981600	1.42773500	0.26661000
C	-2.42981300	2.13275200	-0.92504600
C	-3.37587100	3.14747600	-0.83883600
F	-3.74103900	3.83279000	-1.92722100
F	-4.19884100	3.14843800	2.64386100
F	-4.28989300	-3.19264200	-2.47544700
F	-3.57785300	-3.89031100	2.06074900
C	0.93716000	-0.13656500	5.20991800
C	4.53372900	-3.63009600	-0.79942000
C	5.34946900	-3.05038000	-1.95851900
C	5.35959600	-3.55324700	0.48818200
C	4.24154700	-5.09613800	-1.10031600
C	4.49246400	3.69269600	0.71313400
C	5.78908300	2.90514100	0.57332400
C	4.47912800	4.80563500	-0.33955700
C	4.45074800	4.32077500	2.11018500
C	0.64999100	0.12324200	-5.29678100
H	-2.53713700	3.05937300	-3.58822100
H	0.54228400	-0.60714000	-2.66855600
H	-1.18440300	2.16375400	-5.43248400
H	-0.13038100	3.03192400	0.57445000
H	4.28562600	0.99355600	0.16590200
H	1.83287300	4.45425300	0.83694000
H	-0.13198100	-3.02488400	-0.63455300
H	1.82360000	-4.44542100	-0.92519800
H	4.28434500	-0.98811100	-0.27571000
H	-2.27824500	-3.12695000	3.65193600
H	-0.84373100	-2.21956200	5.42754700
H	0.65759800	0.62213200	2.60459300
H	-4.66175200	-4.36305000	-0.19657200
H	-2.56688800	-1.29323500	-2.31788800
H	-4.70854600	4.30229600	0.38329500
H	-2.46027200	1.27213800	2.40244100
H	1.60993300	-0.90033700	5.60876400
H	1.54064800	0.67466900	4.80156000
H	0.35575800	0.25365200	6.04955400
H	6.26757500	-3.62881700	-2.09552600

H	4.78227100	-3.08633900	-2.89264500
H	5.63120200	-2.01180200	-1.77251400
H	6.27991700	-4.13392100	0.37770900
H	4.80065100	-3.95905400	1.33573600
H	5.63707900	-2.52413400	0.72671200
H	3.68475400	-5.57433100	-0.29038000
H	3.67190200	-5.21472200	-2.02570200
H	5.18439700	-5.63554900	-1.21907100
H	6.63872200	3.57784300	0.71330600
H	5.86523600	2.11317300	1.32315400
H	5.88378900	2.45181200	-0.41700900
H	5.34947500	5.45432500	-0.20725500
H	3.58343200	5.42537500	-0.26011700
H	4.51520100	4.38846000	-1.34958700
H	4.46400300	3.54960800	2.88532700
H	5.32236000	4.96488800	2.25565900
H	3.55547000	4.92991900	2.25242000
H	1.23613700	0.90616100	-5.78449400
H	1.33551700	-0.63039600	-4.90792300
H	0.02717400	-0.34206200	-6.06587900

Ir(IV)

Ir	-0.75210500	-0.00622400	-0.00262200
C	-1.79818400	2.34071500	-3.36261500
C	-1.68731600	1.77172300	-2.09769000
N	-0.85090800	0.72230000	-1.91467600
C	-0.13721600	0.23482900	-2.92885800
C	-0.20000500	0.75485700	-4.21144000
C	-1.05728900	1.83250500	-4.40977100
C	0.89207300	2.58006000	0.53076800
N	0.96359200	1.27373900	0.25677800
C	2.17217400	0.70606700	0.11718800
C	3.33594100	1.44638800	0.25892200
C	3.28218100	2.80139500	0.55435500
C	2.01213900	3.36278100	0.68507500
N	0.94969200	-1.29431400	-0.30608300
C	0.86824900	-2.59648100	-0.57530100
C	1.98417200	-3.39257200	-0.73700300
C	3.25421200	-2.84130000	-0.61801100
C	3.31866700	-1.47906600	-0.33373900
C	2.16701900	-0.73108200	-0.18320700
C	-1.63540100	-1.77806300	2.12006400
C	-1.70765800	-2.35063400	3.38618100
C	-0.92332100	-1.85571800	4.40780700
C	-0.06049500	-0.78792800	4.18234100

C	-0.03838000	-0.26294300	2.90043800
N	-0.79446300	-0.73721000	1.91104400
C	-3.66377300	-2.76568000	-1.45183700
C	-3.96226100	-3.51422500	-0.32366900
C	-3.31073300	-3.19766900	0.85007200
C	-2.37669200	-2.17193800	0.93400600
C	-2.10853300	-1.41982800	-0.24555200
C	-2.75708000	-1.73316800	-1.44244800
C	-3.90977000	3.53949700	0.41923300
C	-3.58188200	2.78963700	1.53821500
C	-2.68771800	1.74668400	1.50056900
C	-2.08508600	1.42288000	0.28286900
C	-2.38334900	2.17628000	-0.88809900
C	-3.30183200	3.21324600	-0.77495400
F	-3.63183600	3.94828000	-1.84014700
F	-4.17111200	3.10445900	2.69420400
F	-4.29490800	-3.07145500	-2.58790500
F	-3.61368600	-3.93109100	1.92438200
C	0.80381600	-0.22101600	5.25552000
C	4.53019500	-3.64398600	-0.78251100
C	5.34235400	-3.04561500	-1.93564900
C	5.34005400	-3.55304500	0.51479500
C	4.24705900	-5.10935700	-1.08687300
C	4.52233000	3.65415600	0.73667800
C	5.80492700	2.85224200	0.55767900
C	4.49508900	4.78698700	-0.29393200
C	4.50160500	4.24790200	2.14899400
C	0.61803700	0.17412900	-5.31320900
H	-2.46151100	3.17543500	-3.52492400
H	0.50300000	-0.60887300	-2.70809000
H	-1.14706900	2.27694800	-5.39489400
H	-0.09890000	2.99997400	0.63795300
H	4.28911000	0.95514200	0.13704200
H	1.87993800	4.41294700	0.91100900
H	-0.12595500	-3.01222400	-0.67092700
H	1.83633700	-4.44065200	-0.95608700
H	4.27966500	-0.99631300	-0.22648200
H	-2.37362200	-3.17906700	3.56879300
H	-0.98178600	-2.30393400	5.39354800
H	0.60468600	0.57321300	2.65948800
H	-4.68187800	-4.32279100	-0.35641000
H	-2.56939900	-1.18503100	-2.35631800
H	-4.61859300	4.35627100	0.47445900
H	-2.47531700	1.19833000	2.40892100

H	1.45942100	-0.99256300	5.66769600
H	1.42173000	0.59401600	4.87751000
H	0.19365300	0.15903600	6.07929900
H	6.26613200	-3.61550500	-2.06333300
H	4.78137600	-3.08826000	-2.87287400
H	5.61215500	-2.00491800	-1.74402800
H	6.26718400	-4.12126500	0.40502600
H	4.78003300	-3.97057400	1.35552700
H	5.60381000	-2.52117700	0.75667600
H	3.68561500	-5.58969700	-0.28144600
H	3.68702000	-5.22884300	-2.01794200
H	5.19478100	-5.64096800	-1.19683800
H	6.66204500	3.51462600	0.69685000
H	5.88486800	2.04537100	1.29097000
H	5.87710400	2.41918400	-0.44346900
H	5.38022100	5.41538800	-0.16697700
H	3.61255800	5.41894200	-0.17547500
H	4.50014700	4.39019300	-1.31246400
H	4.51210200	3.45880000	2.90542400
H	5.38680200	4.87234500	2.29375500
H	3.61875200	4.86937800	2.31281000
H	1.30903700	0.92169900	-5.71224900
H	1.19744000	-0.68257400	-4.96763900
H	-0.02198100	-0.14800100	-6.13867200

6. X-ray Crystallographic Analysis

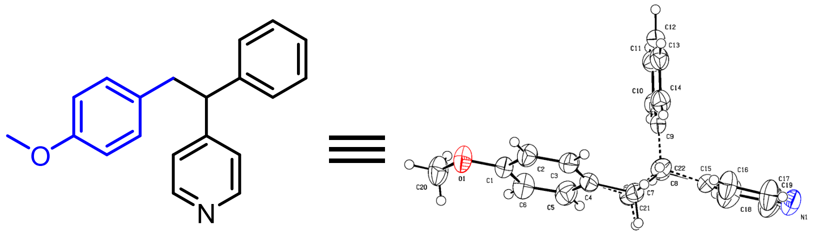

5c **CCDC:2556683**

Table S6. Crystal data and structure refinement for **5c**

Identification code	5c
Empirical formula	$C_{20}H_{19}NO$
Formula weight	289.36
Temperature/K	170.00
Crystal system	monoclinic
Space group	$P2_1/c$
a/Å	15.0927(5)

b/Å	12.6712(4)
c/Å	8.3916(3)
α/°	90
β/°	101.272(2)
γ/°	90
Volume/Å^3	1573.87(9)
Z	4
ρ_{calc}g/cm^3	1.221
μ/mm^{-1}	0.581
F(000)	616.0
Crystal size/mm^3	0.15 × 0.12 × 0.1
Radiation	CuKα (λ = 1.54178)
2Θ range for data collection/°	5.97 to 136.886
Index ranges	-18 ≤ h ≤ 18, -15 ≤ k ≤ 15, -9 ≤ l ≤ 9
Reflections collected	12821
Independent reflections	2860 [R_{int} = 0.0641, R_{sigma} = 0.0720]
Data/restraints/parameters	2860/57/219
Goodness-of-fit on F^2	1.038
Final R indexes [I>=2σ (I)]	R_1 = 0.0494, wR_2 = 0.1256
Final R indexes [all data]	R_1 = 0.0997, wR_2 = 0.1331
Largest diff. peak/hole / e Å^{-3}	0.24/-0.28

7. References

(1) Frisch, M. J.; Trucks, G. W.; Schlegel, H. B.; Scuseria, G. E.; Robb, M. A.; Cheeseman, J. R.; Scalmani, G.; Barone, V.; Mennucci, B.; Petersson, G. A.; Nakatsuji, H.; Caricato, M.; Li, X.; Hratchian, H. P.; Izmaylov, A. F.; Bloino, J.; Zheng, G.; Sonnenberg, J. L.; Hada, M.; Ehara, M.; Toyota, K.; Fukuda, R.; Hasegawa, J.; Ishida, M.; Nakajima, T.; Honda, Y.; Kitao, O.; Nakai, H.; Vreven, T.; Montgomery, J. A., Jr.; Peralta, J. E.; Ogliaro, F.; Bearpark, M.; Heyd, J. J.; Brothers, E.; Kudin, K. N.; Staroverov, V. N.; Kobayashi, R.; Normand, J.; Raghavachari, K.; Rendell, A.; Burant, J. C.; Iyengar, S. S.; Tomasi, J.; Cossi, M.; Rega, N.; Millam, N. J.; Klene, M.; Knox, J. E.; Cross, J. B.; Bakken, V.; Adamo, C.; Jaramillo, J.; Gomperts, R.; Stratmann, R. E.; Yazyev, O.; Austin, A. J.; Cammi, R.; Pomelli, C.; Ochterski, J. W.; Martin, R. L.; Morokuma, K.; Zakrzewski, V. G.; Voth, G. A.; Salvador, P.; Dannenberg, J. J.; Dapprich, S.; Daniels, A. D.; Farkas, Ö.; Foresman, J. B.; Ortiz, J. V.; Cioslowski, J., & Fox, D. J. Gaussian, Inc., Gaussian 09, Revision E.01. *Wallingford CT* **2013**.

(2) Adamo, C.; Barone, V. *J. Chem. Phys.* **1999**, *110*, 6158-6170.

(3) Grimme, S.; Antony, J.; Ehrlich, S.; Krieg, H. *J. Chem. Phys.* **2010**, *132*, 154104.

(4) Weigend, F.; Ahlrichs, R. *Phys. Chem. Chem. Phys.* **2005**, *7*, 3297-3305. S39

(5) Marenich, A. V.; Cramer, C. J.; Truhlar, D. G. *J. Phys. Chem. B* **2009**, *113*, 6378-6396.

8. ^{1}H NMR, ^{13}C NMR and ^{19}F NMR Spectra

^{1}H NMR-spectrum (400MHz, $CDCl_3$) of 4a

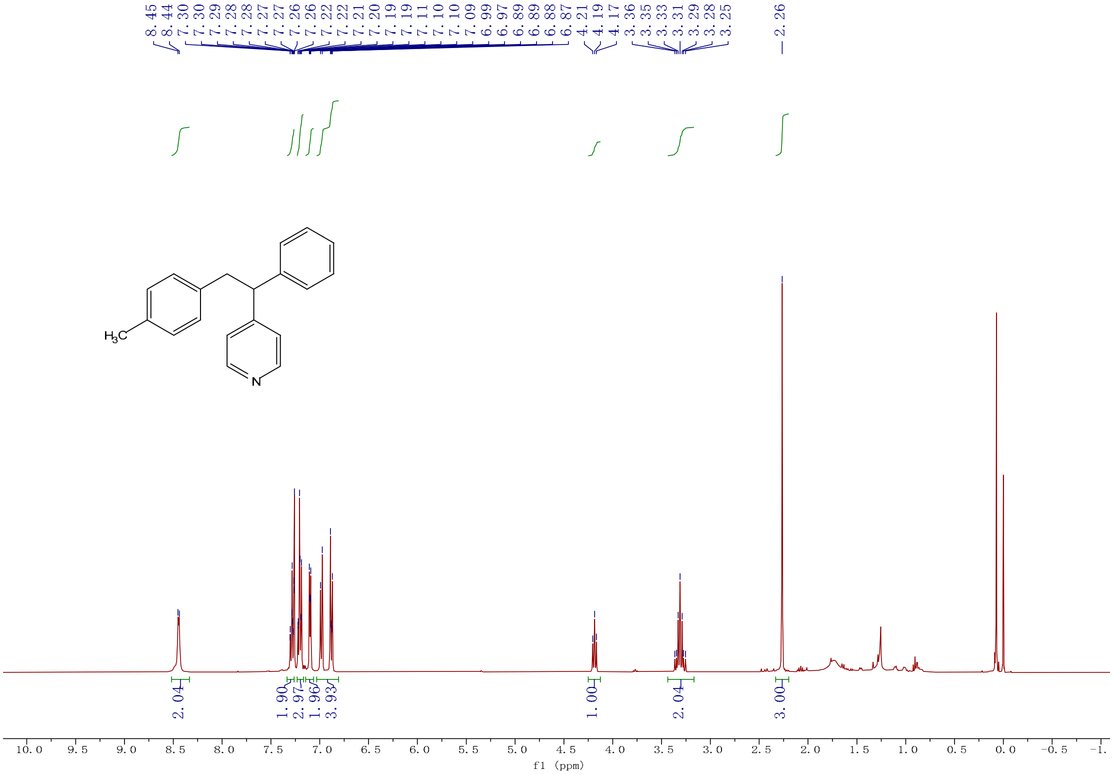

^{13}C{^{1}H} NMR -spectrum (100MHz, $CDCl_3$) of 4a

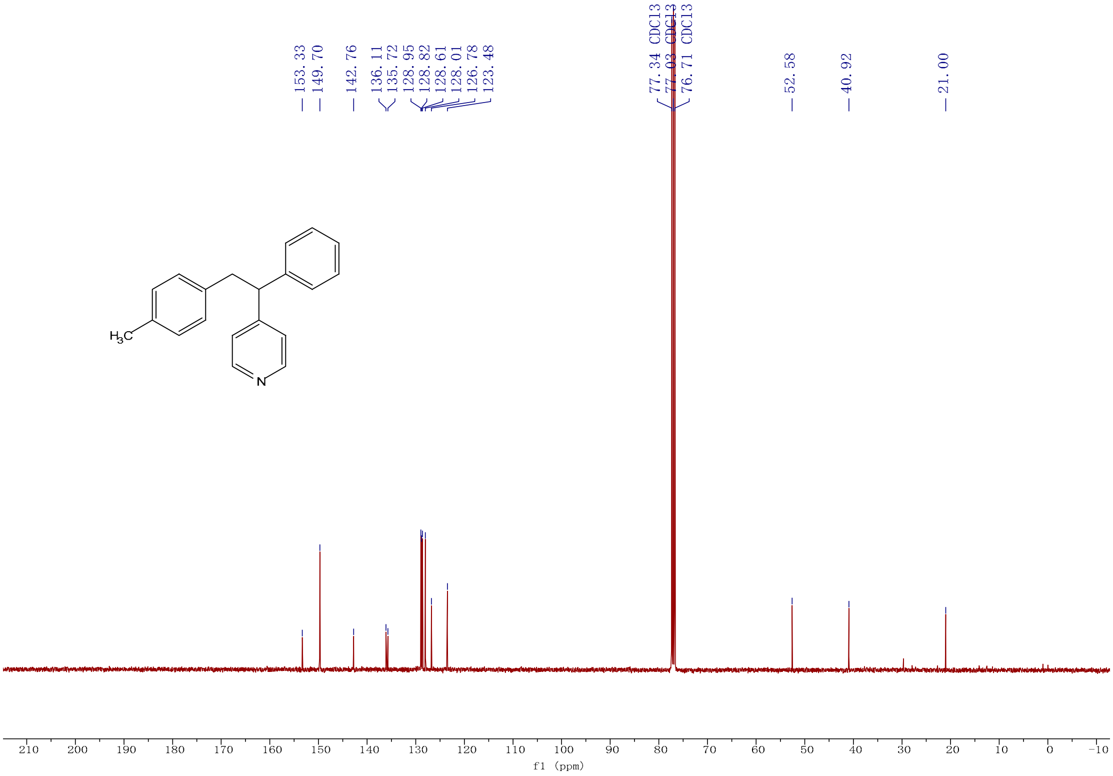

^{1}H NMR-spectrum (400MHz, $CDCl_3$) of 4b

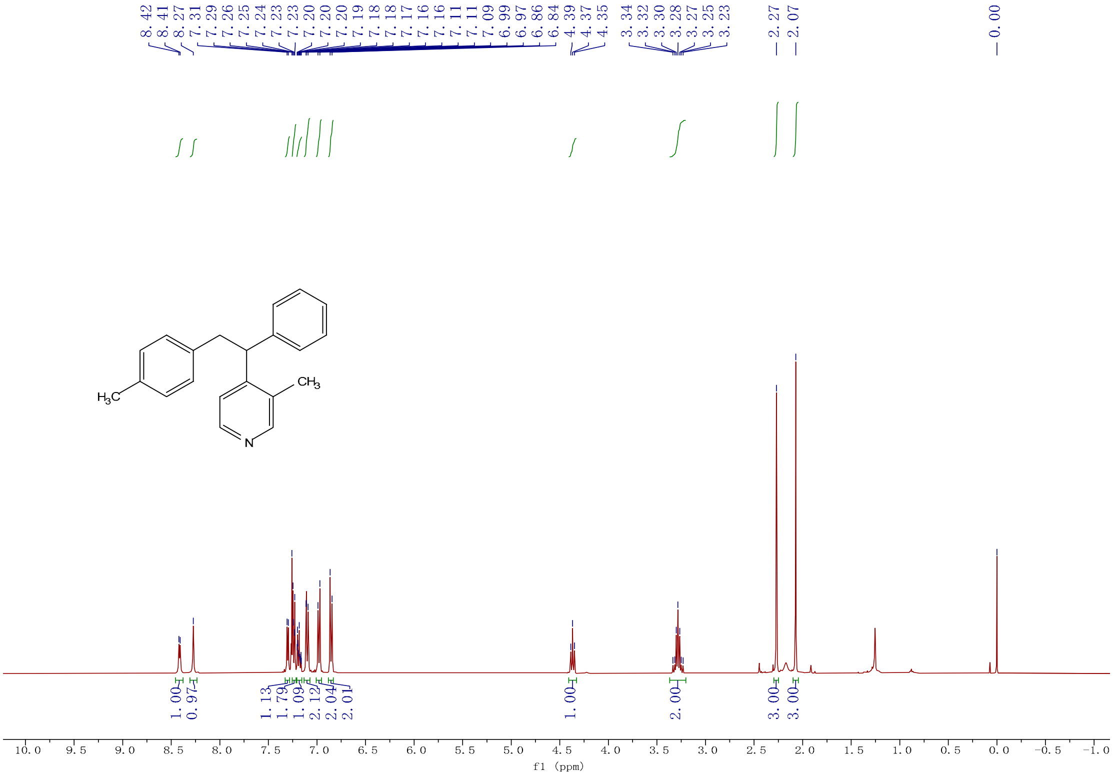

^{13}C{^{1}H} NMR -spectrum (100MHz, $CDCl_3$) of 4b

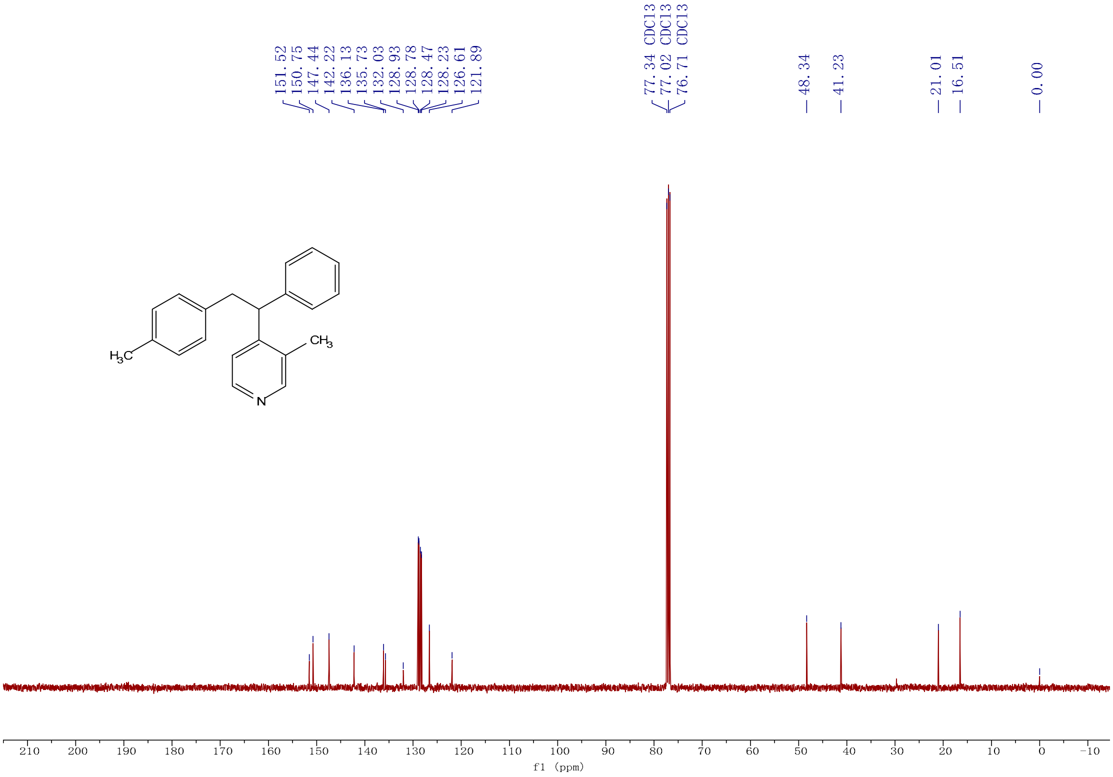

^{1}H NMR-spectrum (400MHz, $CDCl_3$) of 4c

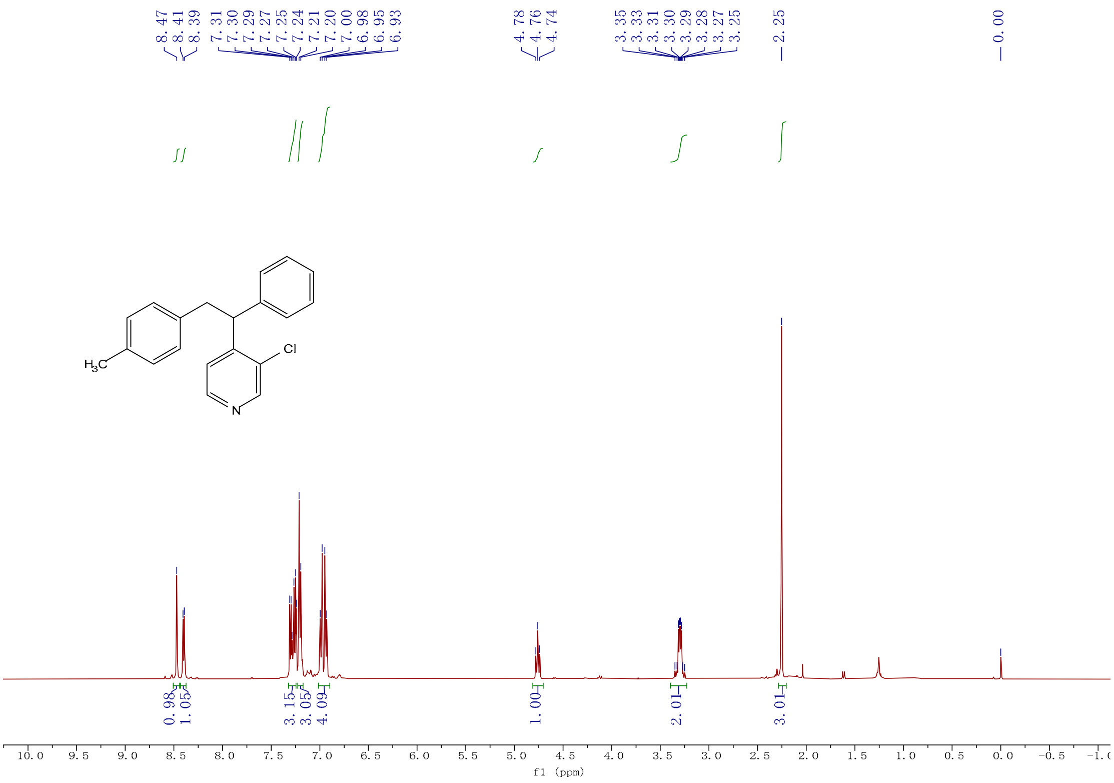

$^{13}C\{^{1}H\}$ NMR -spectrum (100MHz, $CDCl_3$) of 4c

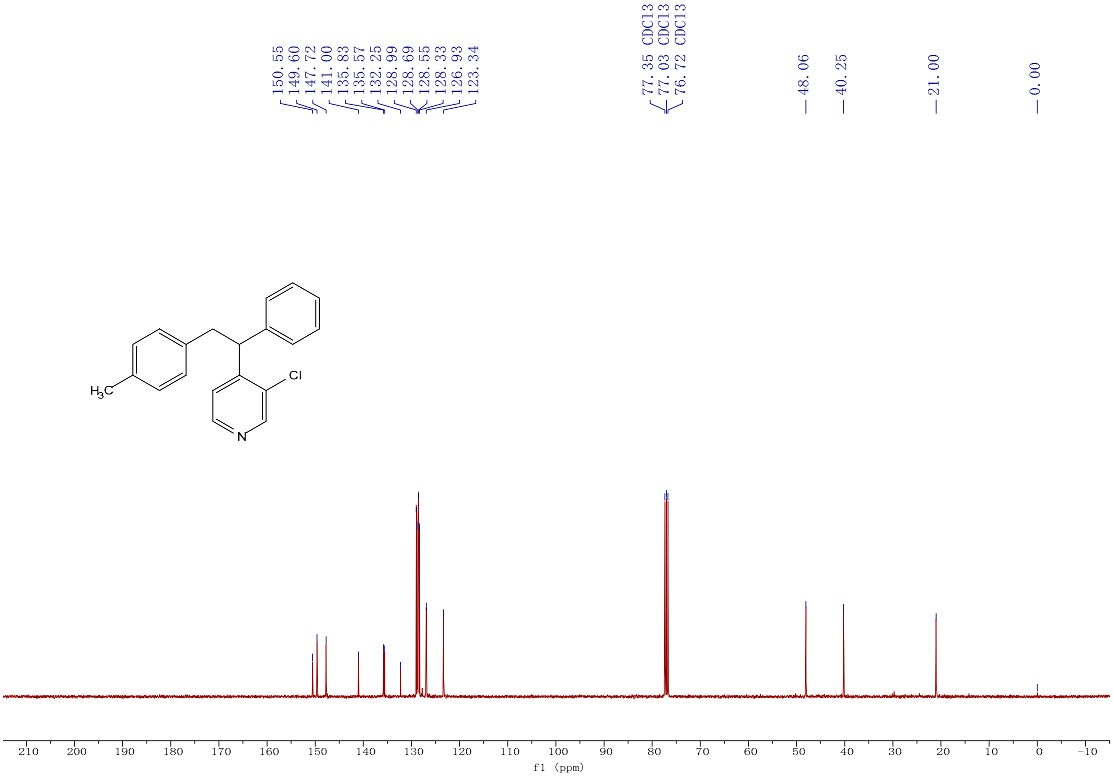

^{1}H NMR-spectrum (400MHz, $CDCl_3$) of 4d

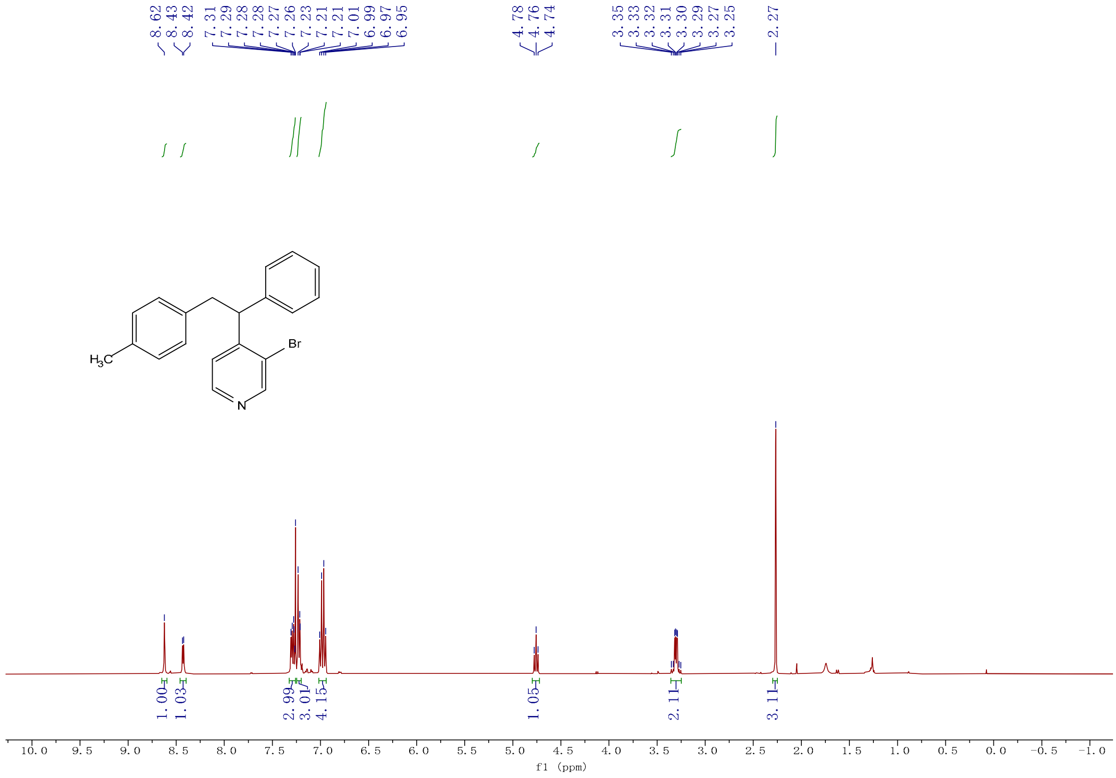

$^{13}C\{^{1}H\}$ NMR -spectrum (100MHz, $CDCl_3$) of 4d

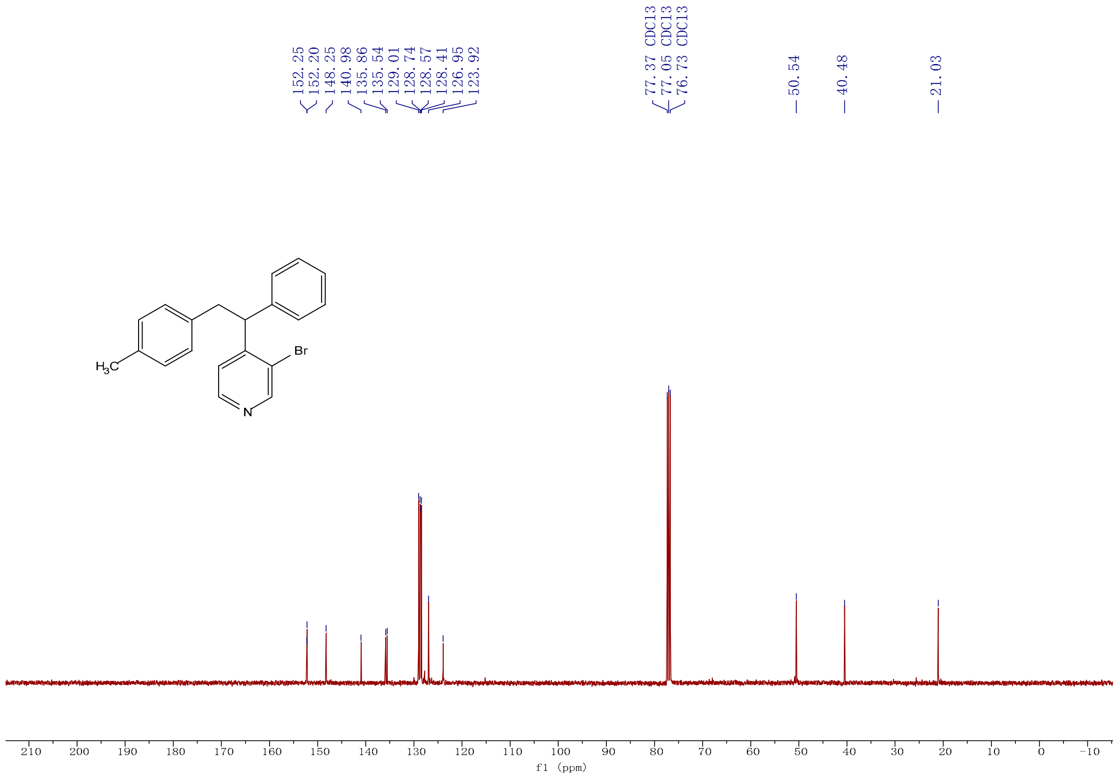

^{1}H NMR-spectrum (400MHz, $CDCl_3$) of 4e

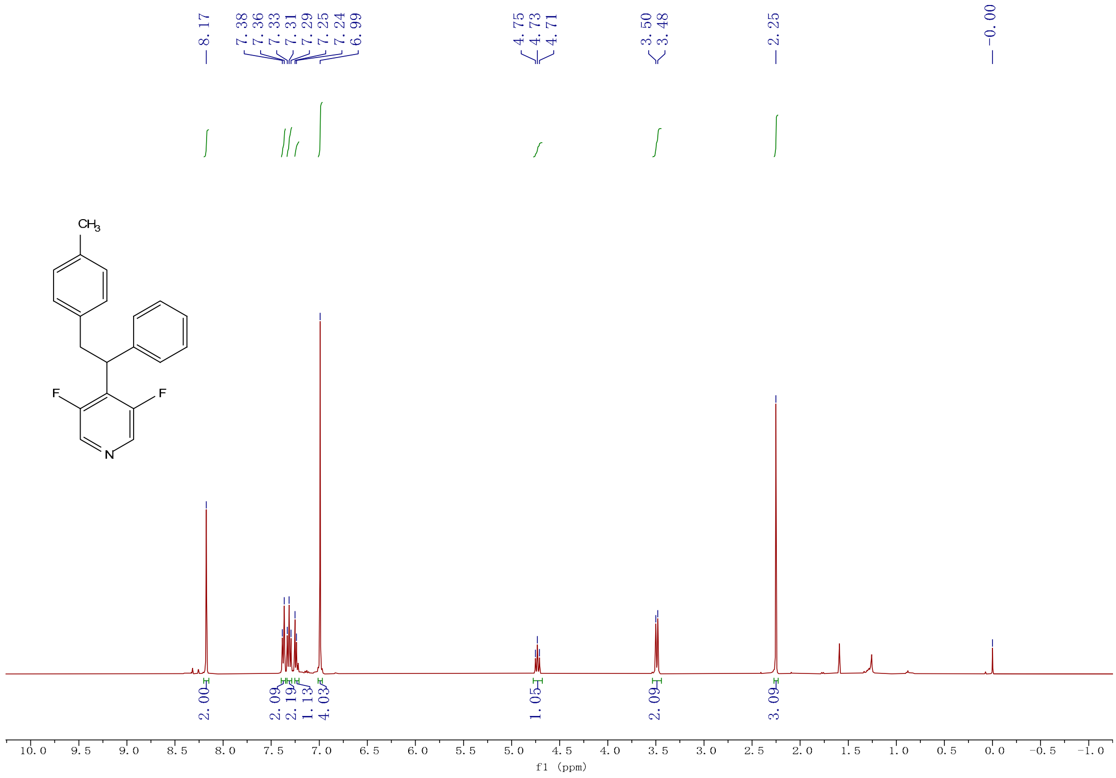

^{13}C{^{1}H} NMR-spectrum (100MHz, $CDCl_3$) of 4e

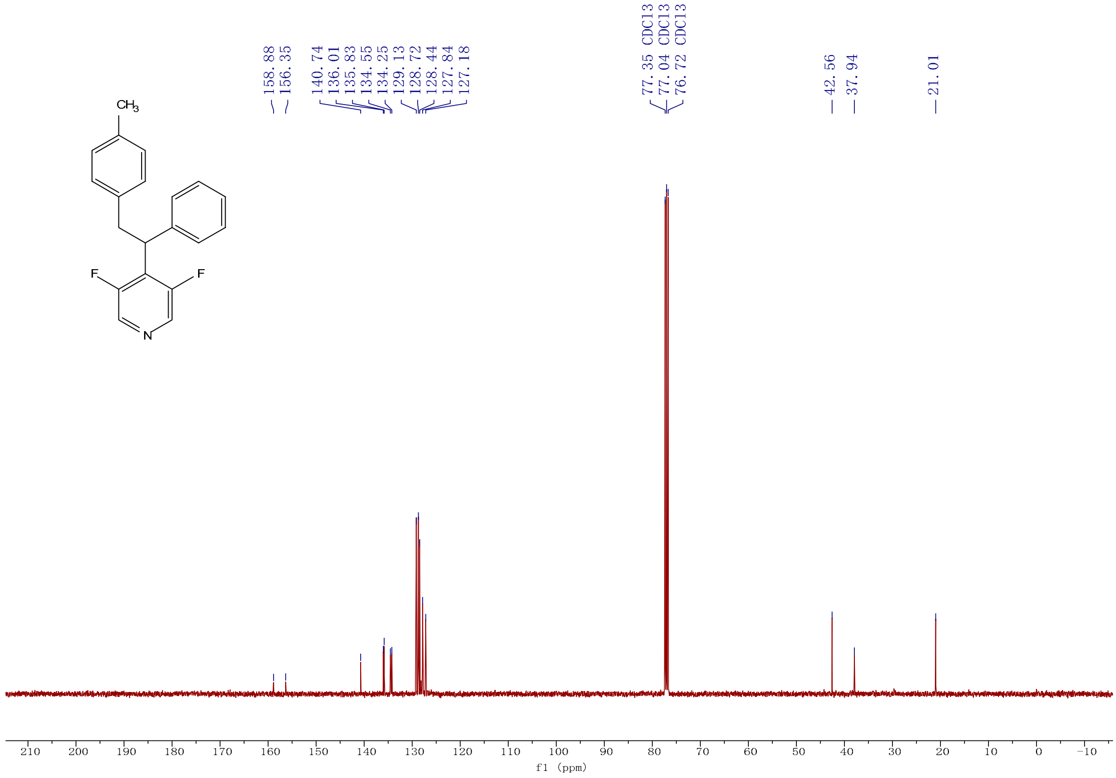

^{19}F NMR-spectrum (376 MHz, CDCl$_3$) of 4e

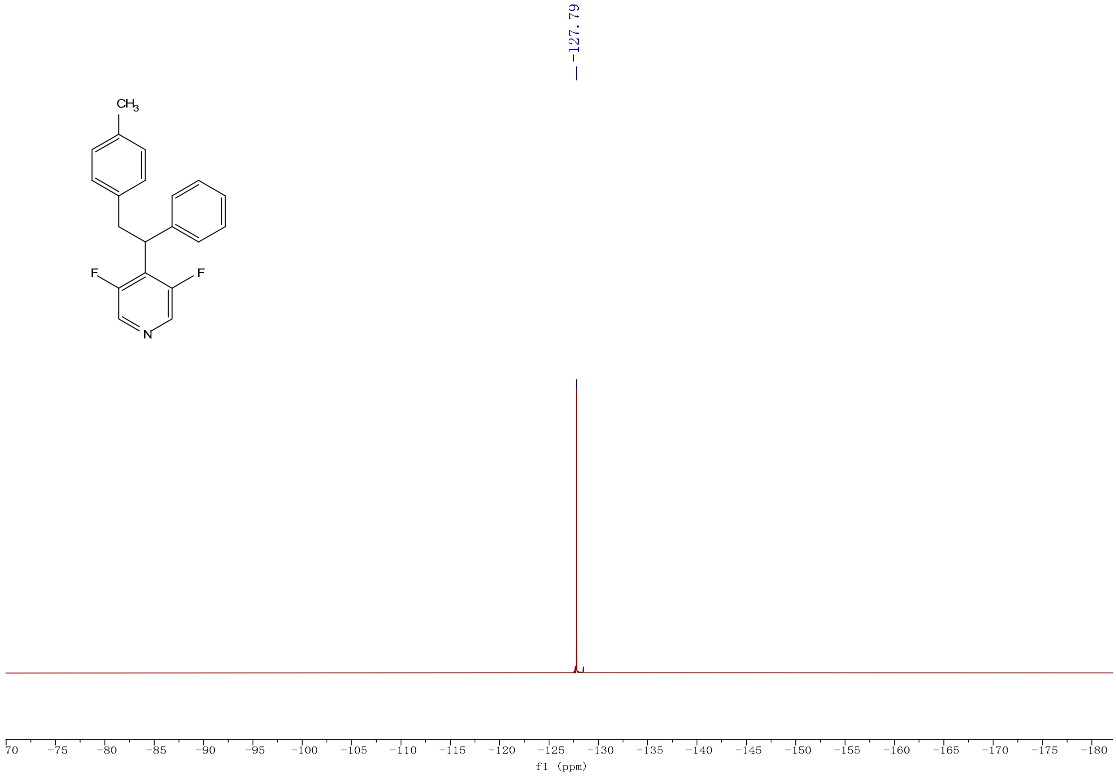

^{1}H NMR-spectrum (400MHz, CDCl$_3$) of 5a

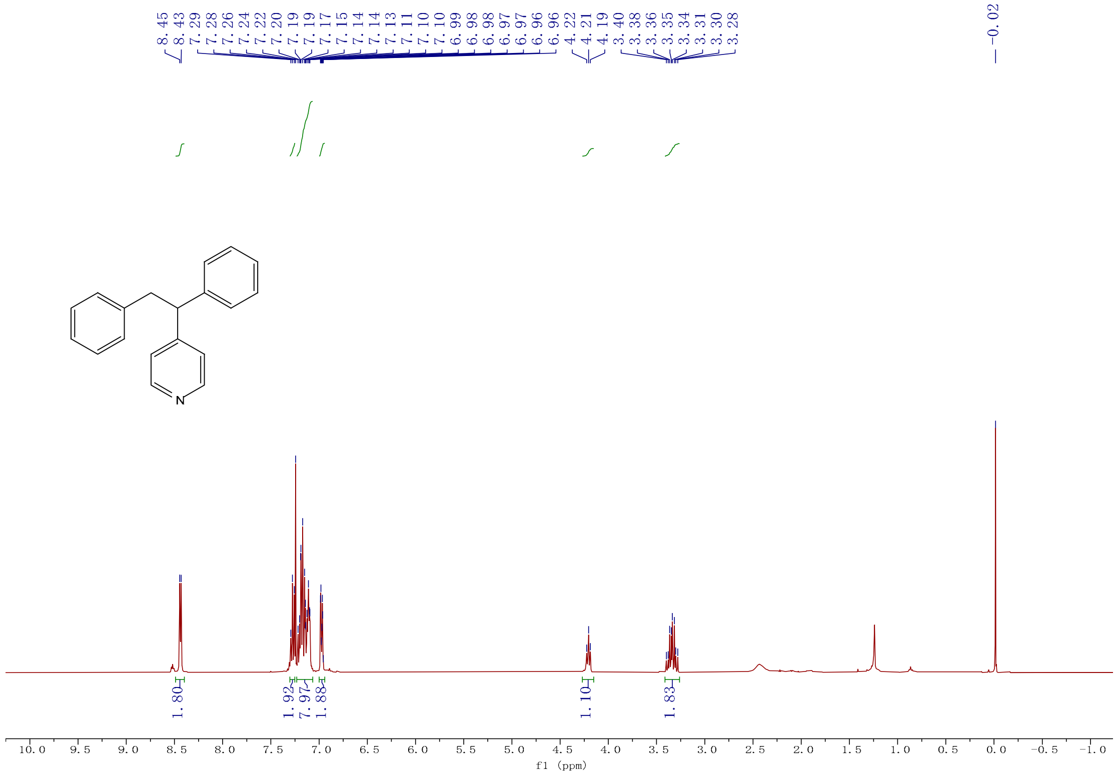

$^{13}C\{^{1}H\}$ NMR -spectrum (**100**MHz, $CDCl_3$) of **5a**

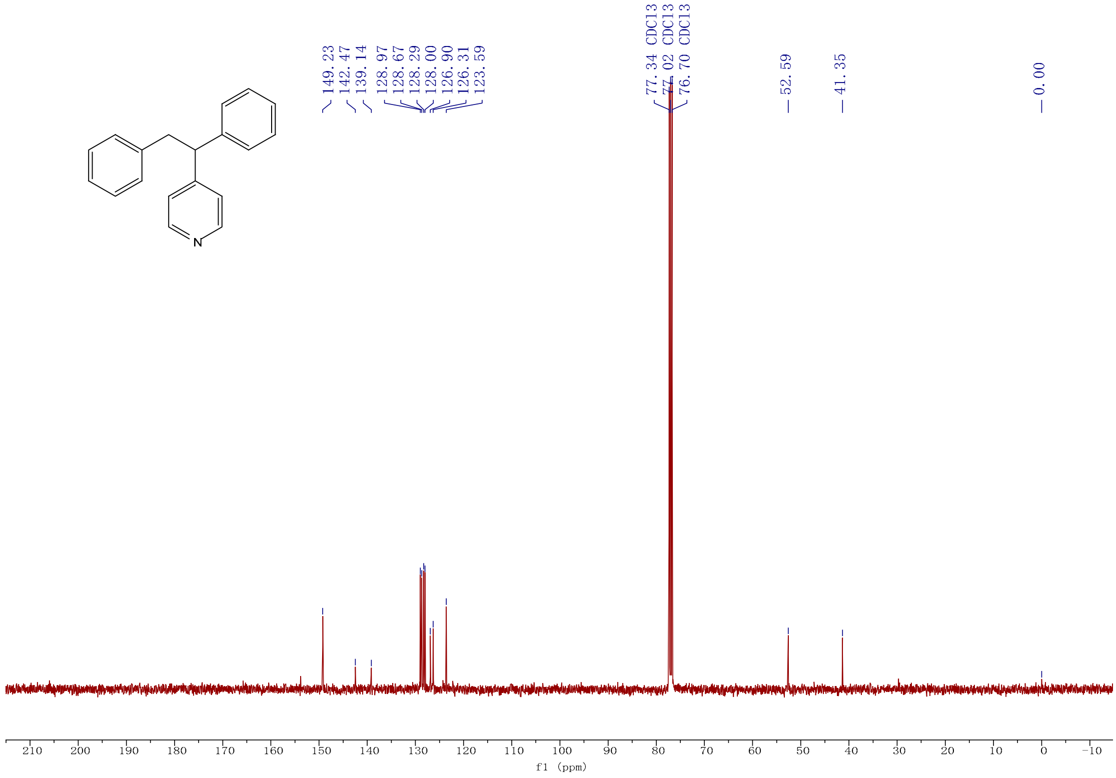

^{1}H NMR-spectrum (400MHz, $CDCl_3$) of **5b**

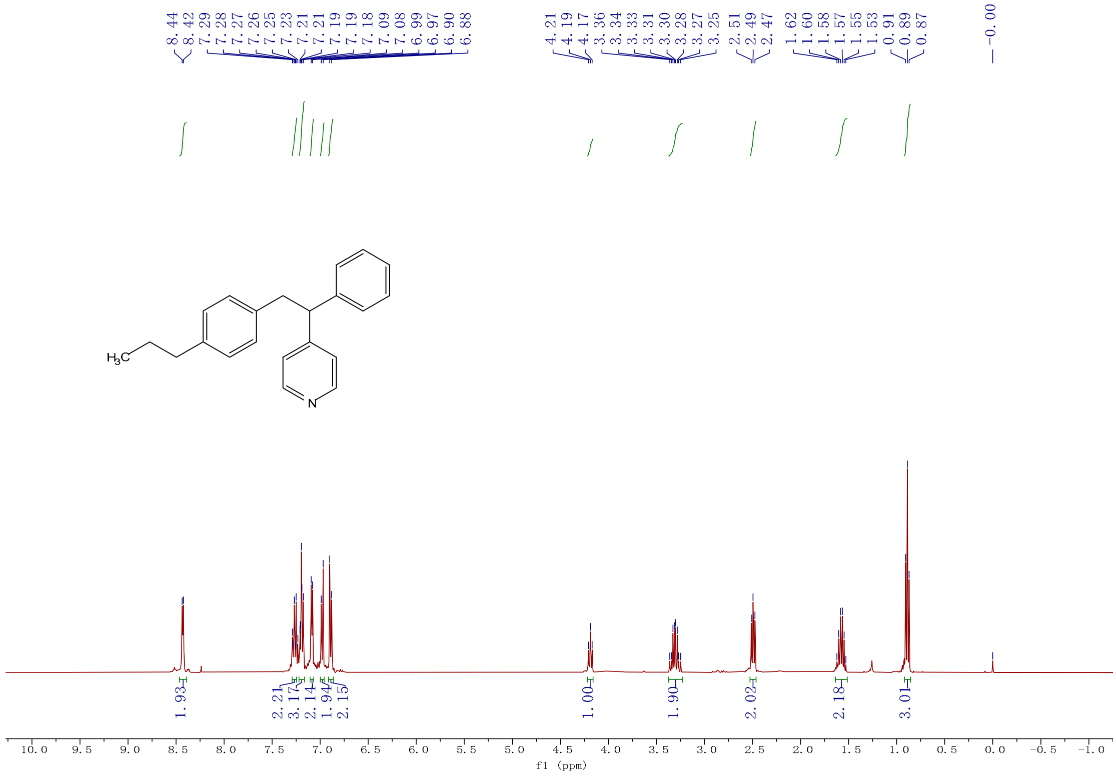

$^{13}C\{^{1}H\}$ **NMR** -spectrum (**100**MHz, $CDCl_3$) of **5b**

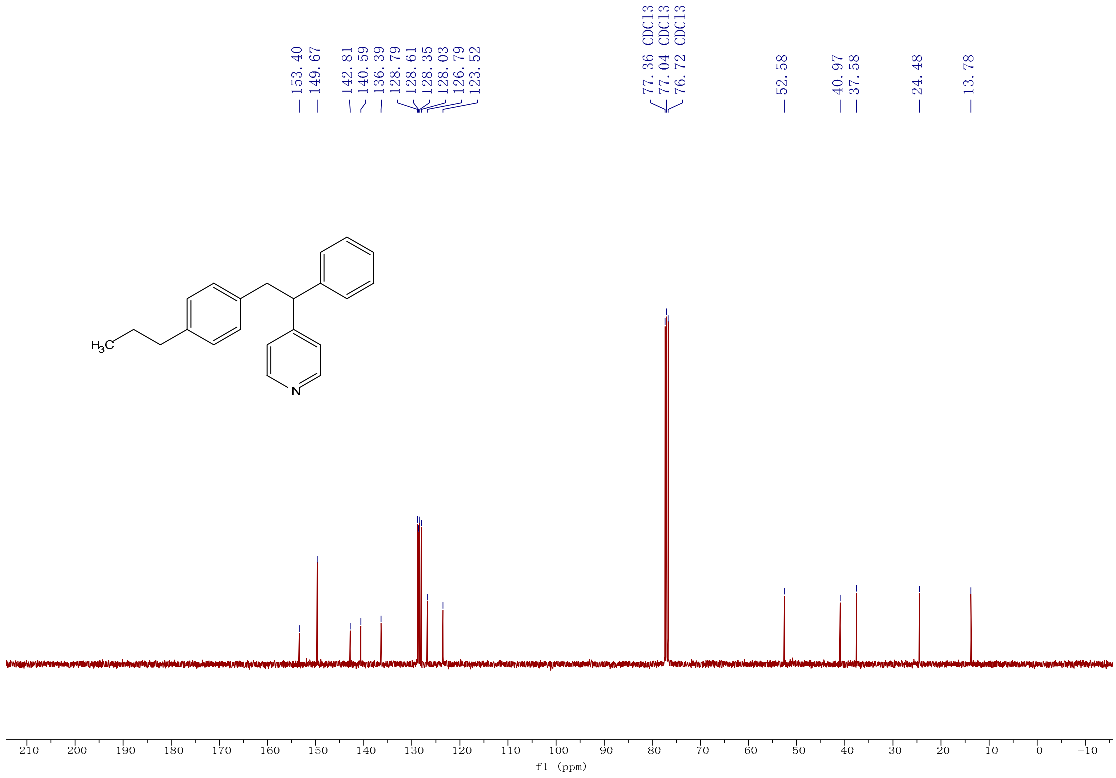

^{1}**H NMR**-spectrum (400MHz, $CDCl_3$) of **5c**

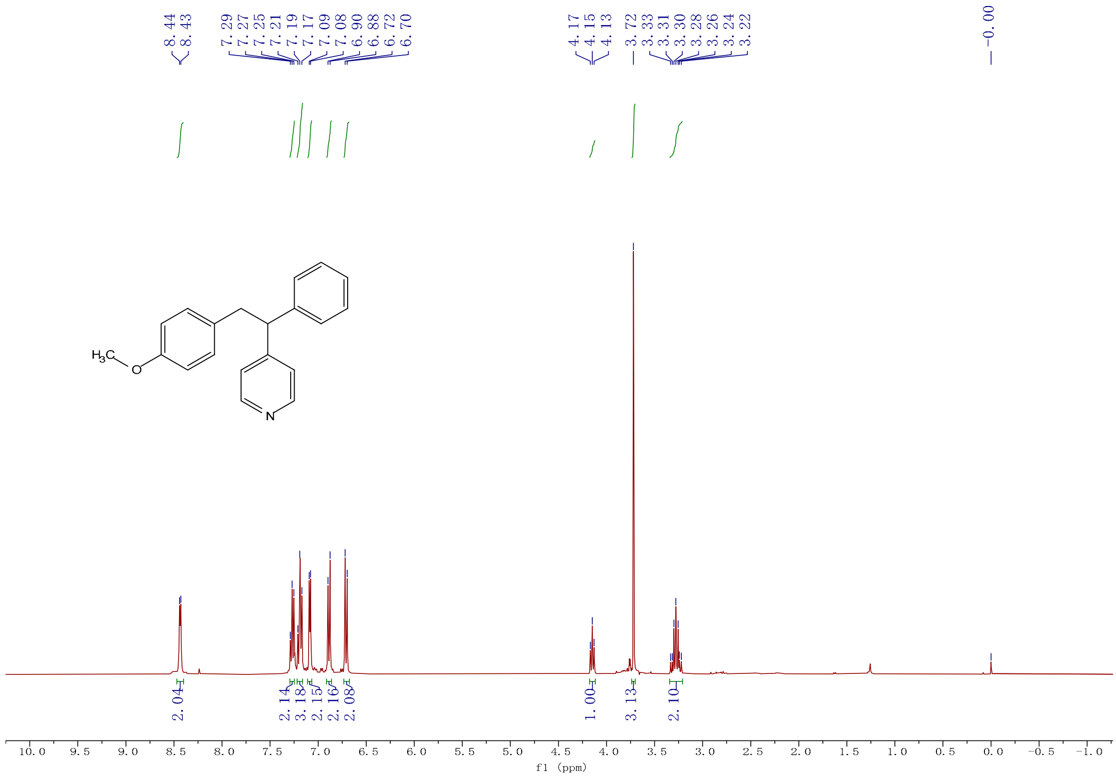

^{13}C{^{1}H} **NMR** -spectrum (**100**MHz, $CDCl_3$) of **5c**

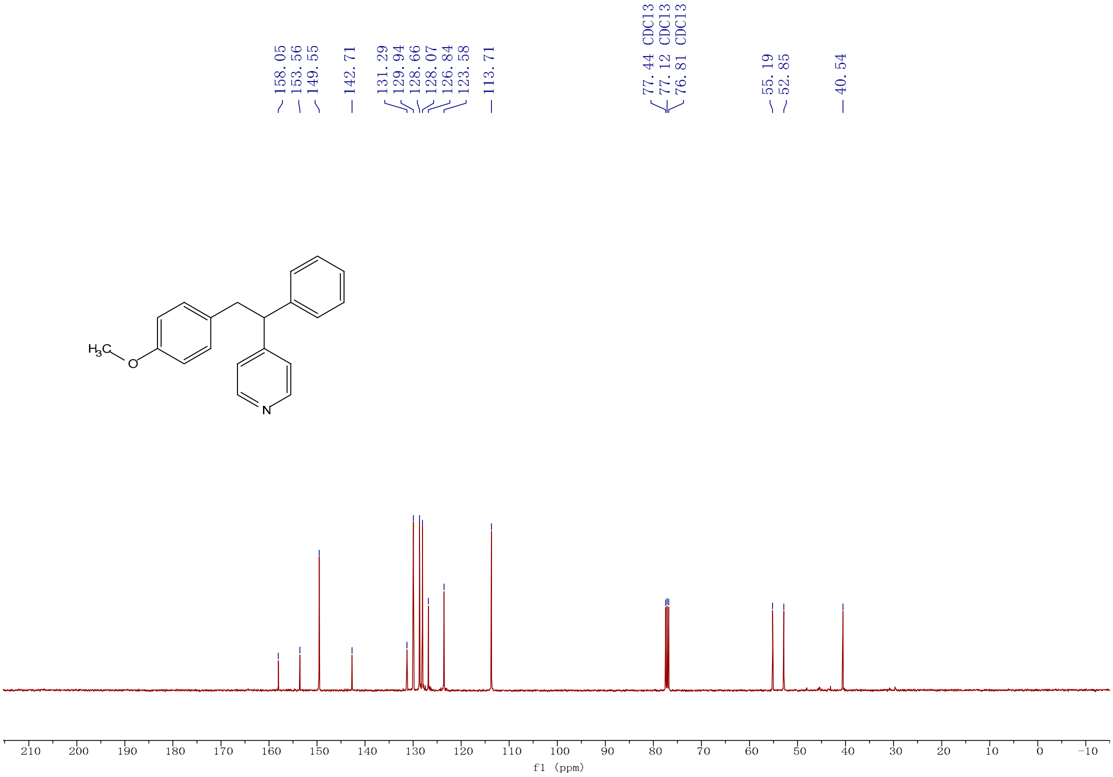

[1]**H NMR**-spectrum (400MHz, $CDCl_3$) of **5d**

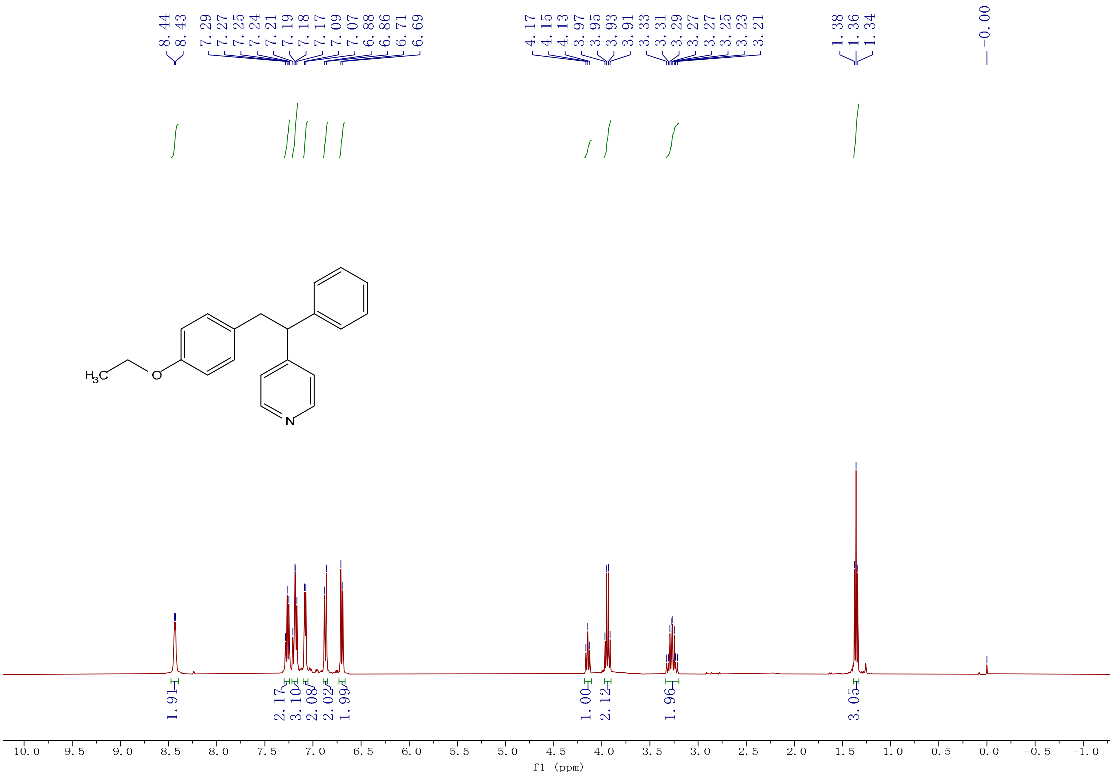

$^{13}C\{^{1}H\}$ NMR -spectrum (**100**MHz, $CDCl_3$) of **5d**

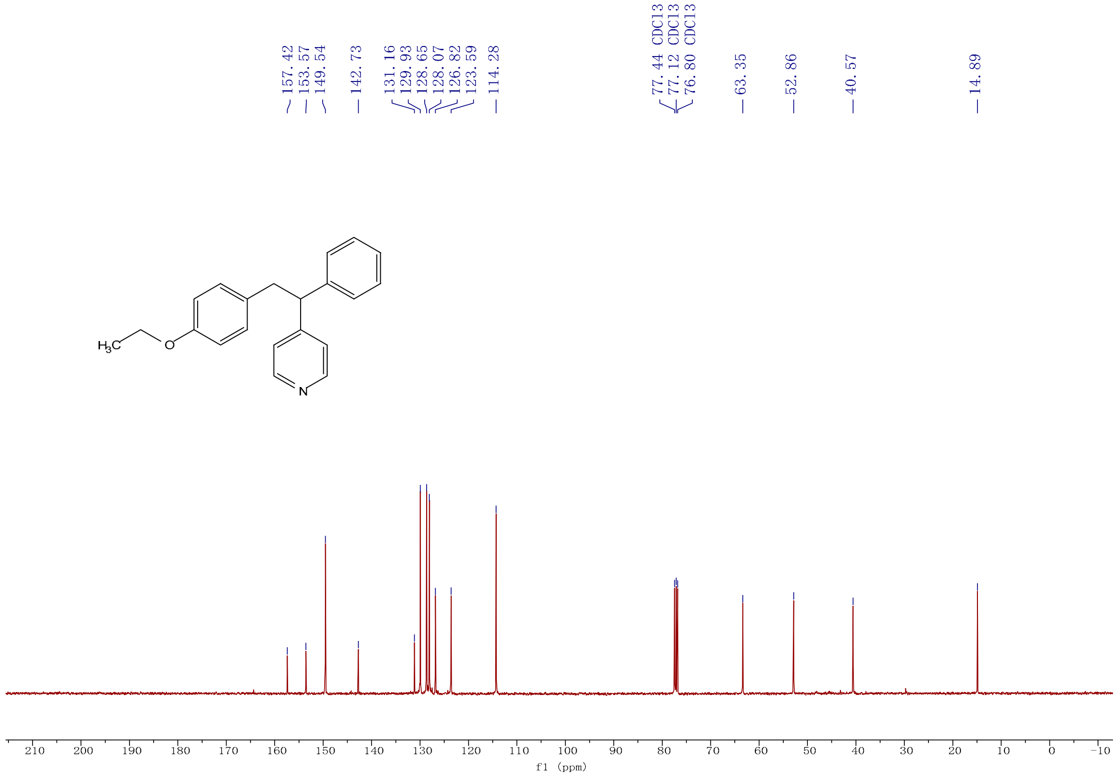

^{1}H NMR-spectrum (400MHz, $CDCl_3$) of **5e**

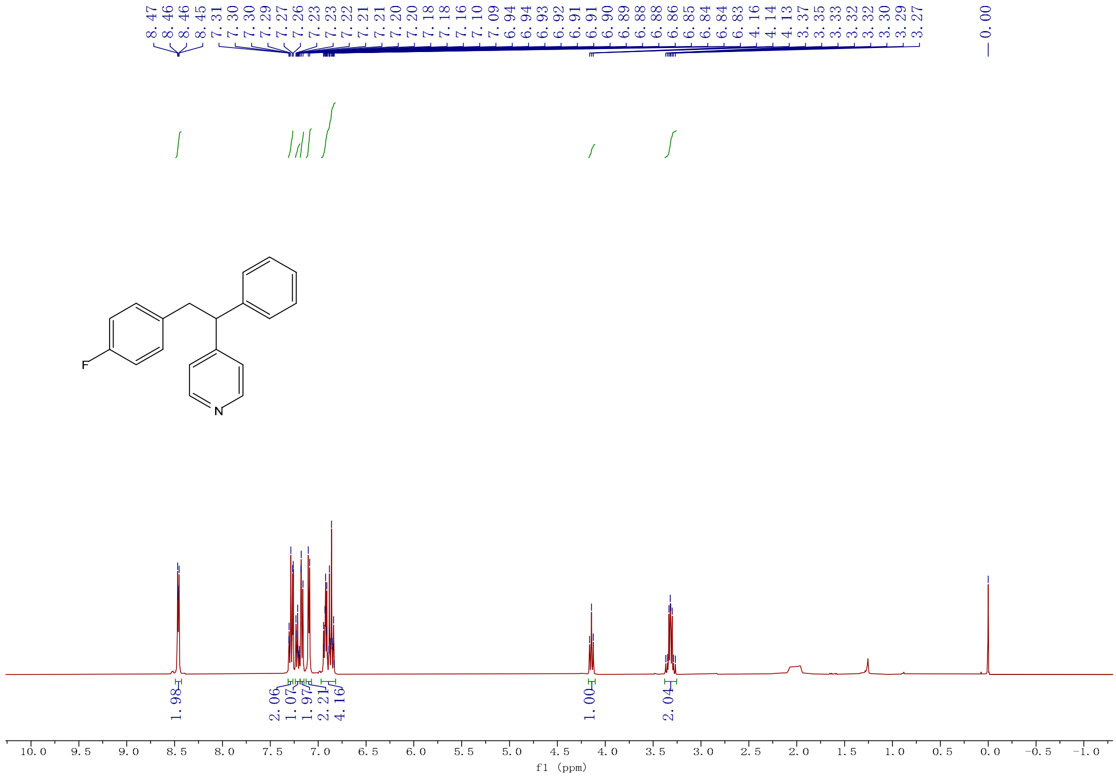

$^{13}C\{^{1}H\}$ NMR -spectrum (**100**MHz, $CDCl_3$) of **5e**

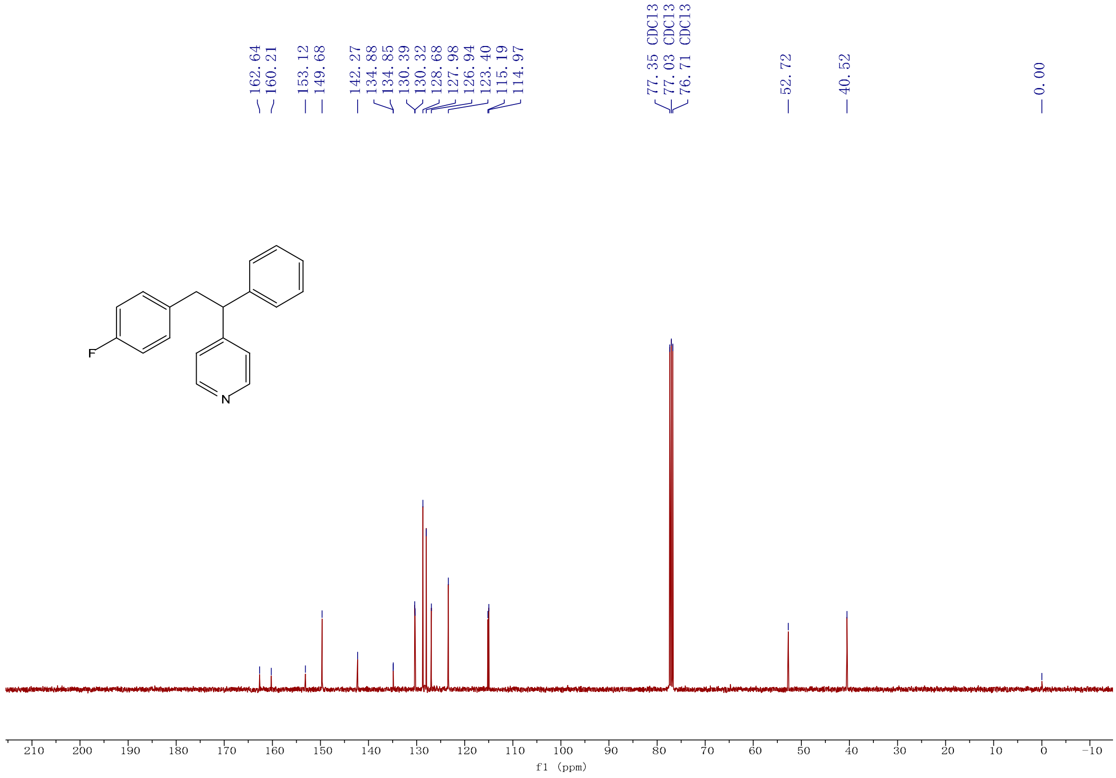

^{19}F NMR-spectrum (376 MHz, $CDCl_3$) of **5e**

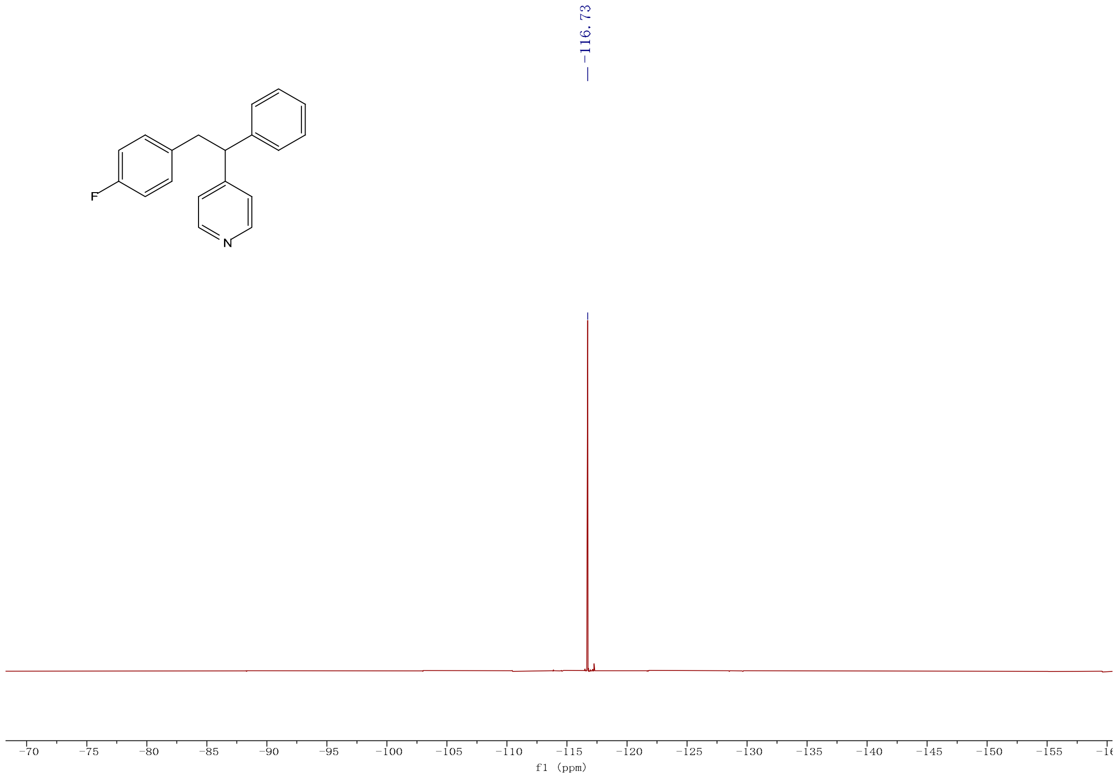

^{1}H NMR-spectrum (400MHz, $CDCl_3$) of **5f**

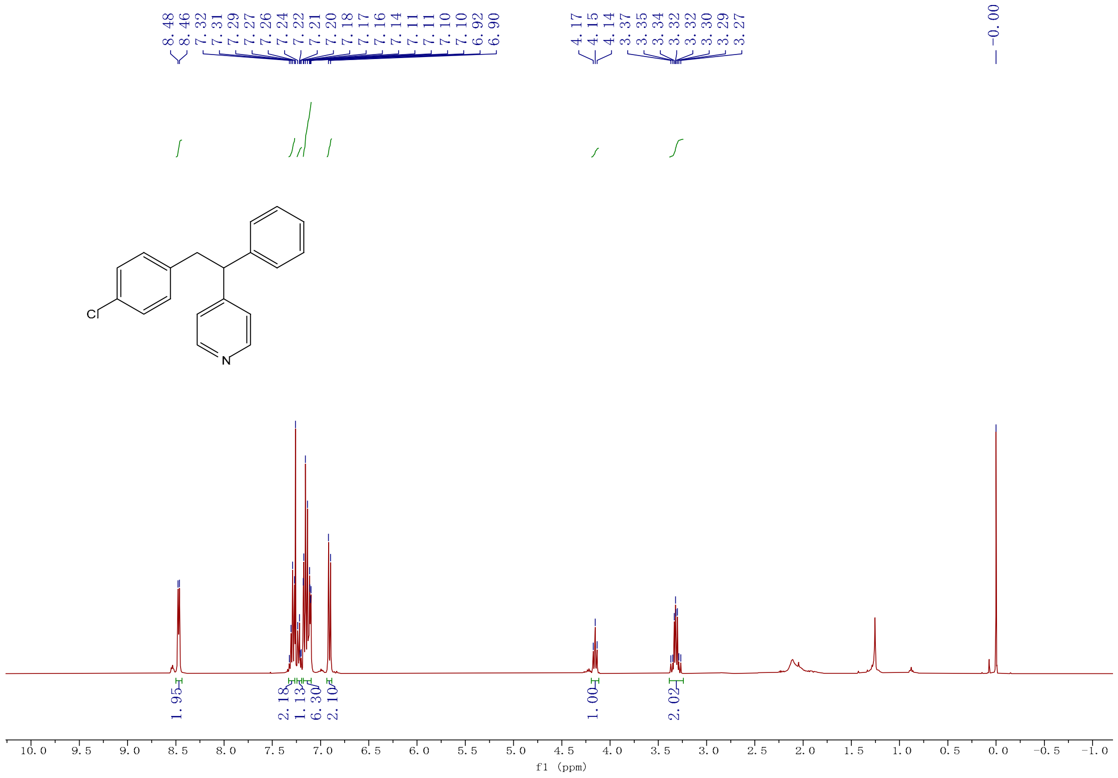

^{13}C{^{1}H} NMR -spectrum (**100**MHz, $CDCl_3$) of **5f**

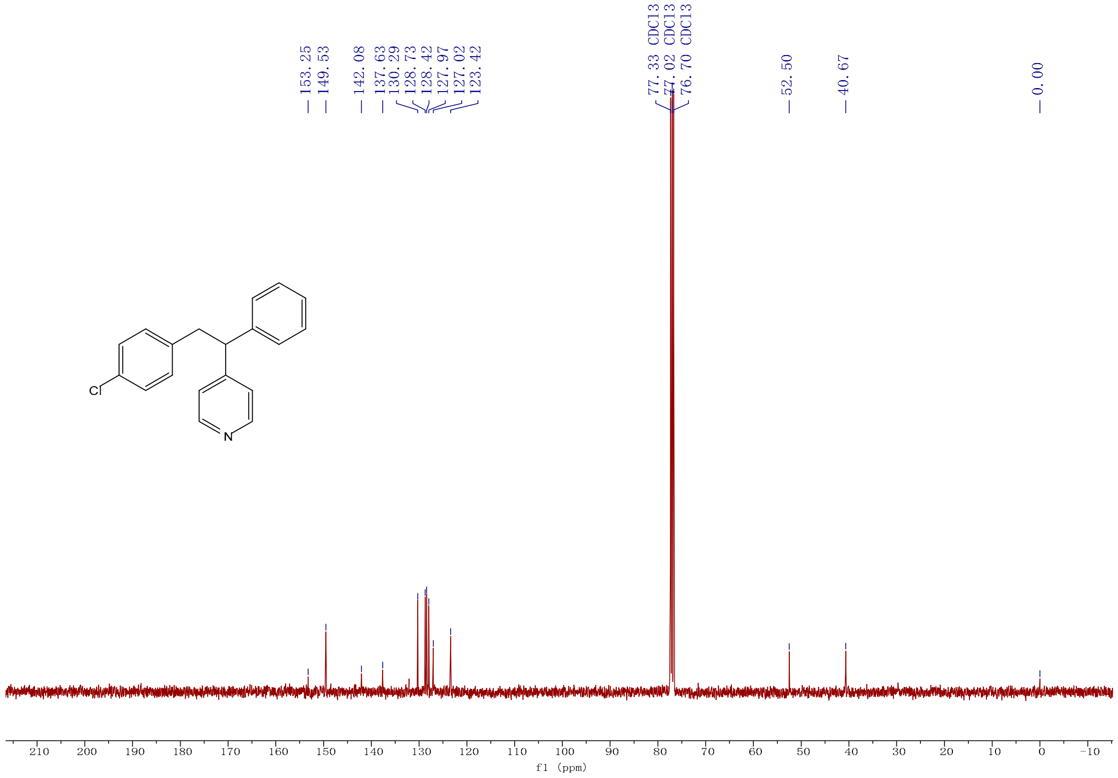

^{1}H NMR-spectrum (400MHz, $CDCl_3$) of **5g**

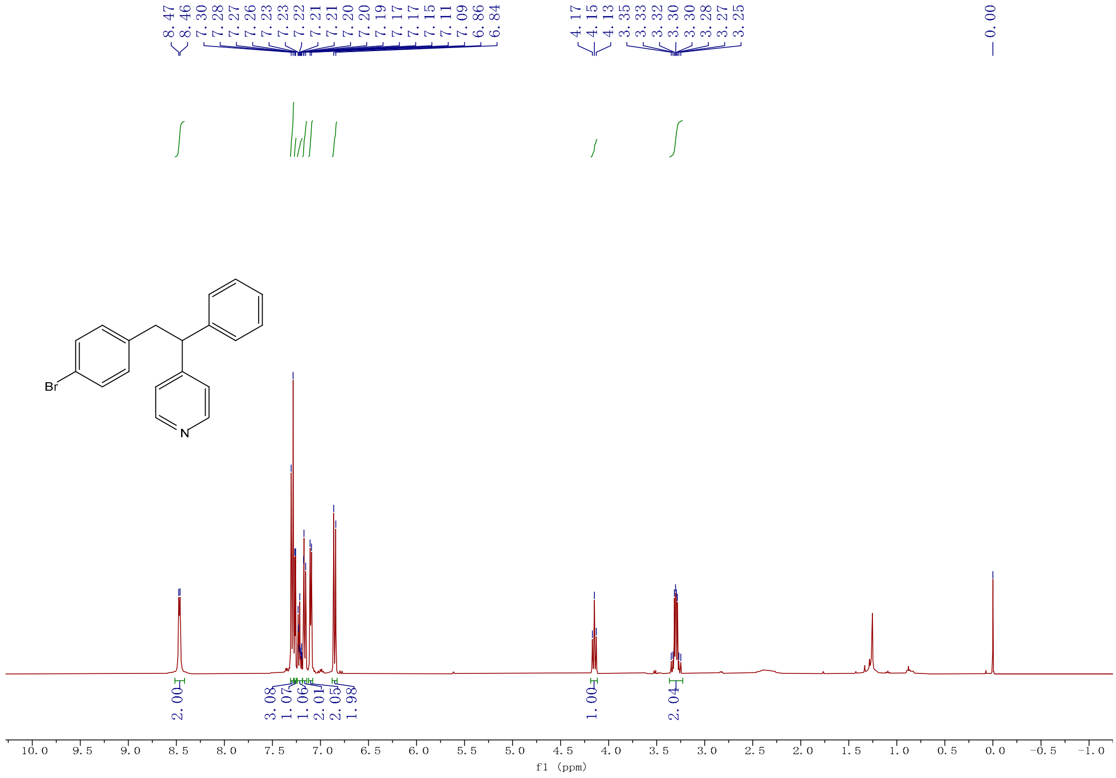

^{13}C{^{1}H} NMR -spectrum (**100**MHz, $CDCl_3$) of **5g**

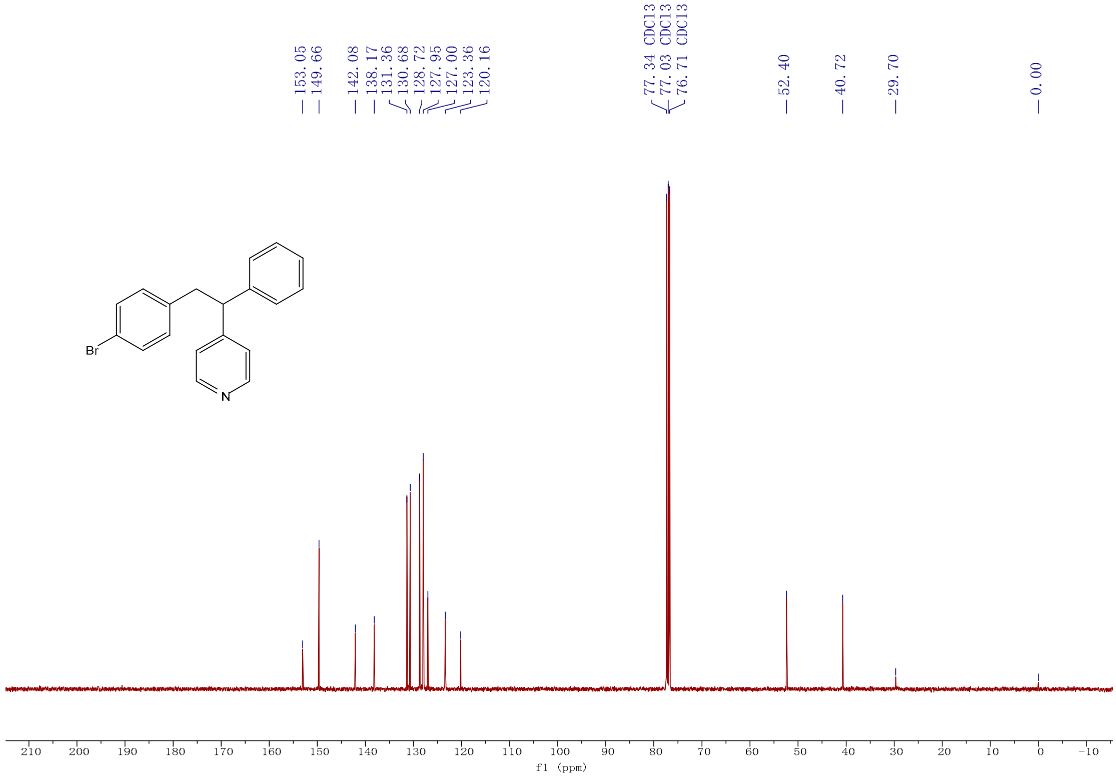

^{1}H NMR-spectrum (400MHz, $CDCl_3$) of **5h**

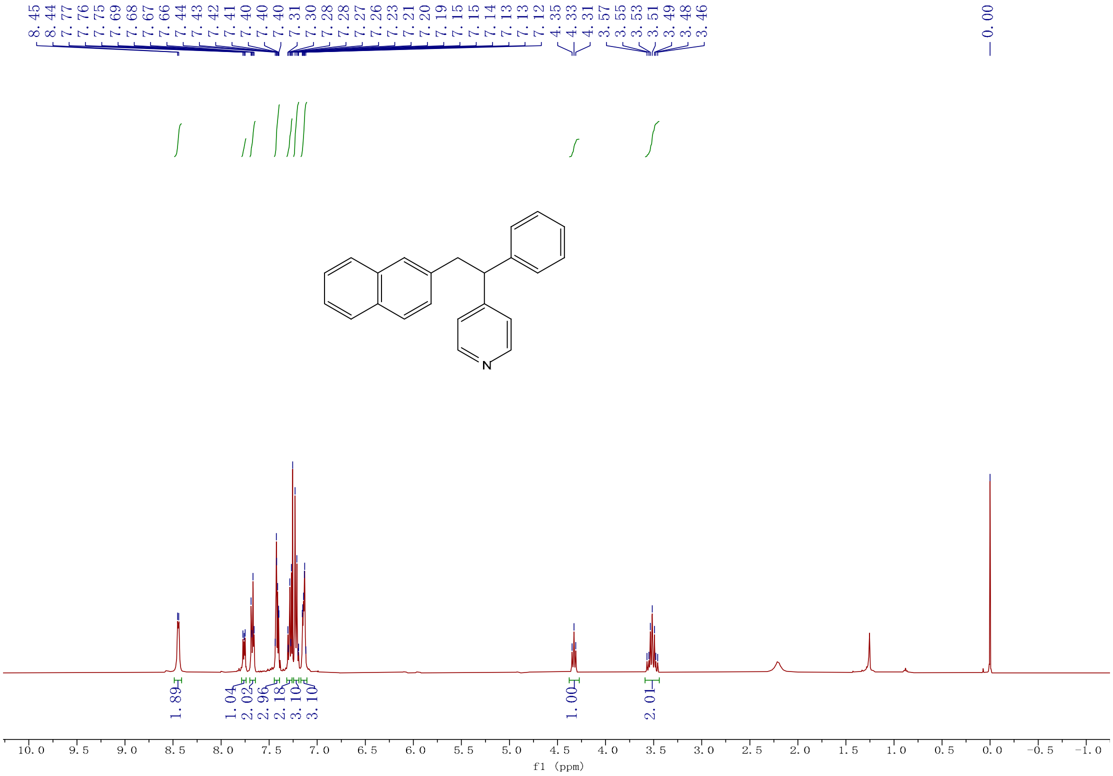

^{13}C{^{1}H} NMR -spectrum (**100**MHz, $CDCl_3$) of **5h**

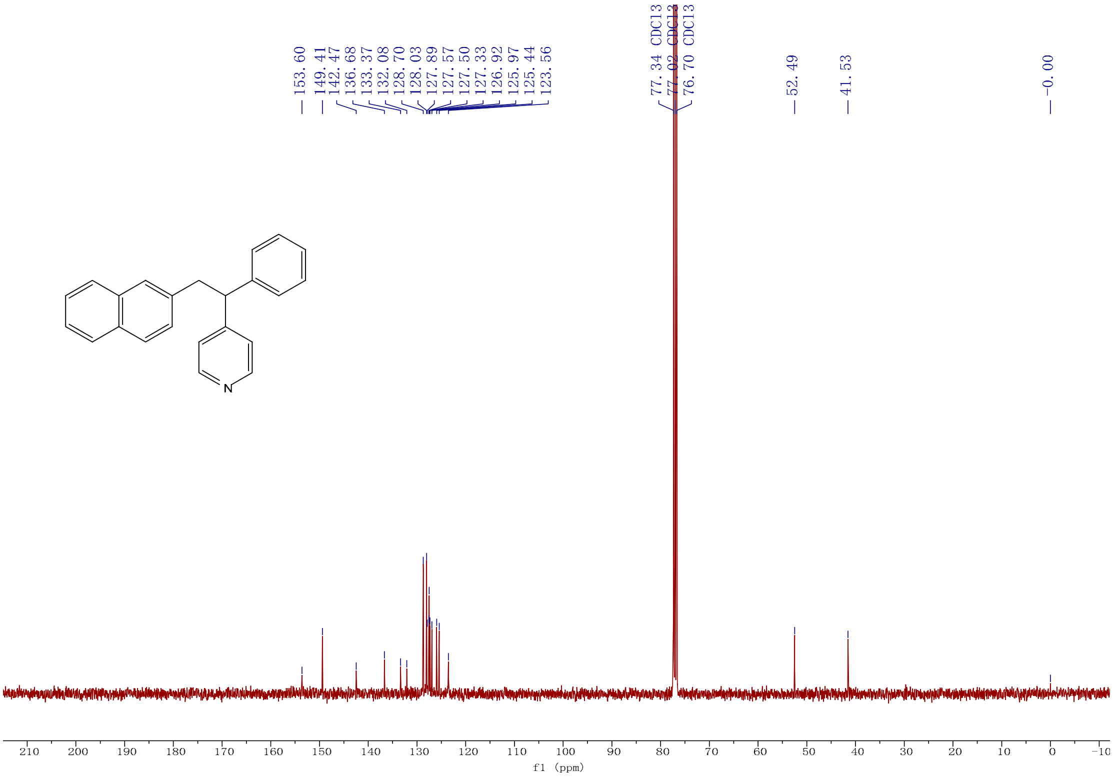

^{1}**H NMR**-spectrum (400MHz, $CDCl_3$) of **5i**

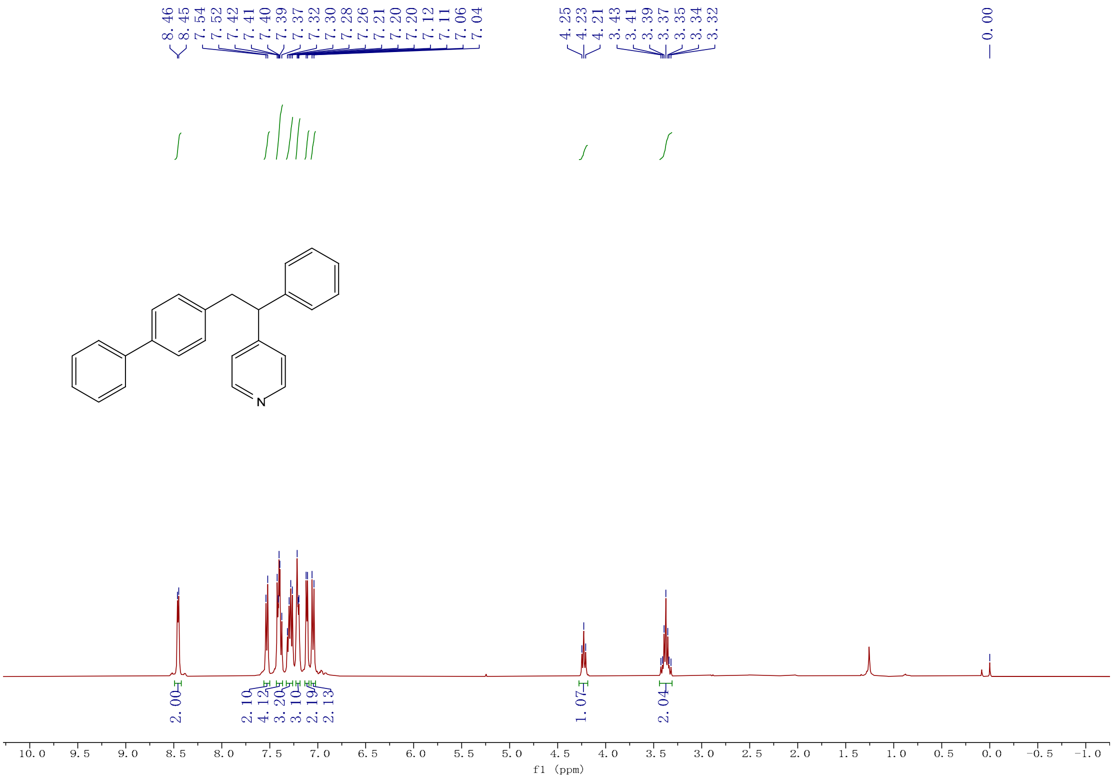

^{13}**C{**^{1}**H} NMR** -spectrum (**100**MHz, $CDCl_3$) of **5i**

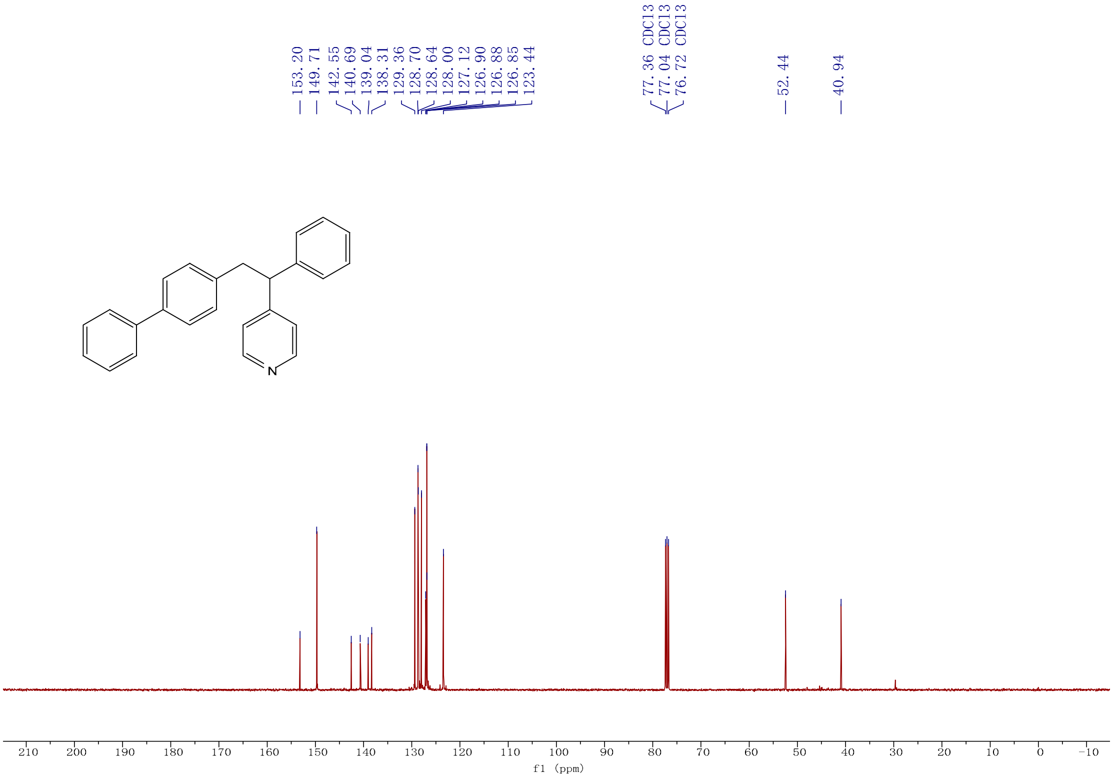

^{1}H NMR-spectrum (400MHz, $CDCl_3$) of **5j**

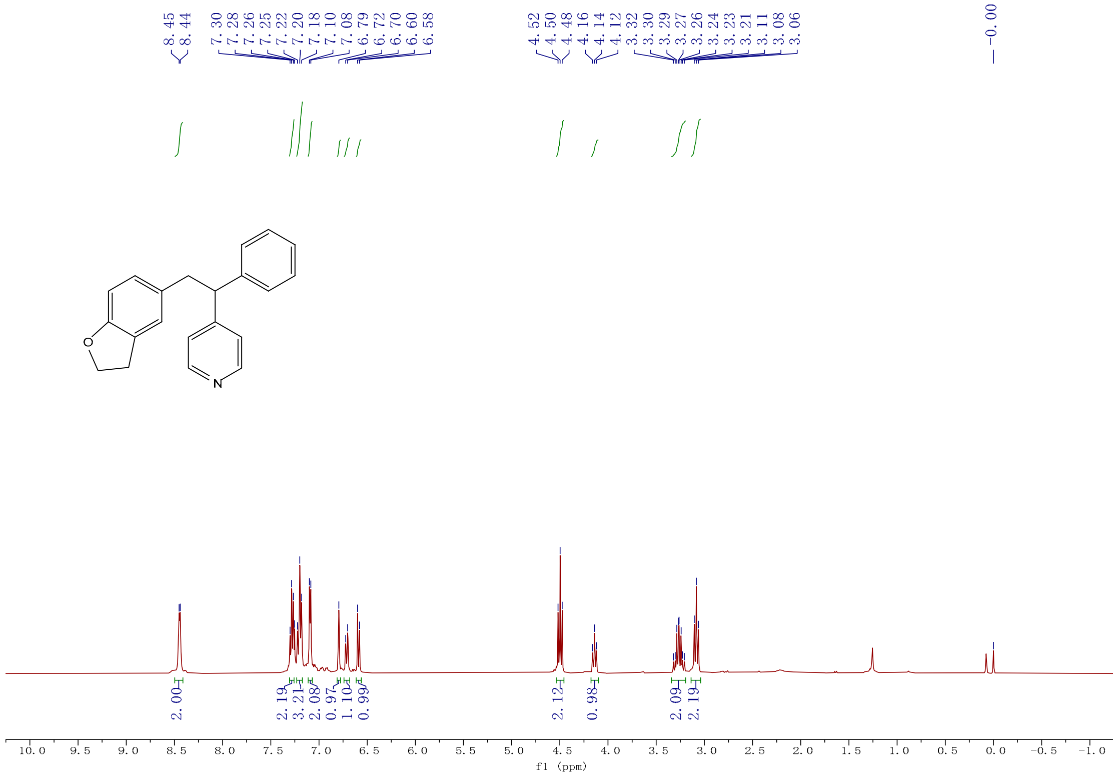

^{13}C{^{1}H} NMR -spectrum (**100**MHz, $CDCl_3$) of **5j**

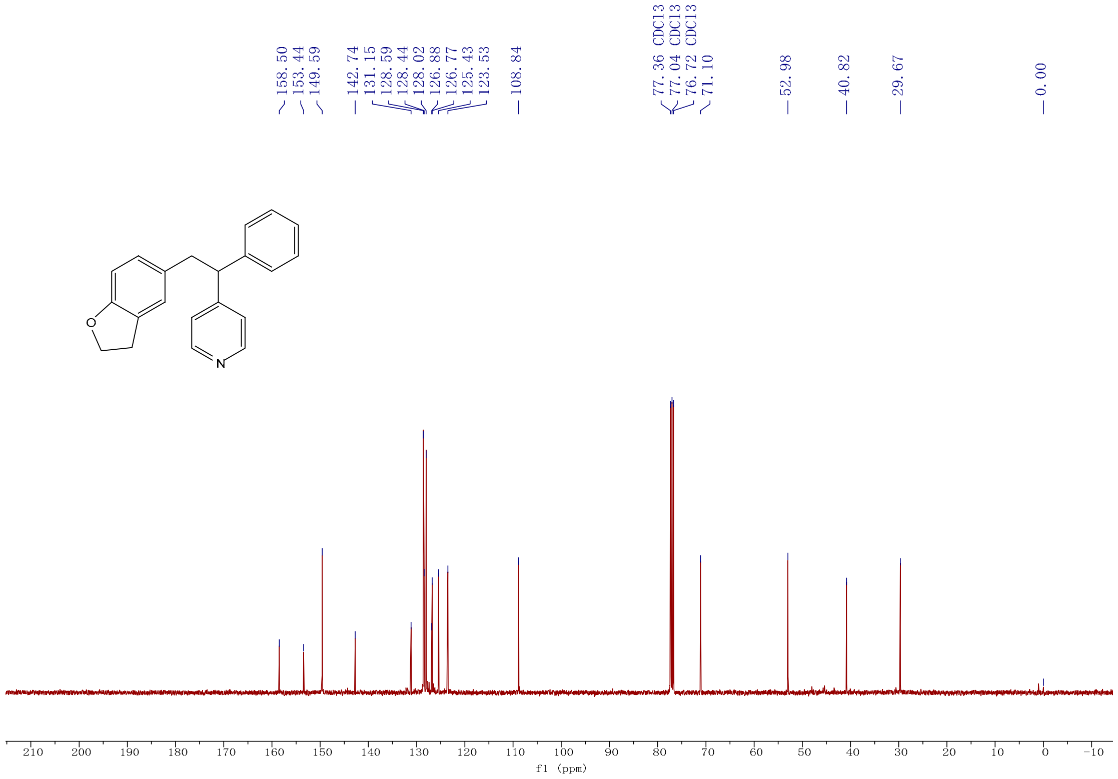

^{1}H NMR-spectrum (400MHz, $CDCl_3$) of **6a**

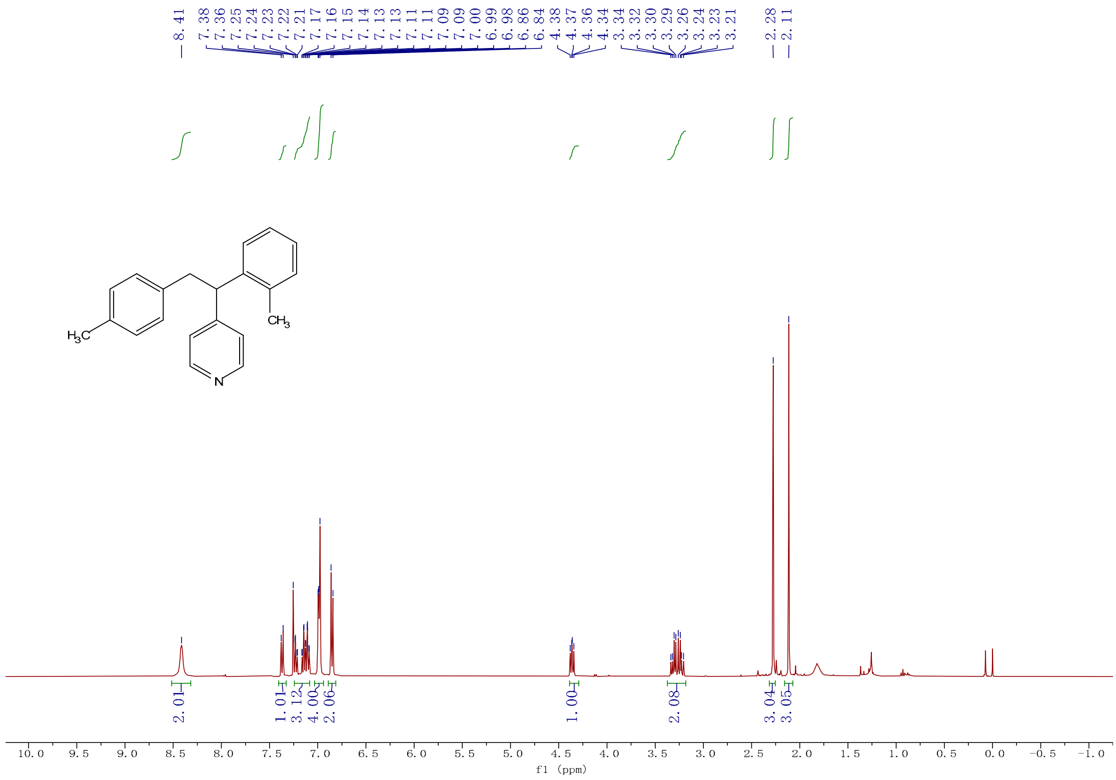

^{13}C{^{1}H} NMR -spectrum (**100**MHz, $CDCl_3$) of **6a**

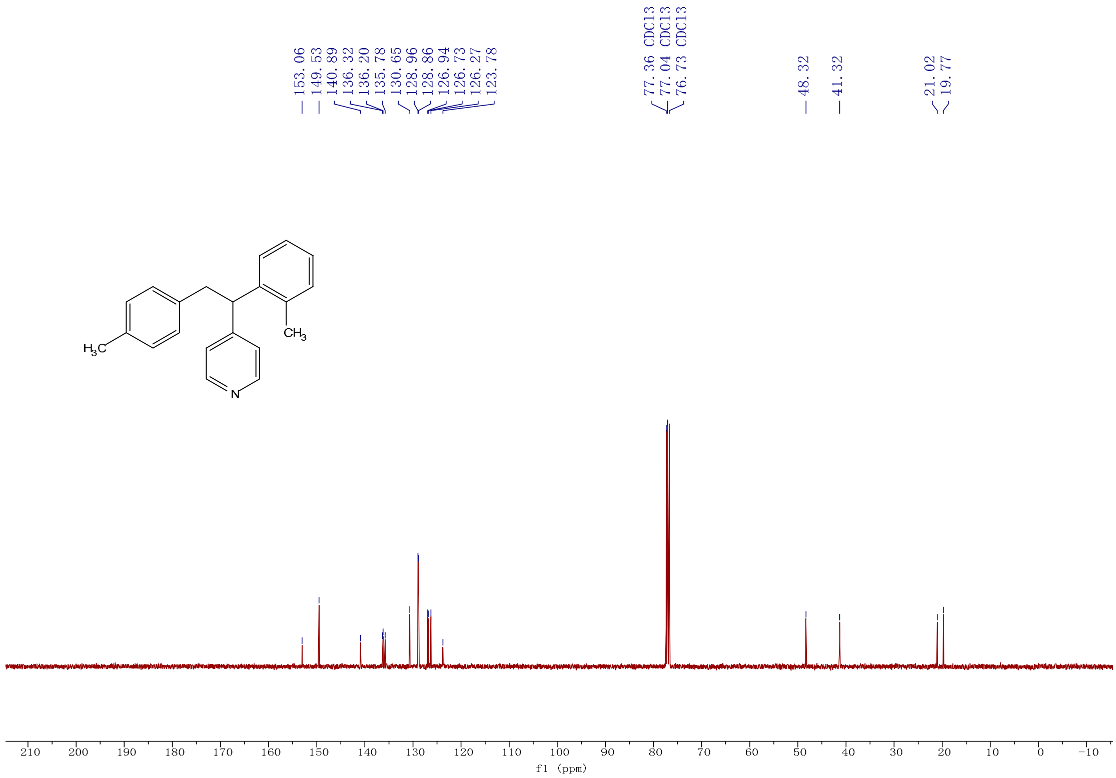

^{1}**H NMR**-spectrum (400MHz, $CDCl_3$) of **6b**

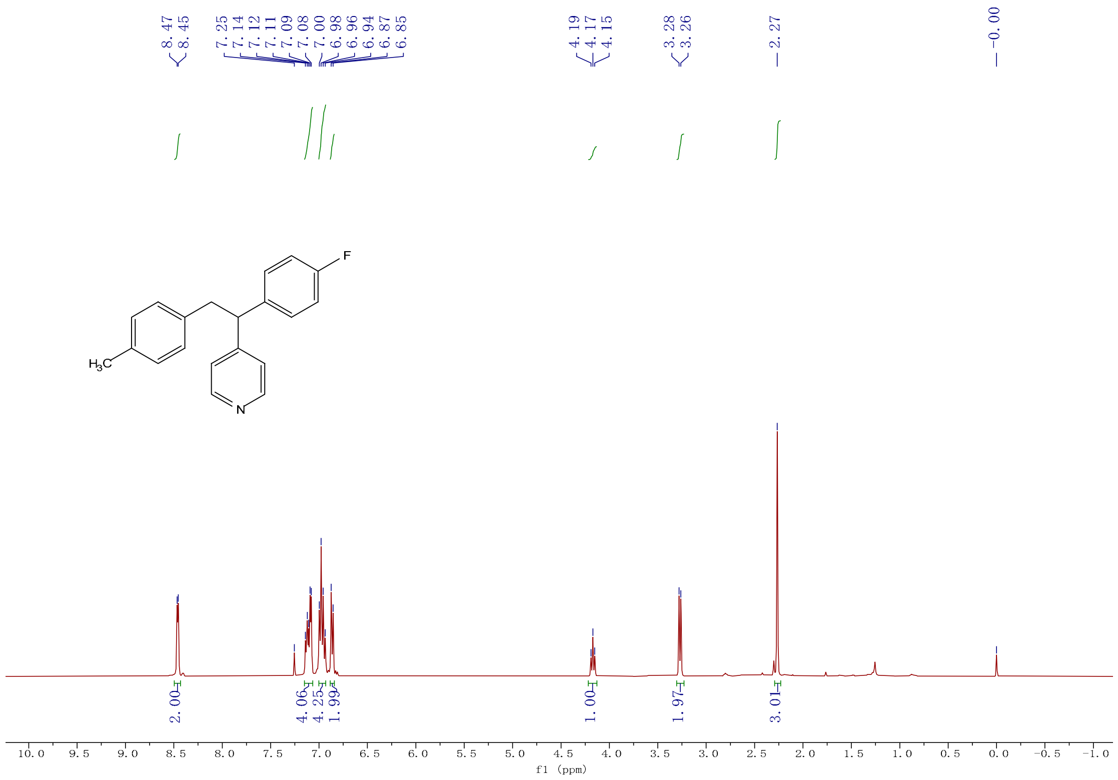

^{13}**C{**^{1}**H} NMR** -spectrum (**100**MHz, $CDCl_3$) of **6b**

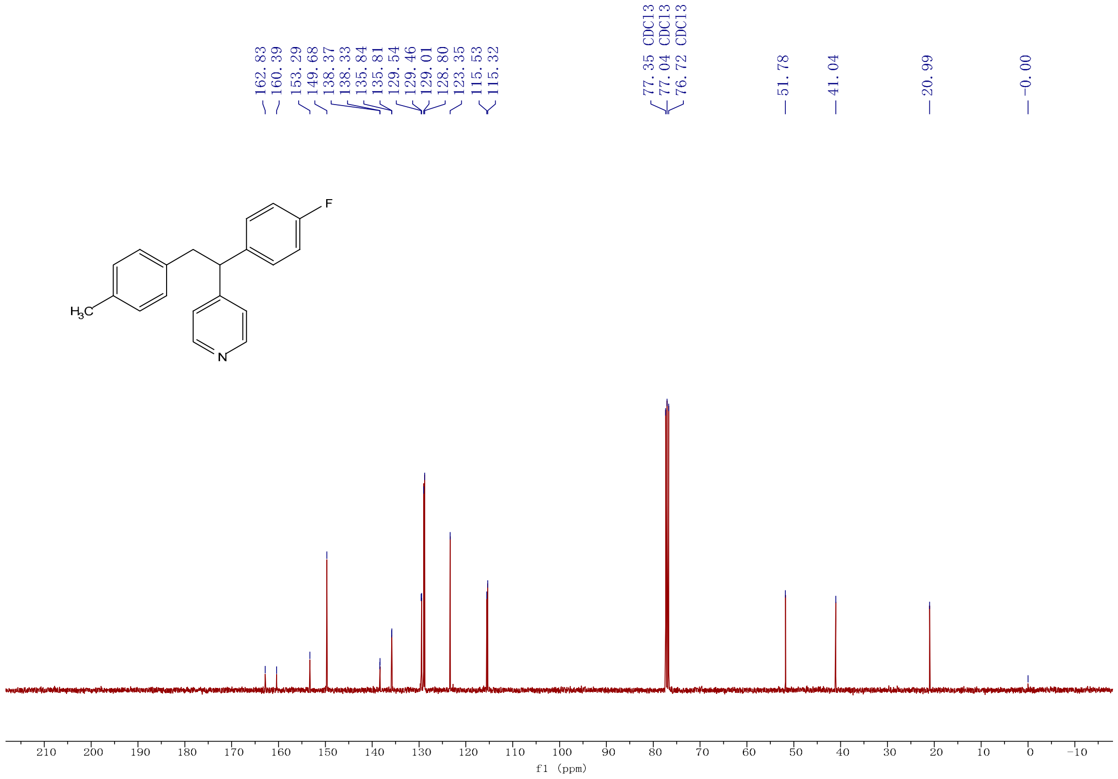

^{19}F NMR-spectrum (376 MHz, $CDCl_3$) of **6b**

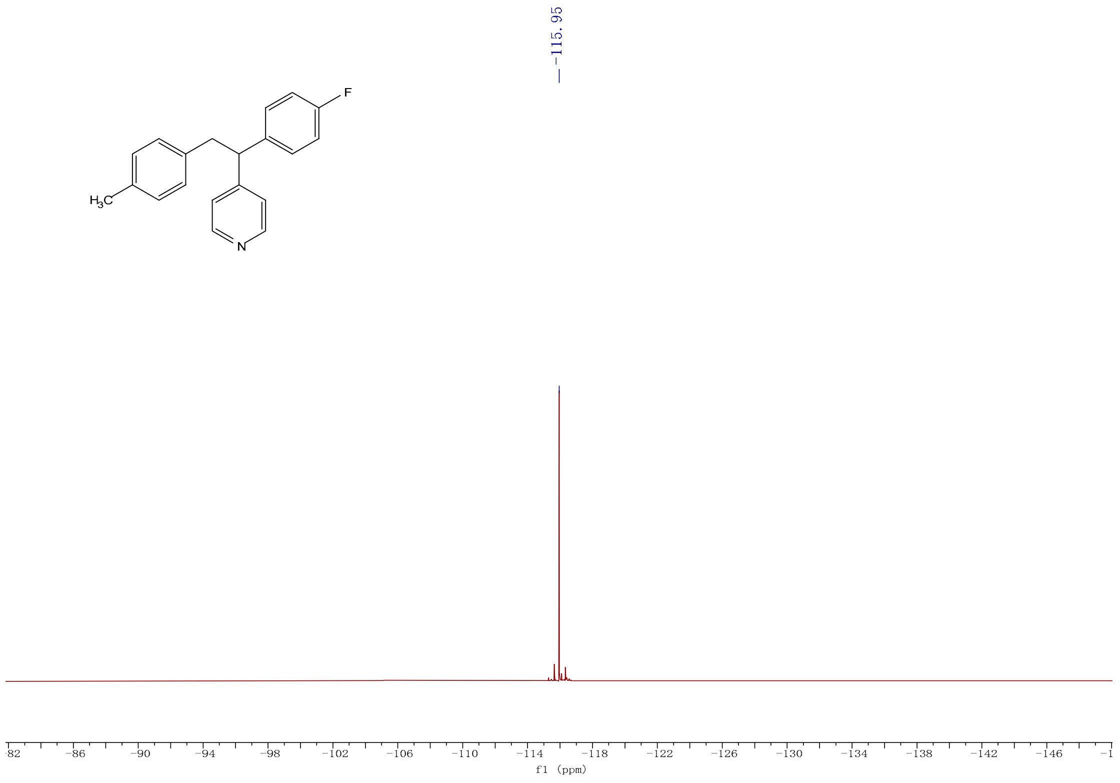

^{1}H NMR-spectrum (400MHz, $CDCl_3$) of **6c**

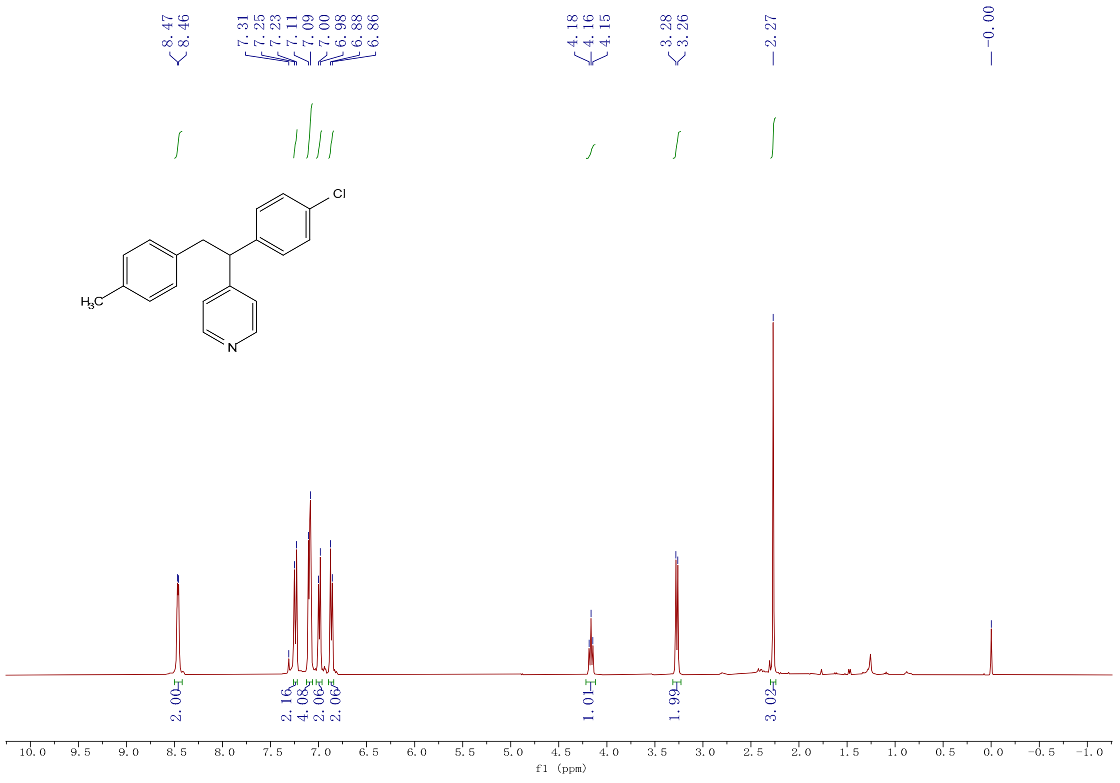

$^{13}C\{^{1}H\}$ NMR -spectrum (**100**MHz, $CDCl_3$) of **6c**

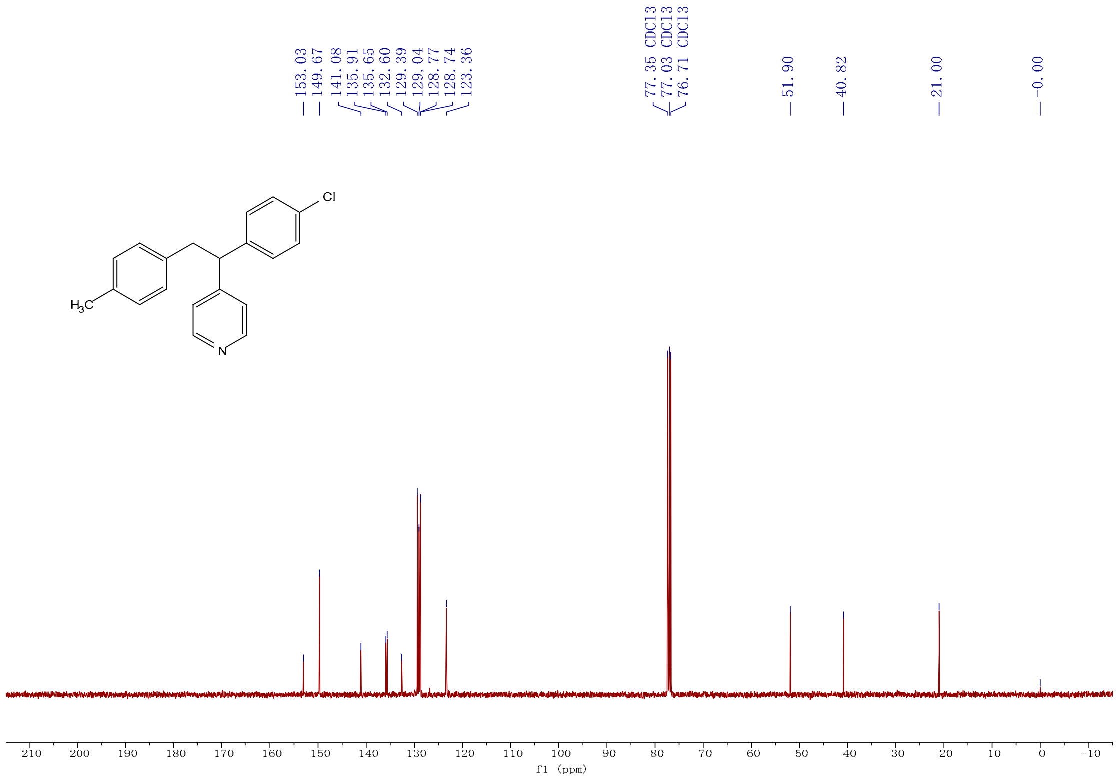

^{1}H NMR-spectrum (400MHz, $CDCl_3$) of **6d**

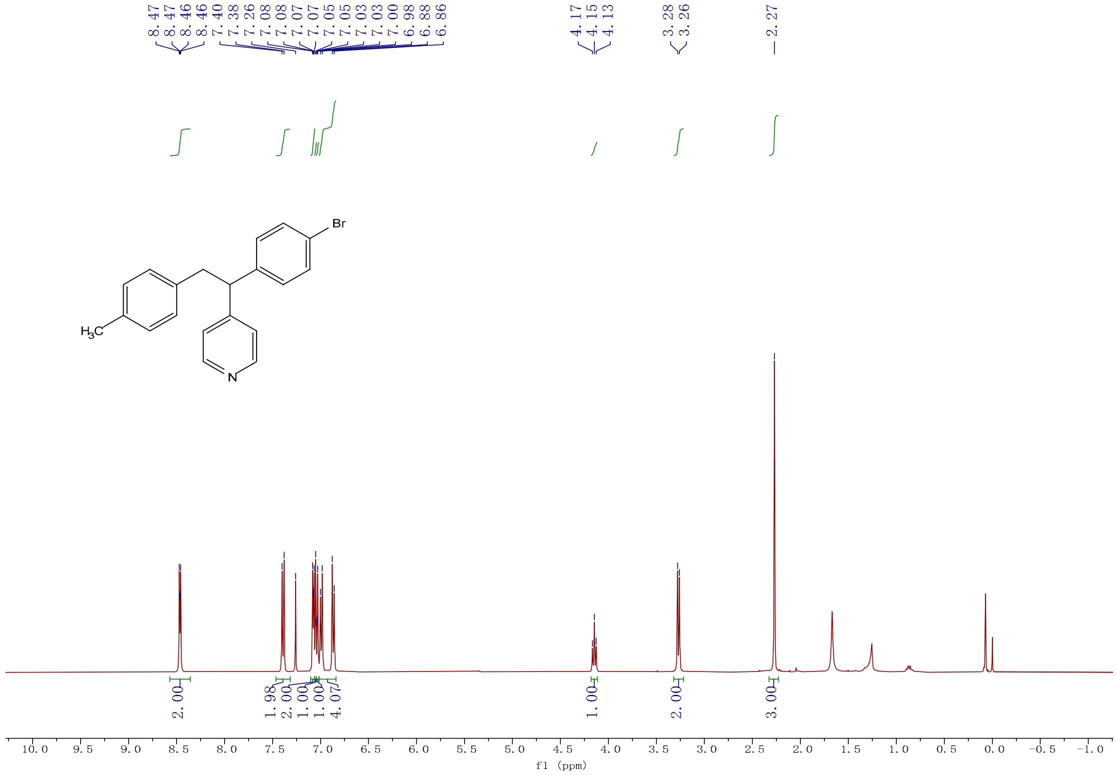

$^{13}C\{^1H\}$ **NMR** -spectrum (**100**MHz, $CDCl_3$) of **6d**

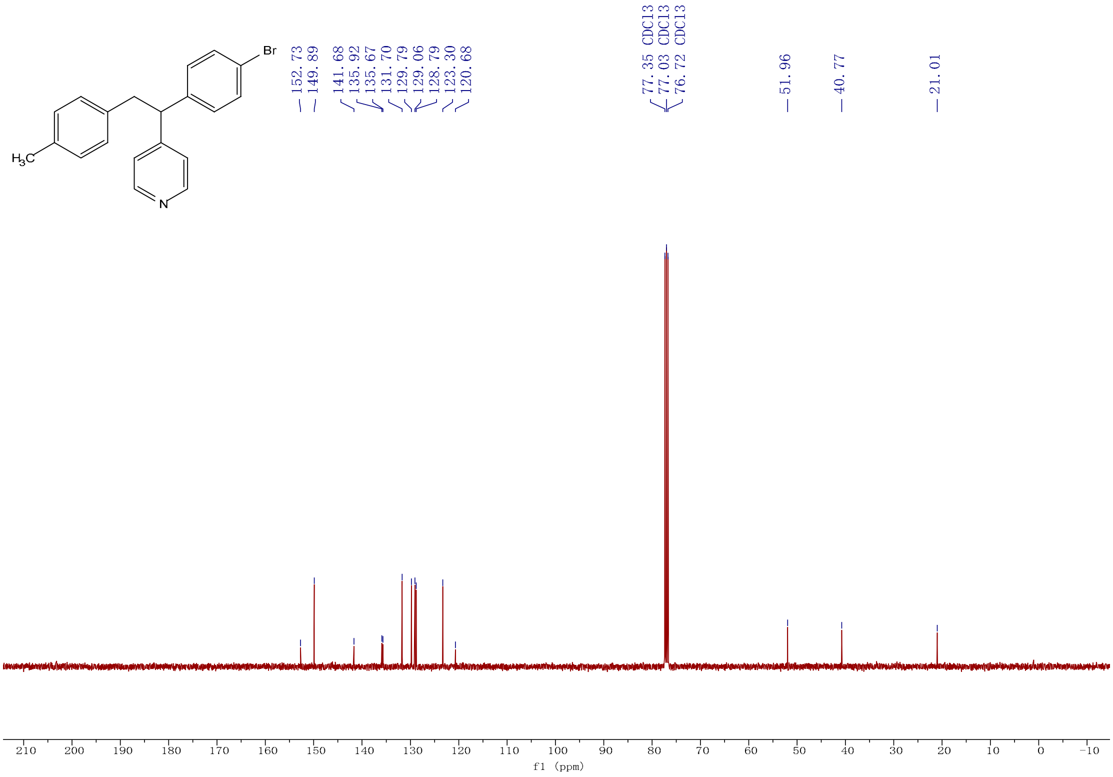

^{1}**H NMR**-spectrum (400MHz, $CDCl_3$) of **6e**

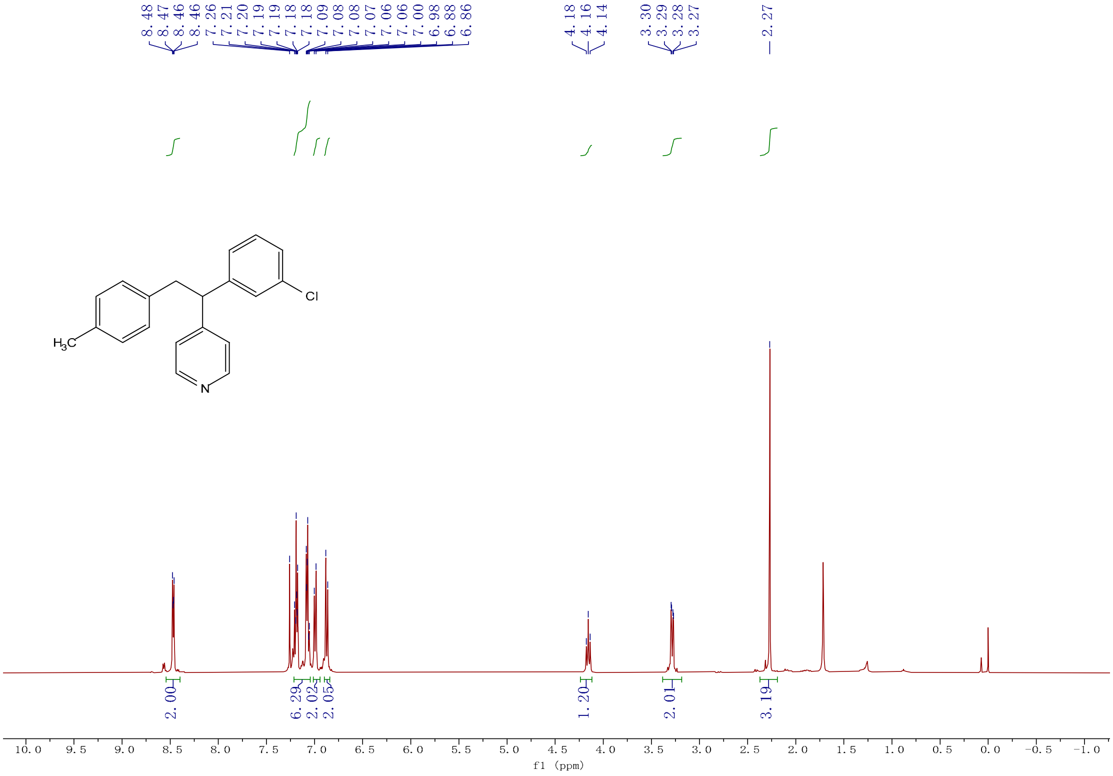

$^{13}C\{^{1}H\}$ NMR -spectrum (**100**MHz, $CDCl_3$) of **6e**

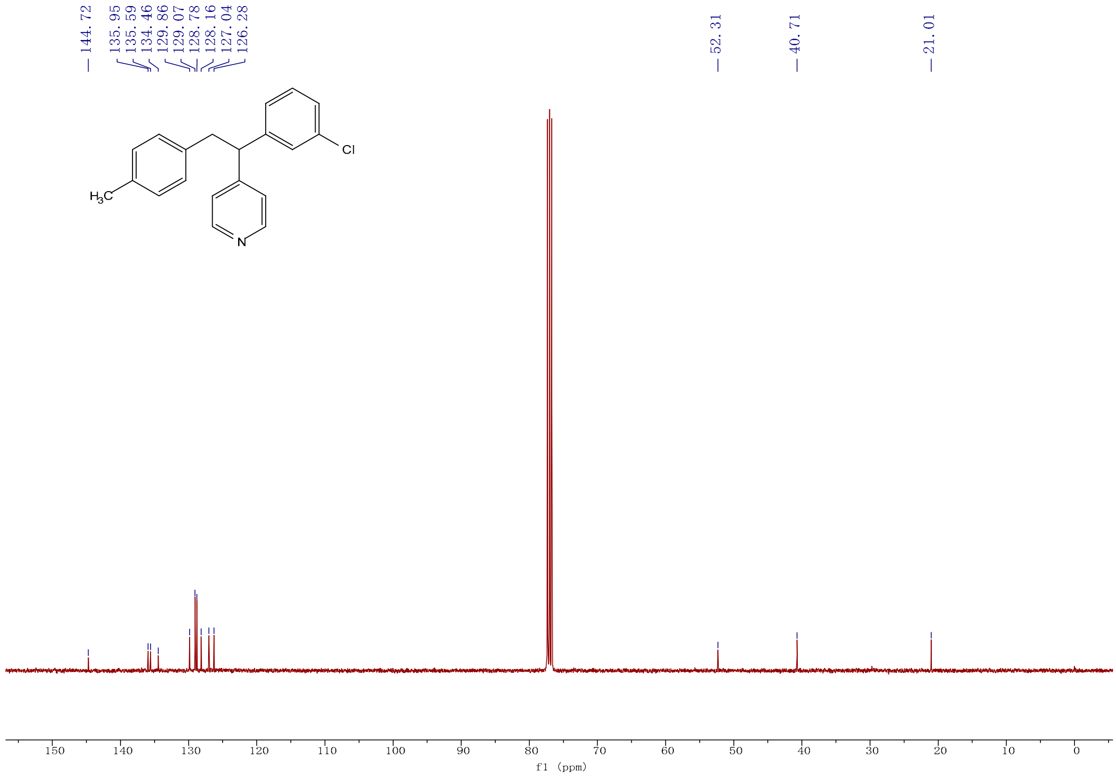

^{1}H NMR-spectrum (400MHz, $CDCl_3$) of **6f**

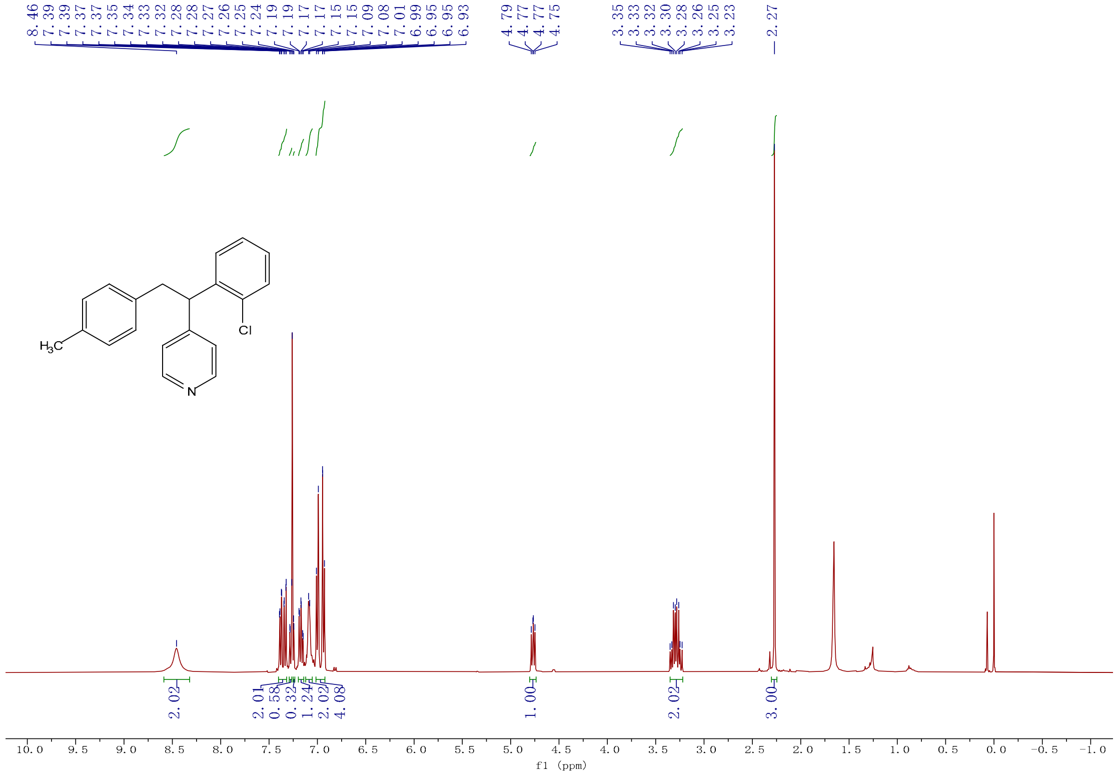

$^{13}C\{^{1}H\}$ **NMR** -spectrum (**100**MHz, $CDCl_3$) of **6f**

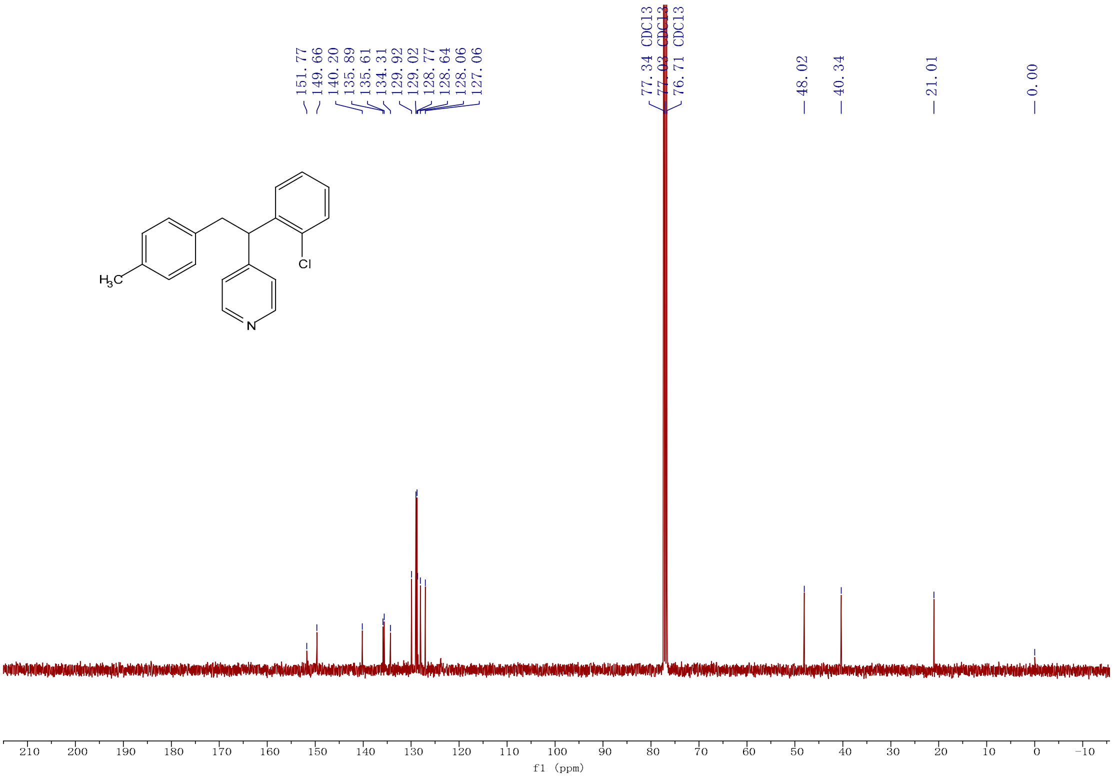

^{1}**H NMR**-spectrum (400MHz, $CDCl_3$) of **6g**

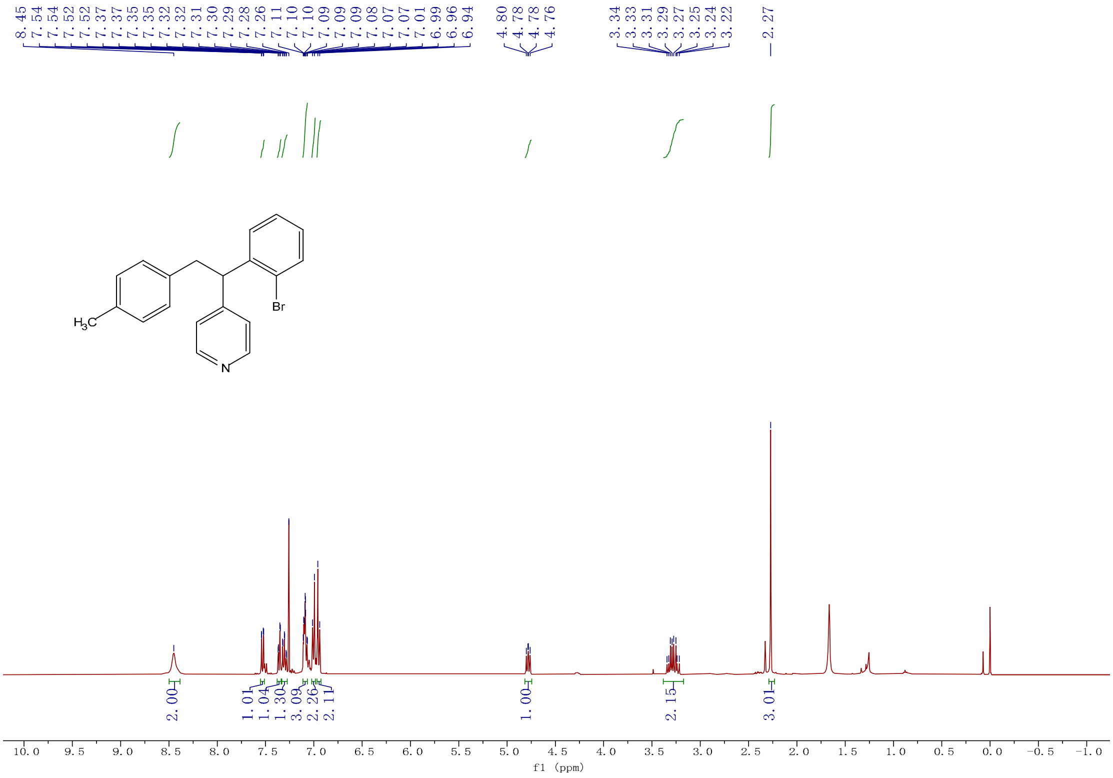

$^{13}C\{^{1}H\}$ NMR -spectrum (**100**MHz, $CDCl_3$) of **6g**

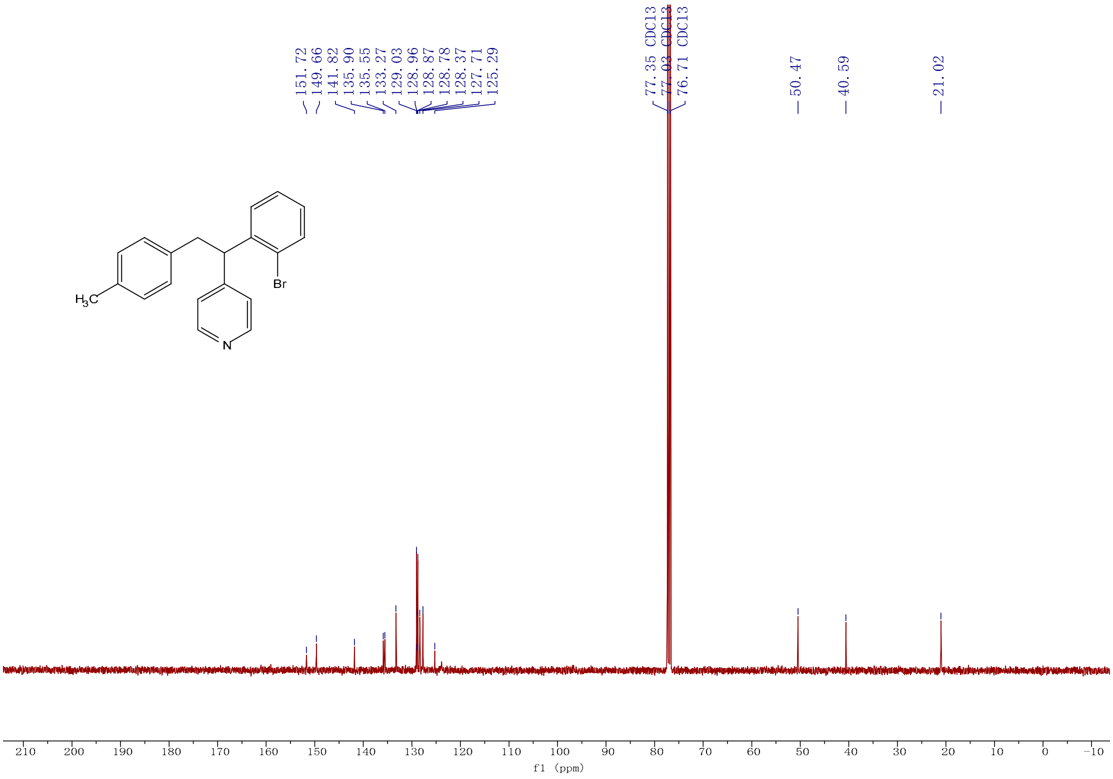

^{1}H NMR-spectrum (400MHz, $CDCl_3$) of **6h**

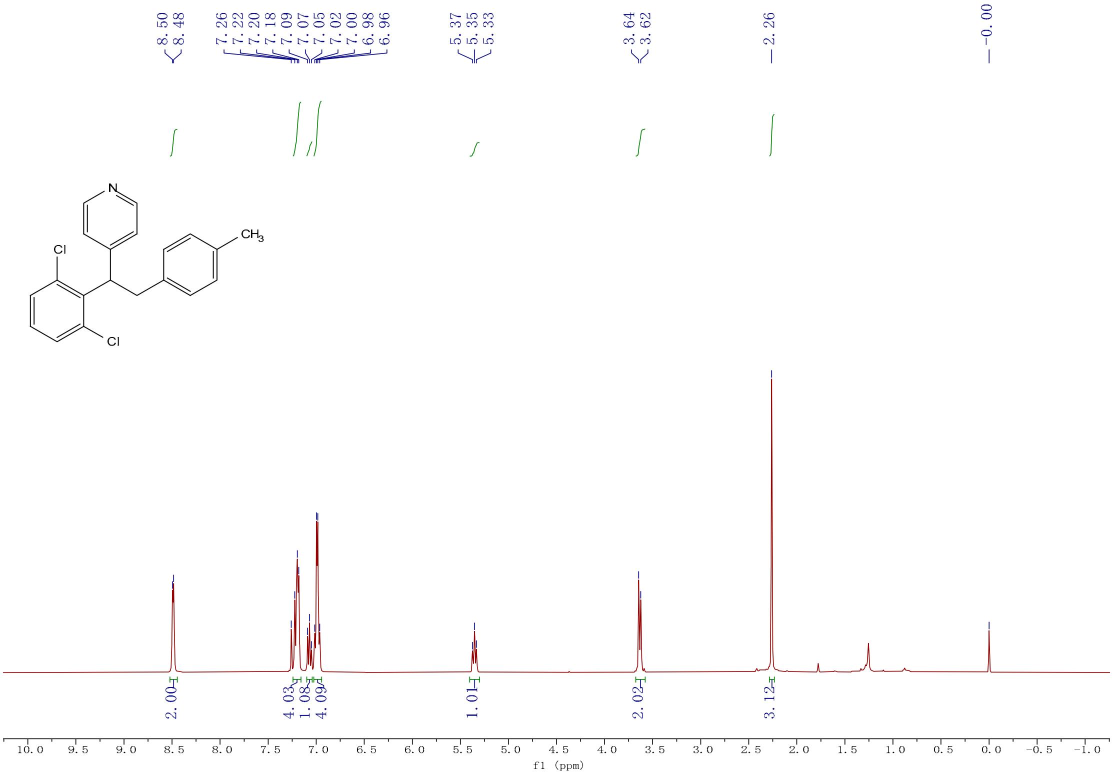

$^{13}C\{^{1}H\}$ NMR -spectrum (**100**MHz, $CDCl_3$) of **6h**

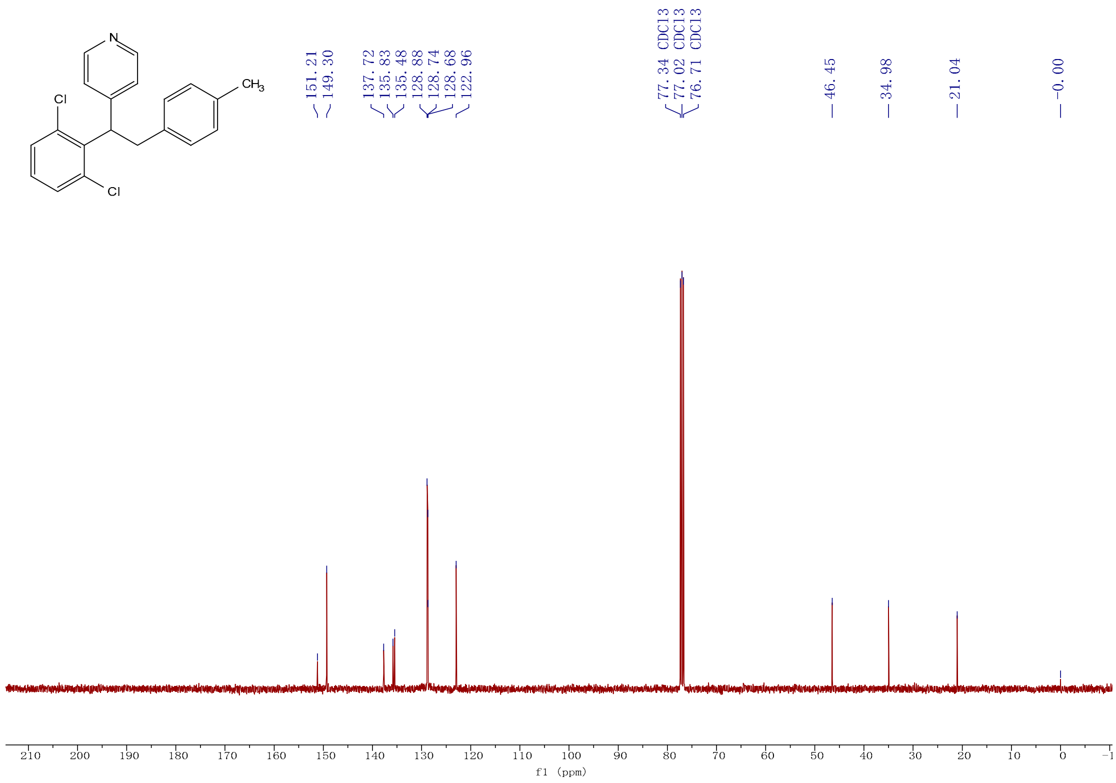

^{1}H NMR-spectrum (400MHz, $CDCl_3$) of **6i**

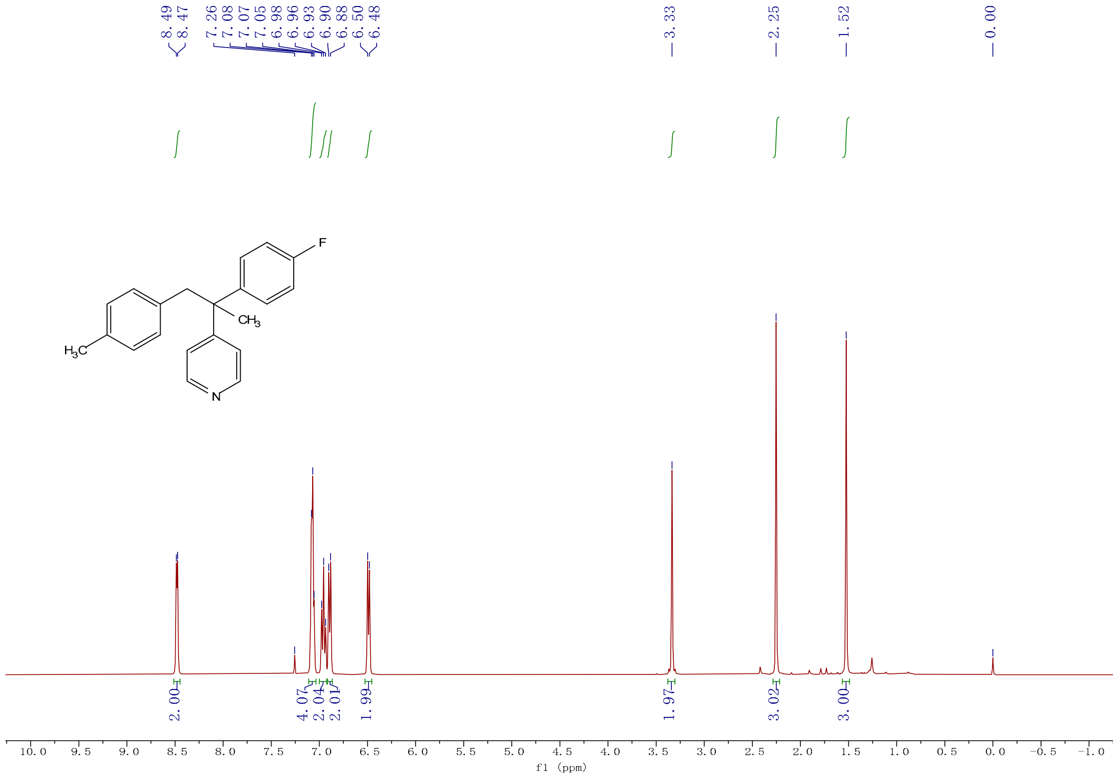

$^{13}C\{^{1}H\}$ **NMR** -spectrum (**100**MHz, $CDCl_3$) of **6i**

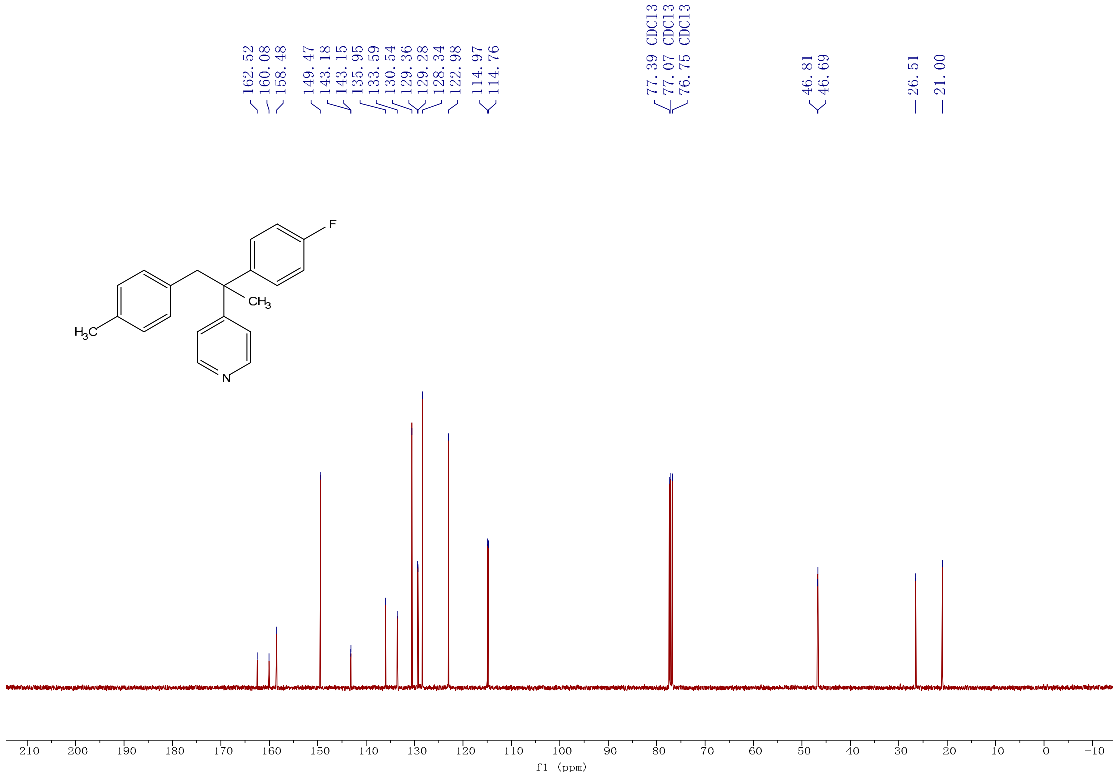

^{19}F **NMR**-spectrum (376 MHz, $CDCl_3$) of **6i**

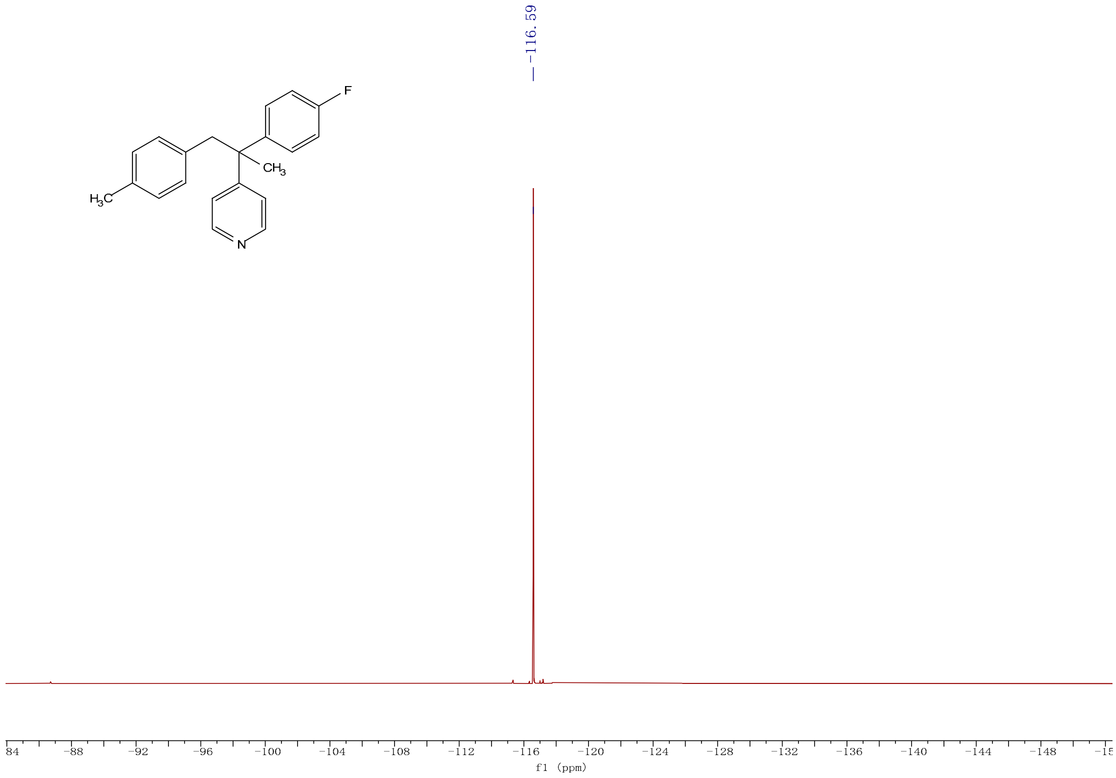

^{1}H NMR-spectrum (400MHz, $CDCl_3$) of **6j**

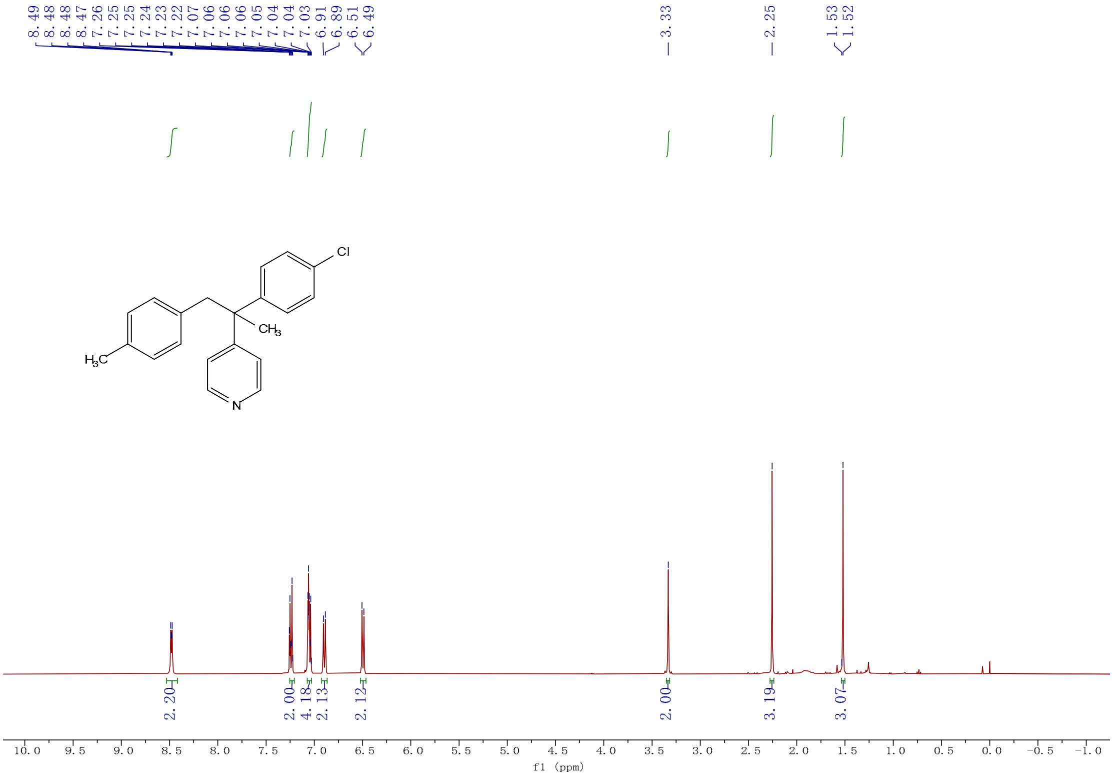

^{13}C{^{1}H} NMR -spectrum (**100**MHz, $CDCl_3$) of **6j**

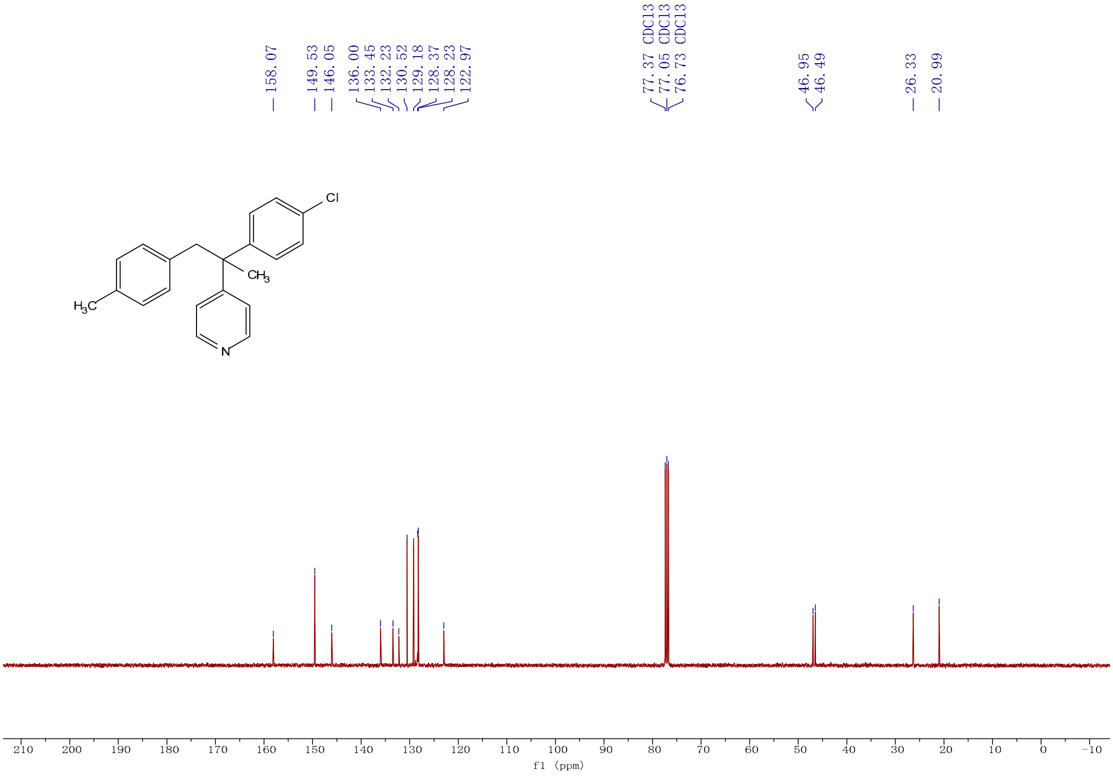

^{1}**H NMR**-spectrum (400MHz, $CDCl_3$) of **6k**

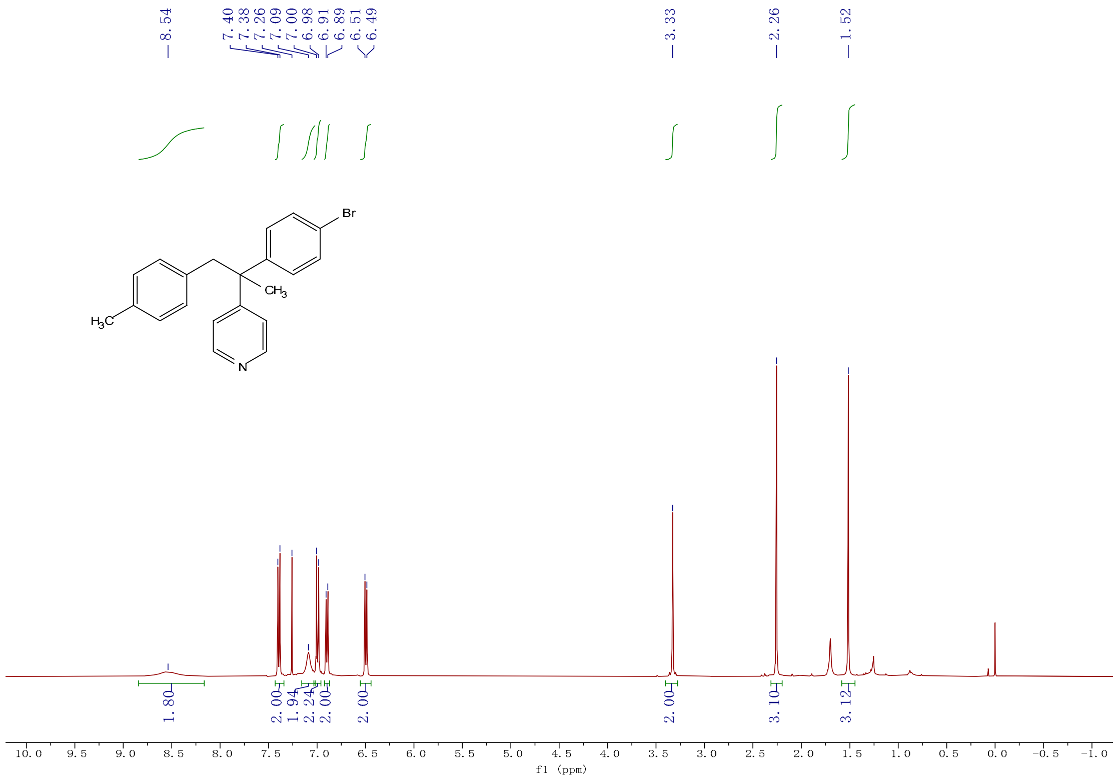

^{13}**C{**^{1}**H} NMR** -spectrum (**100**MHz, $CDCl_3$) of **6k**

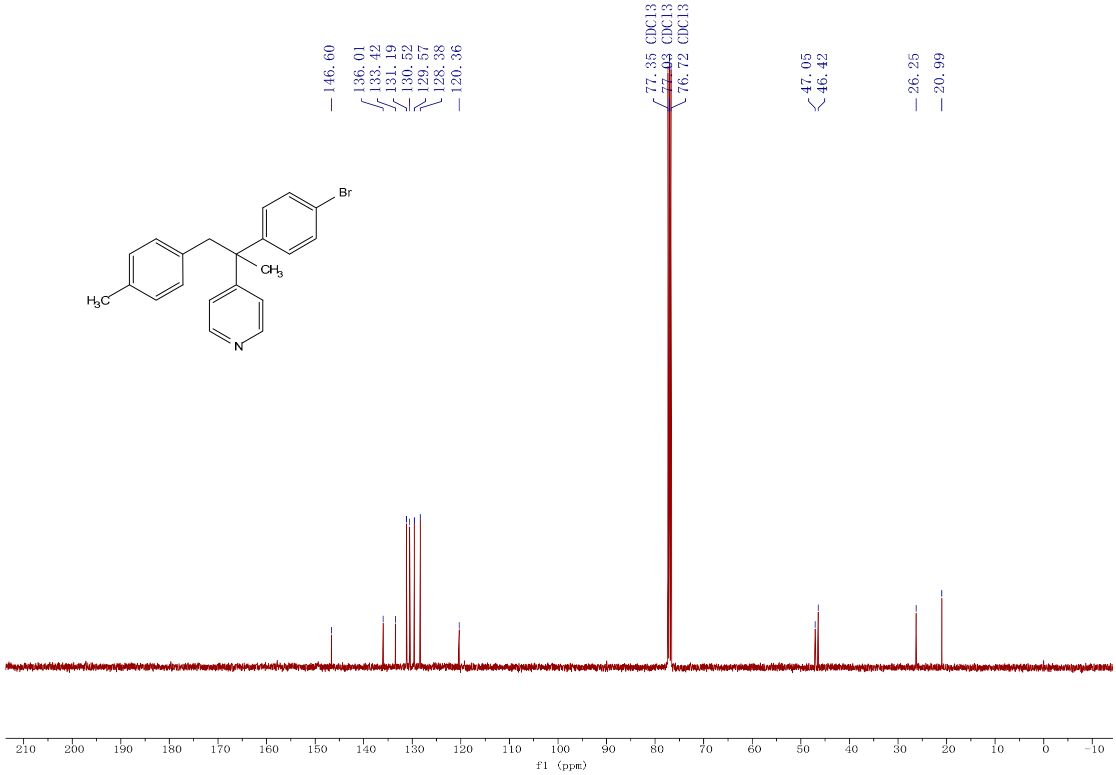

^{1}H NMR-spectrum (400MHz, $CDCl_3$) of **6l**

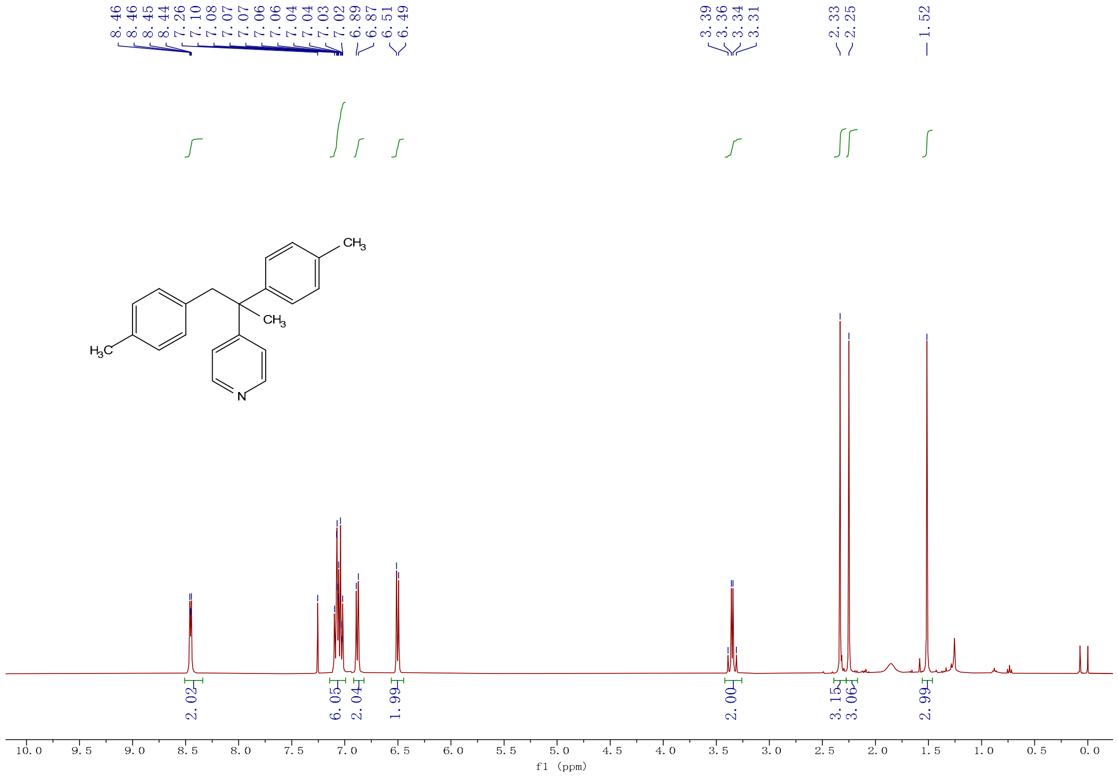

^{13}C{^{1}H} NMR -spectrum (**100**MHz, $CDCl_3$) of **6l**

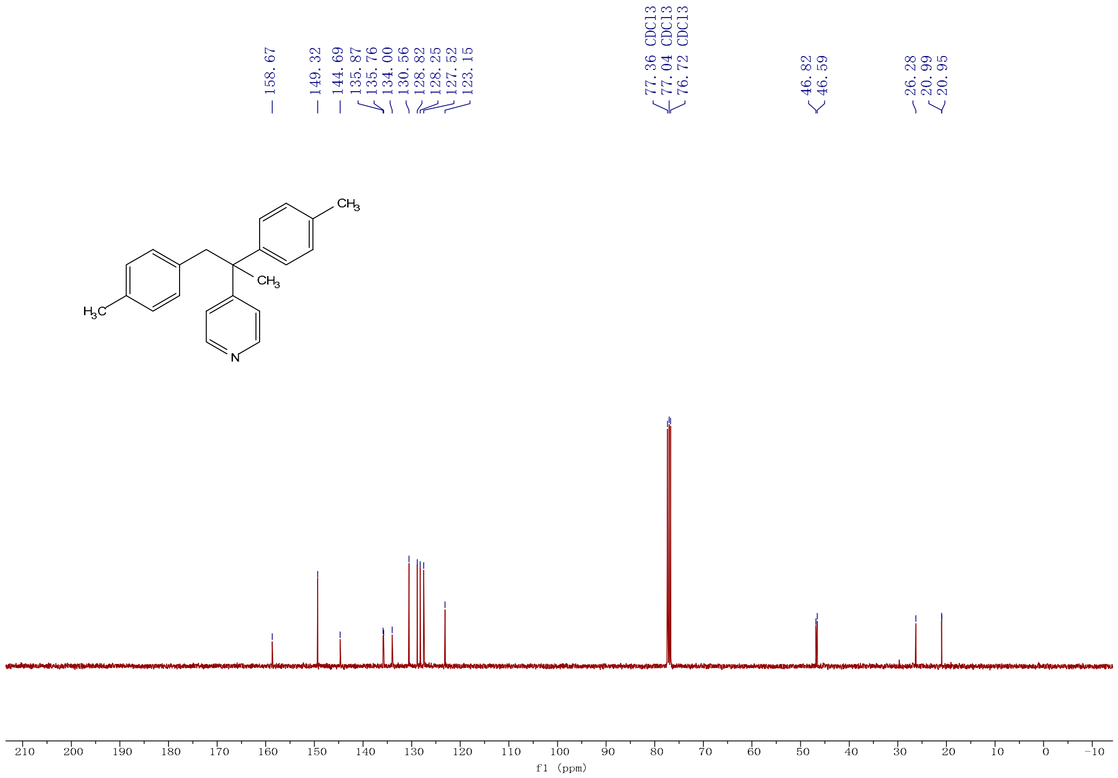

^{1}H NMR-spectrum (400MHz, $CDCl_3$) of **6m**

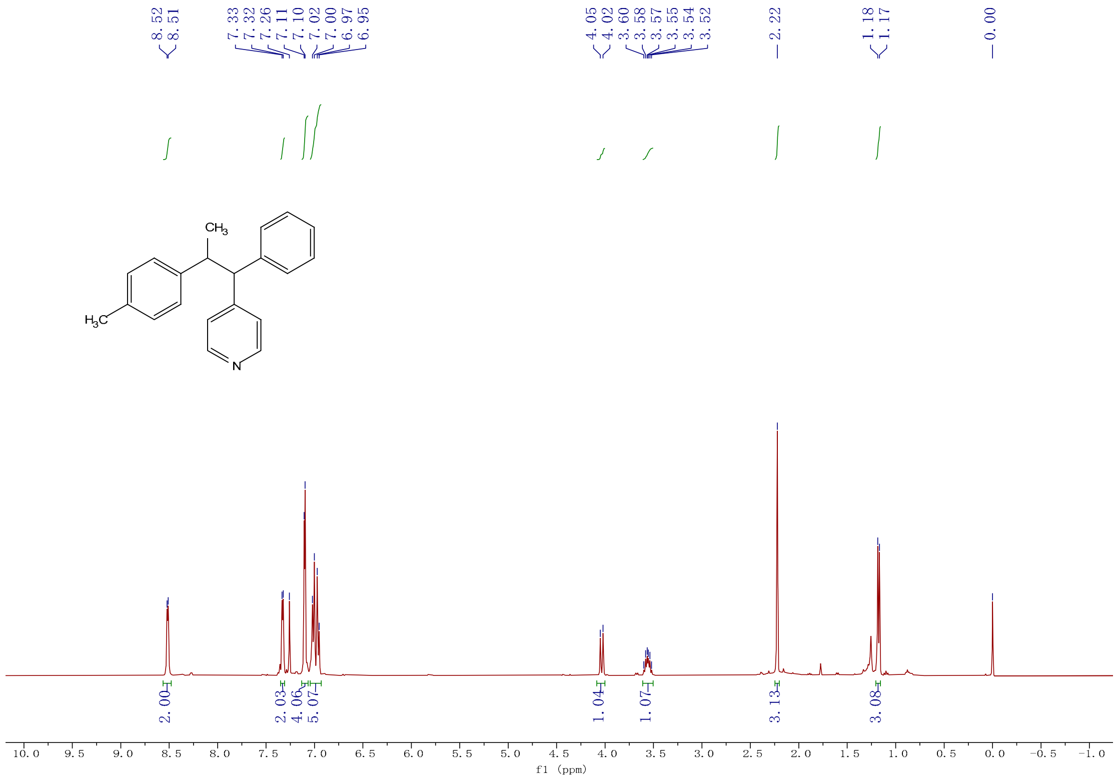

^{13}C{^{1}H} NMR -spectrum (**100**MHz, $CDCl_3$) of **6m**

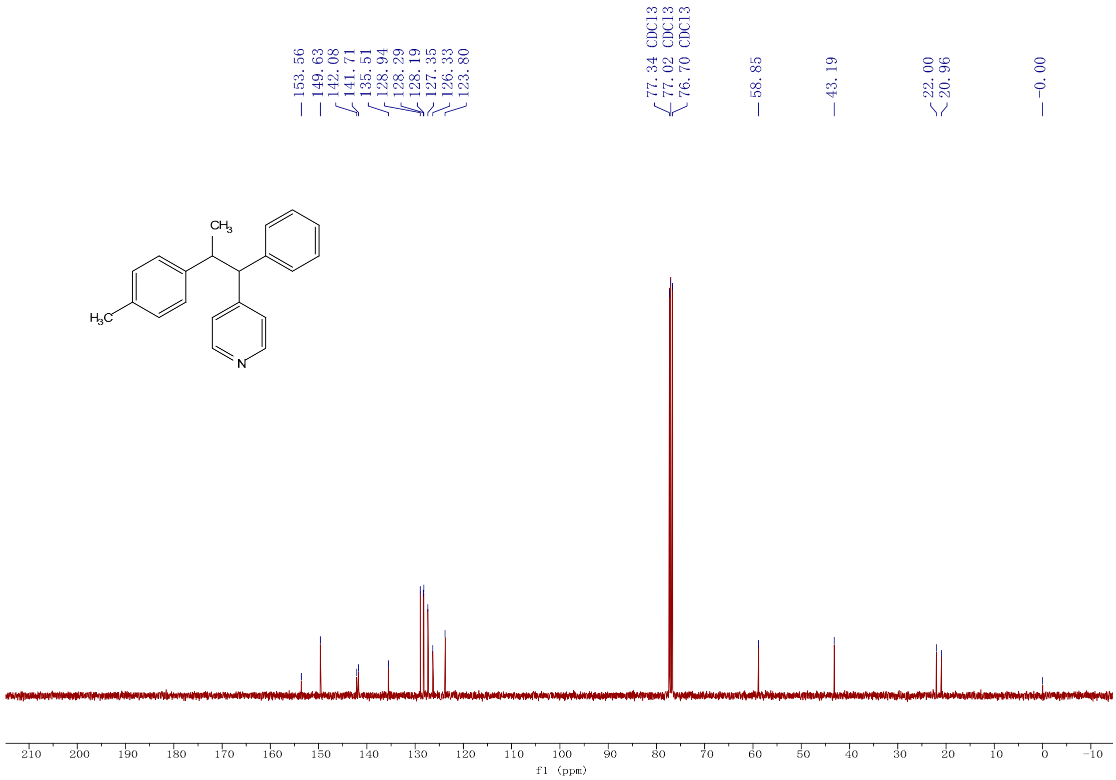

^{1}H NMR-spectrum (400MHz, $CDCl_3$) of **6n**

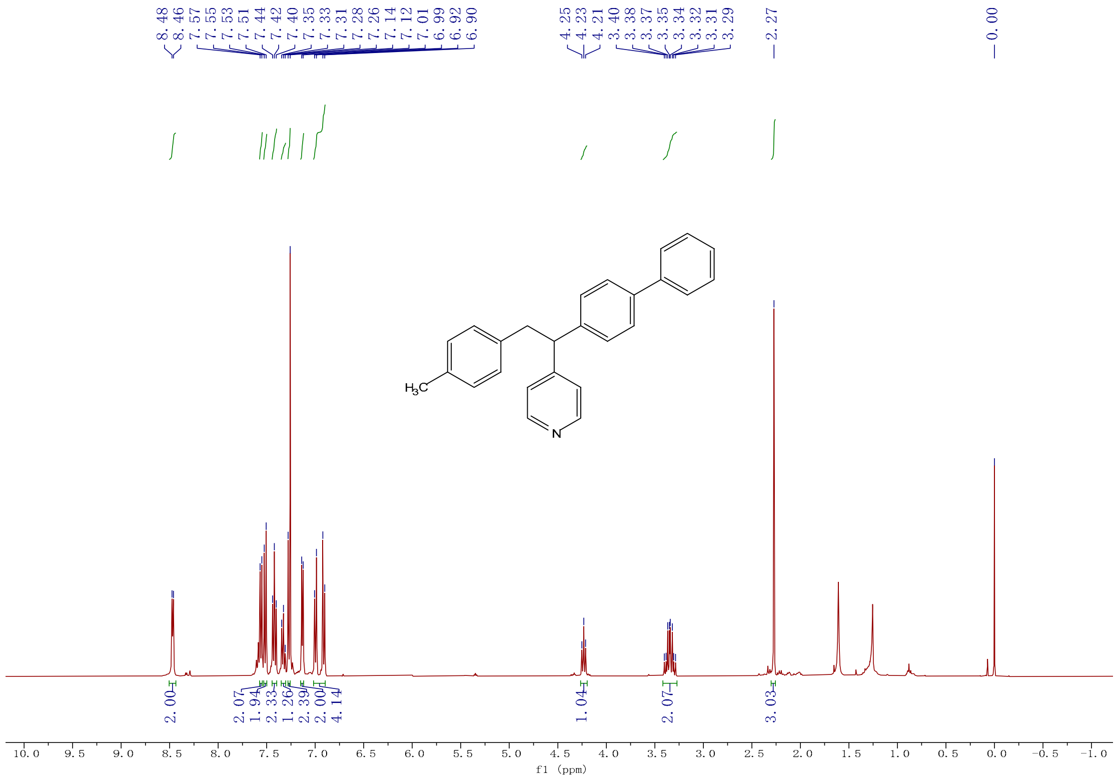

^{13}C{^{1}H} NMR -spectrum (**100**MHz, $CDCl_3$) of **6n**

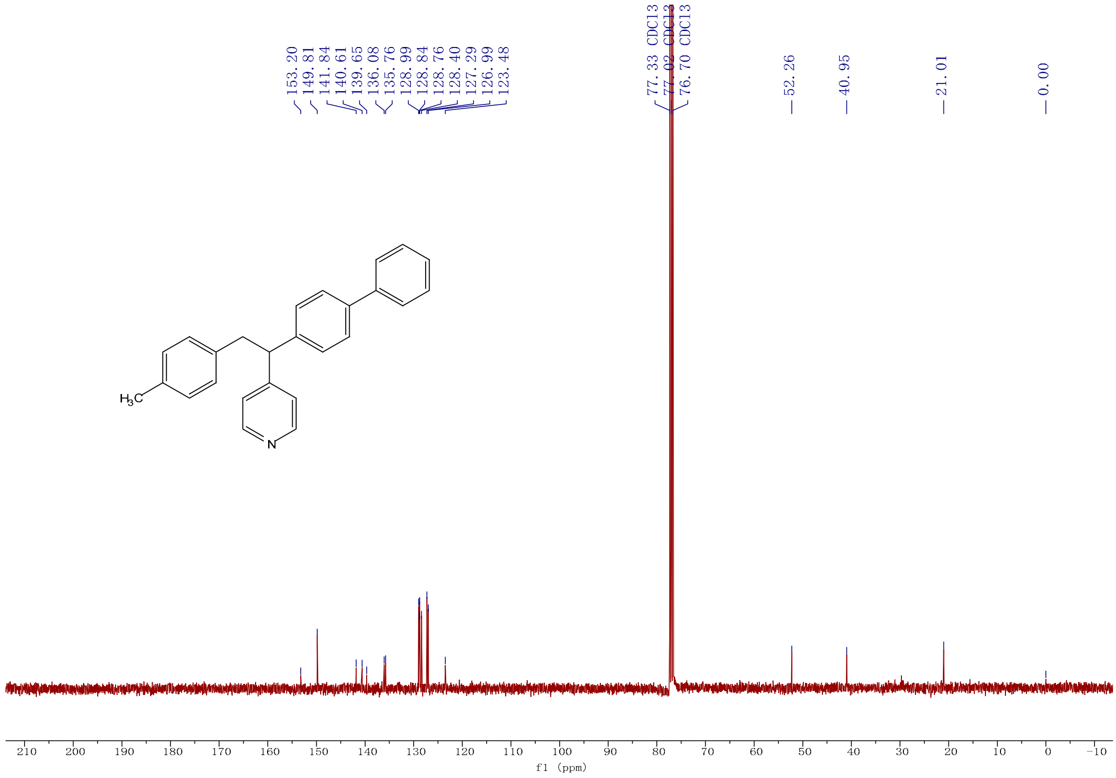

^{1}H NMR-spectrum (400MHz, $CDCl_3$) of **9**

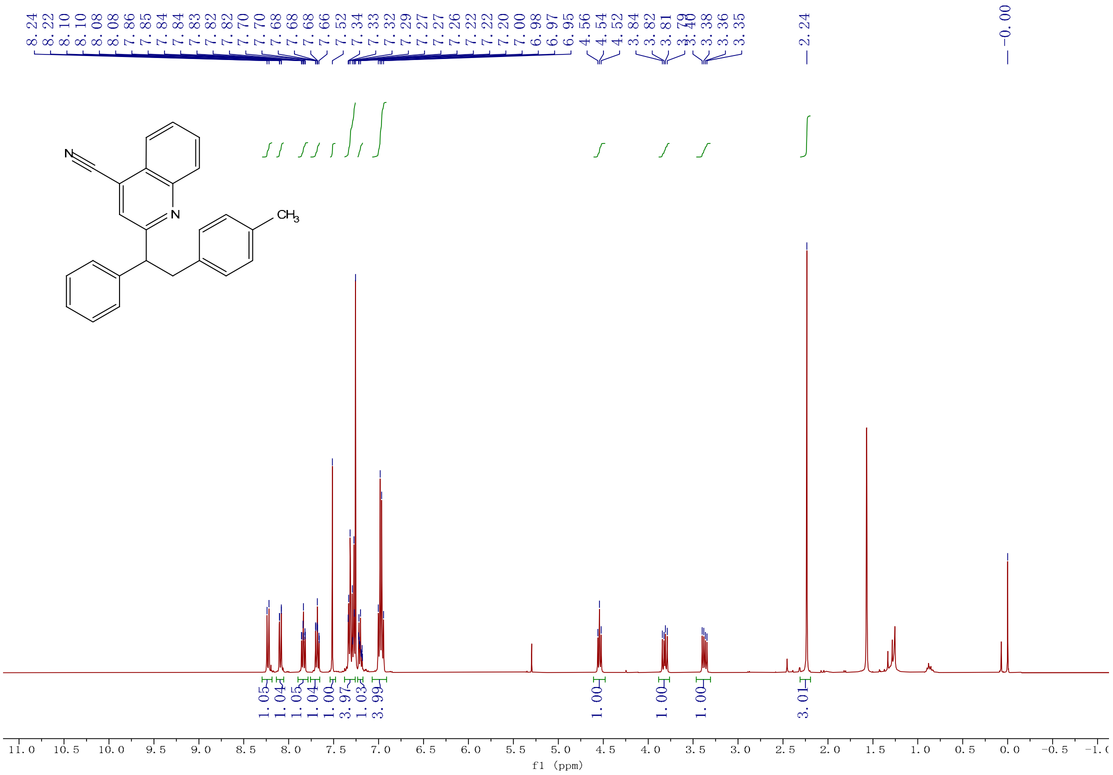

^{13}C{^{1}H} NMR -spectrum (**100**MHz, $CDCl_3$) of **9**

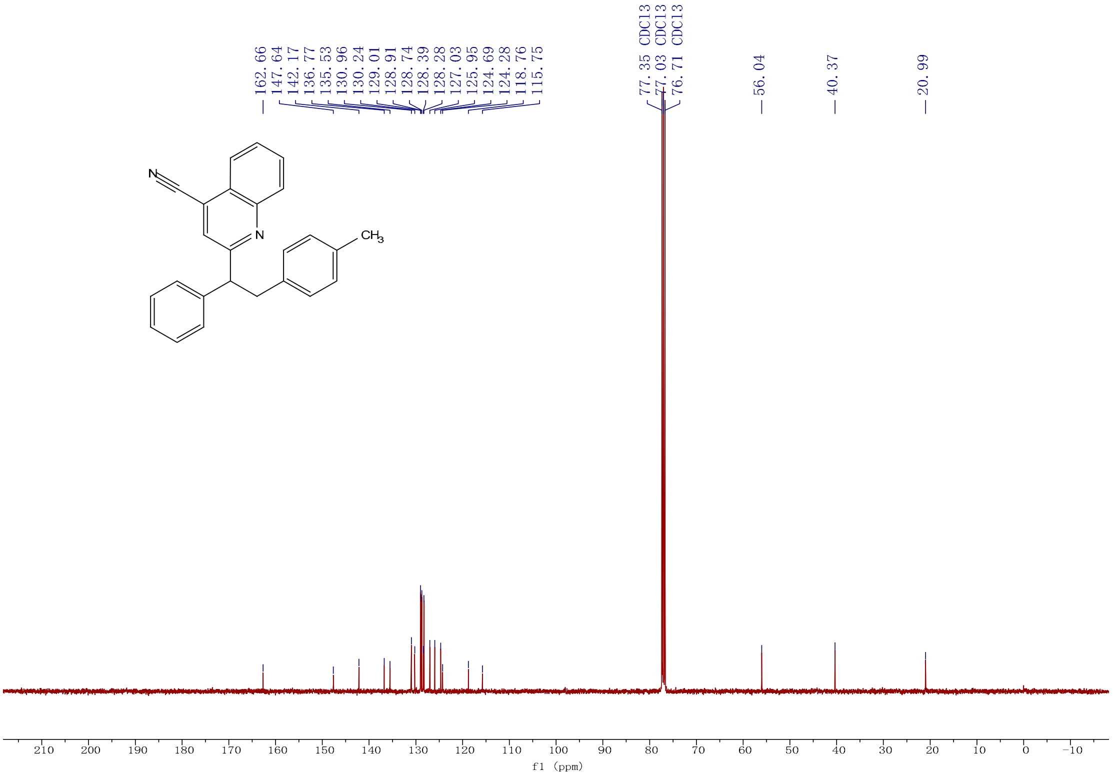

Supplement: Supplementary file 1 [file molecules-31-02216-s001.zip › molecules-4376343-supplementary.pdf]
